# Identification of novel gut microbiota-related biomarkers in cerebral hemorrhagic stroke

**DOI:** 10.3389/fmed.2025.1636860

**Published:** 2025-08-26

**Authors:** Fengli Ye, Huili Li, Hongying Li, Xiue Mu

**Affiliations:** Department of Anesthesiology, The First Hospital of Hebei Medical University, Shijiazhuang, China

**Keywords:** cerebral hemorrhagic stroke, gut microbe, bioinformatics analysis, hub genes, fecal microbiota transplantation

## Abstract

**Introduction:**

Hemorrhagic stroke, especially intracerebral hemorrhage (ICH), is the most fatal type of stroke and a major cause of mortality and disability. Due to ambiguous symptoms and limited biomarkers, early diagnosis and prognosis remain challenging. Recent evidence suggests that gut microbiota dysregulation influences neuroinflammation and outcomes in ICH, but the underlying molecular mechanisms remain unclear.

**Methods:**

Transcriptome data from the GSE24265 dataset were analyzed to identify differentially expressed genes (DEGs) in ICH. Gut microbiota-related genes (GMRGs) were obtained from GeneCards and literature, and overlapping genes were defined as gut microbiota-related DEGs (GMRDEGs). Functional enrichment, gene set enrichment analysis (GSEA), and protein–protein interaction (PPI) analyses were performed. Hub genes were screened using LASSO, RandomForest, and SVM-RFE algorithms. Validation was conducted in plasma samples from ICH patients (n=20) and controls (*n* < 20) by qRT-PCR, and in a collagenase-induced ICH mouse model. The therapeutic effect of fecal microbiota transplantation (FMT) was evaluated through neurological scoring, hematoma volume, brain edema, intestinal barrier protein expression, inflammatory cytokines, and hub gene expression.

**Results:**

We identified 806 DEGs in ICH, among which 65 overlapped with GMRGs. These GMRDEGs were enriched in immune processes and pathways such as TNF and IL-17 signaling. PPI network analysis highlighted IL1B, IL6, and CCL2 as central nodes. Machine learning identified four hub genes—LEF1, ITGAX, BLVRB, and ATF4. All were significantly upregulated in ICH tissues and plasma, correlating with immune cell infiltration. In vivo, FMT reduced hematoma volume and brain edema, improved neurological function, restored intestinal barrier proteins, decreased inflammatory cytokines, and downregulated hub gene expression.

**Discussion:**

LEF1, ITGAX, BLVRB, and ATF4 were identified as gut microbiota-related biomarkers of ICH. Their modulation by FMT highlights the role of the brain–gut axis in ICH and suggests potential diagnostic biomarkers and therapeutic targets.

## Introduction

Stroke is the second leading cause of disability and death worldwide (1). Stroke is mainly classified into the ischemic stroke (IS) and hemorrhagic stroke. Hemorrhagic stroke as the most lethal type of stroke, and it is mainly divided into intracerebral hemorrhage (ICH) and subarachnoid hemorrhage (SAH). In 2016, there were 13.7 million new strokes cases globally; approximately 87% of these were IS (2). In China, in 2020, the reported stroke cases exceeded 3.4 million, of which 81.9% were IS and 14.9% were ICH (3). ICH is a subtype of stroke, mainly related to aging and hypertension. Currently, the diagnosis of ICH mainly relies on neuroimaging examinations, but clinicians still have difficulties in differentiating ICH from acute IS because its symptoms are rather ambiguous. Therefore, it is necessary to explore and determine potential biomarkers related to ICH to understand the molecular basis of ICH and improve the diagnostic efficiency.

Gut microbiota refers to the total of approximately 10 trillion diverse symbiotic organisms (including 50 bacterial phyla and about 100–1,000 bacterial species) within the human gut (an area of approximately 200–300 m^2^ of mucous membrane) (4). Recent studies have shown that the gut microbiota plays a pivotal role in many central nervous system (CNS) diseases, including ICH. For instance, gut microbiota depletion accelerates hematoma resolution and neurological recovery after ICH (5). Gut microbiota-derived phenylacetylglutamine mitigates neuroinflammation induced by ICH in mice (6). The gut-brain axis reveals the interaction between the brain and the gut microbiota (7). The gut microbiota can influence brain function and the response to injury, along this axis (8, 9). Changes in the gut microbiota has also been demonstrated to be associated with the response to acute CNS injury (10–12). For example, Celorrio et al. have found that the gut microbial dysbiosis induced by antibiotic increased the neuronal loss and reduces neurogenesis following traumatic brain injury, indicating that regulating the gut microbiota has therapeutic potential for brain injury (13). The development of abnormal gut microbiota-immune-brain axis is associated with brain injury in premature infants, and Klebsiella overgrowth shows predictive value for brain injury (14). Additionally, in animal models of intracerebral hemorrhage, gut microbiota dysbiosis and gastrointestinal dysfunction were observed, and the transplantation of healthy microbiota has been proven to promote neural function recovery by regulating the immune response (15). Therefore, exploring the gut microbiota-related biomarkers in ICH may provide novel insights into the underlying physiological mechanisms.

In this study, we investigated the gut microbiota-related differentially expressed genes (DEGs) in ICH based on bioinformatics analysis. The hub genes were selected using machine learning methods, and validated in clinical samples. We also established an ICH mouse model to investigate the effects of gut microbiota transplantation on ICH and the roles of host genes functionally associated with gut microbiota, which might provide clues for targeting the brain-gut axis in ICH therapy. The novelty and significance of this study lie in the integration of transcriptome data with clinical and animal validation results.

## Materials and methods

### Raw data acquisition and processing

An expression dataset from ICH patient (GSE24265 dataset) was downloaded from the Gene Expression Omnibus (GEO) database. This dataset included 11 human brain samples, among which four samples were from the surrounding areas of hematoma, and seven samples were from the gray and white matter regions on the opposite side. This dataset was generated by the GPL570 platform as a gene expression array. The gut microbiota-related genes (GMRGs) were obtained from the GeneCards database, with the condition of score>1 (16).

### Identification of GMRDEGs

After log2 transformation, the differentially expressed genes (DEGs) between perihematomal area and contralateral area were screened out using the limma R package. The criteria were |log Fold change (FC)| > 1 and *p*< 0.05. Common genes among DEGs and GMRGs were defined as gut microbiota-related differentially genes (GMRDEGs), and were identified and visualized using the VennDiagram R package.

### GO and KEGG enrichment analyses

Gene Ontology (GO) and Kyoto Encyclopedia of Genes and Genomes (KEGG) functional enrichment analyses were performed for DEGs and GMRDEGs using clusterProfiler R package under the condition of *p*< 0.05, *q*<0.05.

### Gene set enrichment analysis

GSEA was used to explore the enrichment pathways of the DEGs and GMRDEGs. The significantly enriched signals were selected and visualized using the clusterProfiler and msigdbr R packages, and visualized using the ggplot2 R package. The cutoff values were set as *p* < 0.05 and *q* < 0.05.

### Protein–protein interaction network construction

The STRING database was used to analyze GMRDEGs.^1^ The threshold was set as 0.4. The network information was input into Cytoscape software (version 3.9.1) for further analysis. The key nodes in the network were explored by analyzing the degree of nodes.

### Characteristic genes

Three machine learning algorithms (Lasso, RandomForest, svm-rfe) were adopted to select the characteristic genes from GMRDEGs. Lasso regression was conducted using the glmnet R package with the parameter *λ* to adjust the complexity. RandomForest is a method of integrating numerous decision trees into a forest, which is used for predicting the final outcomes. Its operation uses the RandomForest R package. The top 20 genes were selected based on their importance ranking. SVM-RFE is a supervised algorithm that ranks different features based on the differences in predictive ability. This algorithm is implemented using the e1071 R package.

### Immune infiltration analysis

The Cell-type Identification By Estimating Relative Subsets Of RNA Transcripts (CIBERSORT) method was applied to estimate the abundance of different types of immune cells based on the gene expression matrix. The CIBERSORT package and LM22 were used to evaluate the infiltration degree of different immune cells in control and ICH groups using the “Cibersort” R package. The permutation number was 100, and the LM22 signature matrix (22 immune cell subsets) was applied *p* < 0.05. The results were visualized in the form of bar charts to compare the infiltration degree of different types of immune cells in the control group and ICH samples. The corrplot R software package was used to explore the relationship between the level of characteristic genes and the proportion of immune cells.

### Clinical samples

A total of 5 mL fasting venous blood was collected from each ICH patient (*n* = 20) and from age-and gender matched healthy donor (*n* = 20). The inclusion criteria for ICH patients were as follows: (1) The onset of ICH occurred within 24 h. (2) The first spontaneous cerebral hemorrhage. (3) The patient and their family had signed an informed consent form. The exclusion criteria were as follows: (1) Severe dysfunction of vital organs such as the lung, heart, liver, and kidney. (2) History of craniocerebral surgery or massive hemorrhage of gastrointestinal tract. (3) Complicated with hematological system diseases, immune diseases, or malignant tumors.

### Animal model

The experimental procedure of this mouse model was carried out with the approval of the ethics committee of our hospital. Male C57BL/6 mice (8 weeks old, 25 ± 2 g) were provided by the Beijing Vital River Laboratory Animal Technology Co., Ltd. (Beijing, China) and housed under constant temperature and humidity conditions. After the mice adapted to the environment for 1 week, they were randomly divided into the Control, ICH and ICH + fecal microbiota transplantation (FMT) groups (*n* = 6 per group). The establishment method of the ICH mouse model was as described previously. In brief, the mice were anesthetized by intraperitoneal injection of 40 mg/kg 1% pentobarbital sodium. Then, the mice were fixed using a precise positioning device, and collagenase (0.05 U type VII collagenase in 0.5 μL saline, Sigma-Aldrich, United States) was injected stereoscopically into their brains to induce ICH. Finally, bone wax was applied to seal the drill holes, and the skin incisions were sutured. The mice in the control group received similar treatment but were not injected with collagenase. When the Longa score was ≥ 1, it was considered that the ICH model was successfully established.

The FMT treatment was performed as previously described (17). Briefly, fecal samples were collected from healthy donor mice. The samples were diluted in phosphate buffer saline (FBS) at 120 mg feces/1 mL PBS, and centrifuged. Then, the supernatant was obtained and re-centrifuged. After removing the supernatant, the samples were resuspended. For animals in the ICH + FMT group, the gut microbiota was removed by pretreating with streptomycin sulfate sterile water (500 mg/mL) for 5 days before the surgery. Then, the mice after the surgery received the prepared fecal suspension (50 μL) by gavage every day for 15 days.

### Modified neurological severity score evaluation

The mNSS score was applied for the assessment of neurological function (18). The score ranged from 0 to 18, indicating a progression from mild to severe injury, with the score increasing accordingly.

### Real-time reverse transcriptase-polymerase chain reaction

Total RNA was extracted from tissues using Trizol reagent. The reverse transcription of RNA was conducted using a High-Capacity cDNA reverse transcription kits (Thermo Fisher, United States). Then, a SYBR Green/ROX qPCR Master Mix (Thermo Fisher, United States) was used for real time PCR. The relative gene expression was calculated using the 2^−ΔΔCT^ method, and glyceraldehyde 3-phosphate dehydrogenase (GAPDH) was used as an internal control.

### Western blot

The radioimmunoprecipitation assay (RIPA) buffer (Thermo Fisher, United States) was used to isolate total protein from tissues. The concentration of protein samples was determined using a bicinchoninic acid (BCA) kit. Subsequently, protein samples (10 μg) were separated on 10% sodium dodecyl sulfate-polyacrylamide gel electrophoresis (SDS-PAGE) and then transferred to electro-transferred onto polyvinylidene fluoride (PVDF) membranes. After that, the membranes were incubated with 5% fat-free milk for 2 h and then incubated with primary antibodies including anti- ZO-1 (#ab276131, 1:1,000, Abcam), anti-Occludin (#ab216327, 1:1,000, Abcam), anti-Claudin-4 (#ab210796, 1:1,000, Abcam), anti-LEF1 (#ab137872, 1:1,000, Abcam, United Kingdom), anti-BLVRB (#sc-373692, 1:1,000, Santa Cruz Biotechnology, United States), anti-ITGAX (#PA5-90208, 1:1,000, Thermo Fisher, USA), anti-ATF4 (#PA5-27576, 1:1,000, Thermo Fisher, United States), and GAPDH (#ab9485, 1:2,500, Abcam, United Kingdom) at 4°C overnight. GAPDH served as the internal control. Next, the membranes were incubated with the Goat Anti-Rabbit IgG H&L (HRP) (#ab6721, 1:2,000, Abcam) for 60 min at room temperature. The interest proteins were observed using enhanced chemiluminescence and measured with ImageJ software.

### Statistical analysis

R (version 4.4.1) and Graphpad Prism 10 were used for data processing, analysis and visualization. Measurement data were expressed as mean ± standard deviation. Data comparisons between two groups were performed using t-tests. Data comparisons among multiple groups were conducted by one-way analysis of variance. Pearson correlation analysis was applied for exploring the correlation between levels of genes or infiltrating levels of immune cells. *p* < 0.05 was set as the threshold value.

## Results

### Exploration of DEGs in ICH

**Figure 1:**
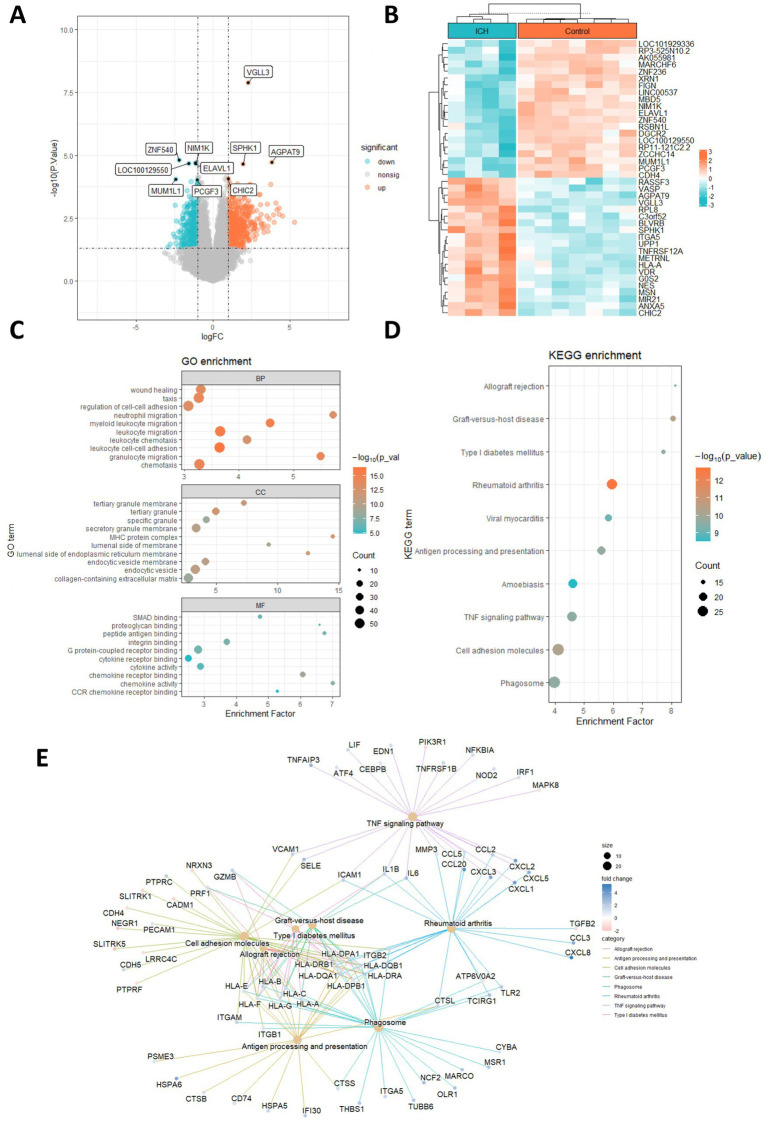
Identification of DEGs in ICH. **(A,B)** Volcano plot and heatmap showed the upregulated and downregulated DEGs in ICH. **(C,D)** GO and KEGG enrichment analysis of DEGs. **(E)** Network diagram showed the interactions of enriched pathways and related genes.

**Figure 2:**
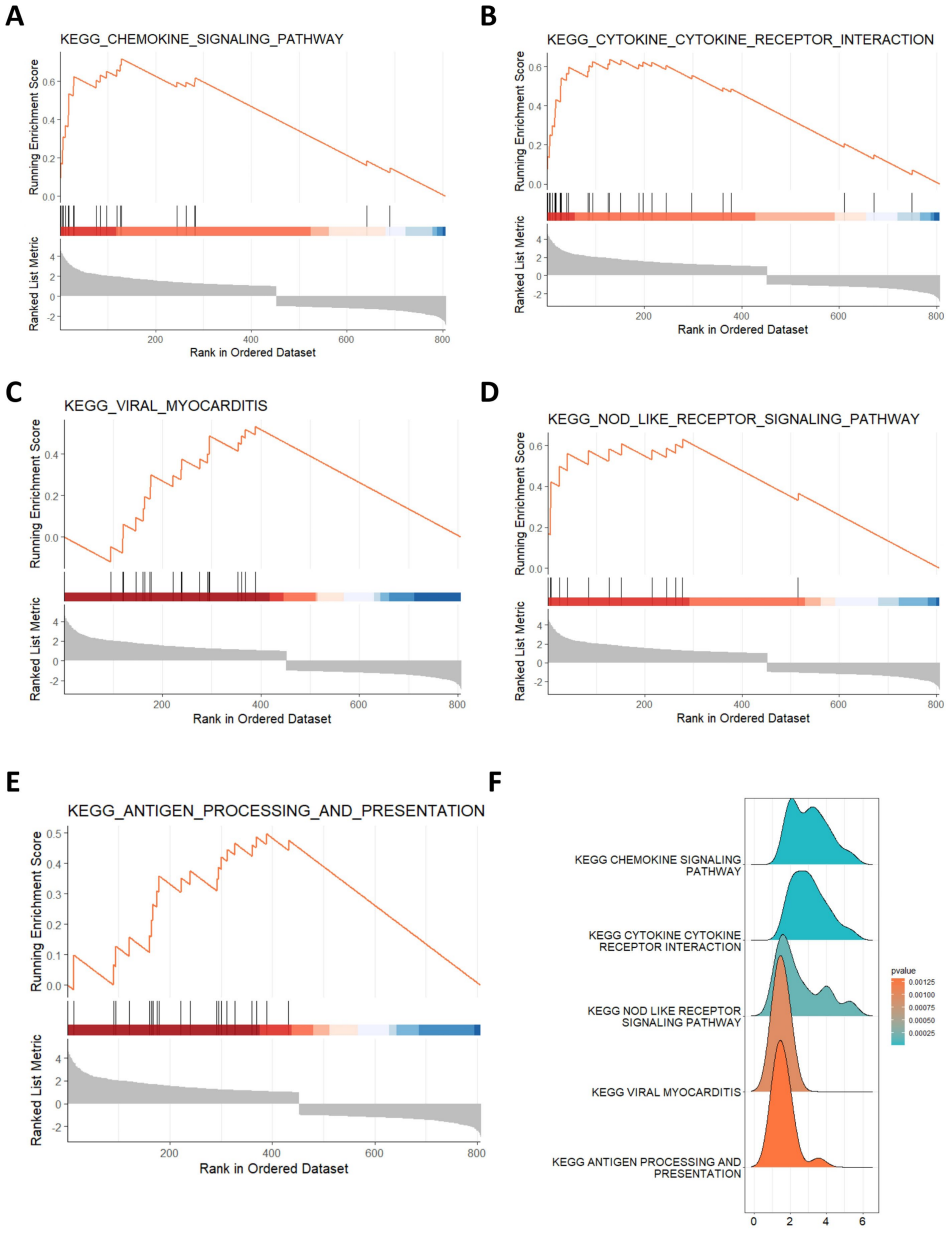
Gene set enrichment analysis of DEGs. **(A–F)** The top 5 enriched KEGG pathways for DEGs by GSEA.

The DEGs in human ICH were explored using the limma R package. As shown in Figure 1A, there were a total of 806 DEGs in ICH, among which 355 genes showed downregulation and 451 genes showed upregulation. The expression heatmap of the top 20 upregulated and downregulated DEGs was shown in Figure 1B. Moreover, we conducted the enrichment analysis of these DEGs in ICH. The GO enrichment analysis uncovered the significant enrichment of DEGs in biological process (BP) terms, such as leukocyte cell–cell adhesion, leukocyte migration, granulocyte migration, regulation of cell–cell adhesion, and wound healing. In terms of cell composition (CC), these DEGs were mainly related to major histocompatibility complex (MHC) protein complex, tertiary granule, endocytic vesicle and others. These DEGs were also associated with molecular function (MF) terms such as chemokine receptor binding, chemokine activity, integrin binding, peptide antigen binding, and SMAD binding (Figure 1C). Additionally, the results from KEGG analysis revealed that these DEGs were significantly associated with human immune diseases such as rheumatoid arthritis and graft-versus-host disease, allograft rejection, endocrine and metabolic disease such as Type I diabetes mellitus, and signaling pathway such as the TNF pathway (Figures 1D,E). Moreover, we conducted the GSEA on these DEGs. The results showed that these DEGs were enriched in KEGG pathways, such as chemokine signaling pathway, cytokine-cytokine receptor interaction, viral myocarditis, NOD-like receptor signaling pathway, and antigen-processing and presentation (Figures 2A–F).

### Identification of GMRDEGs

**Figure 3:**
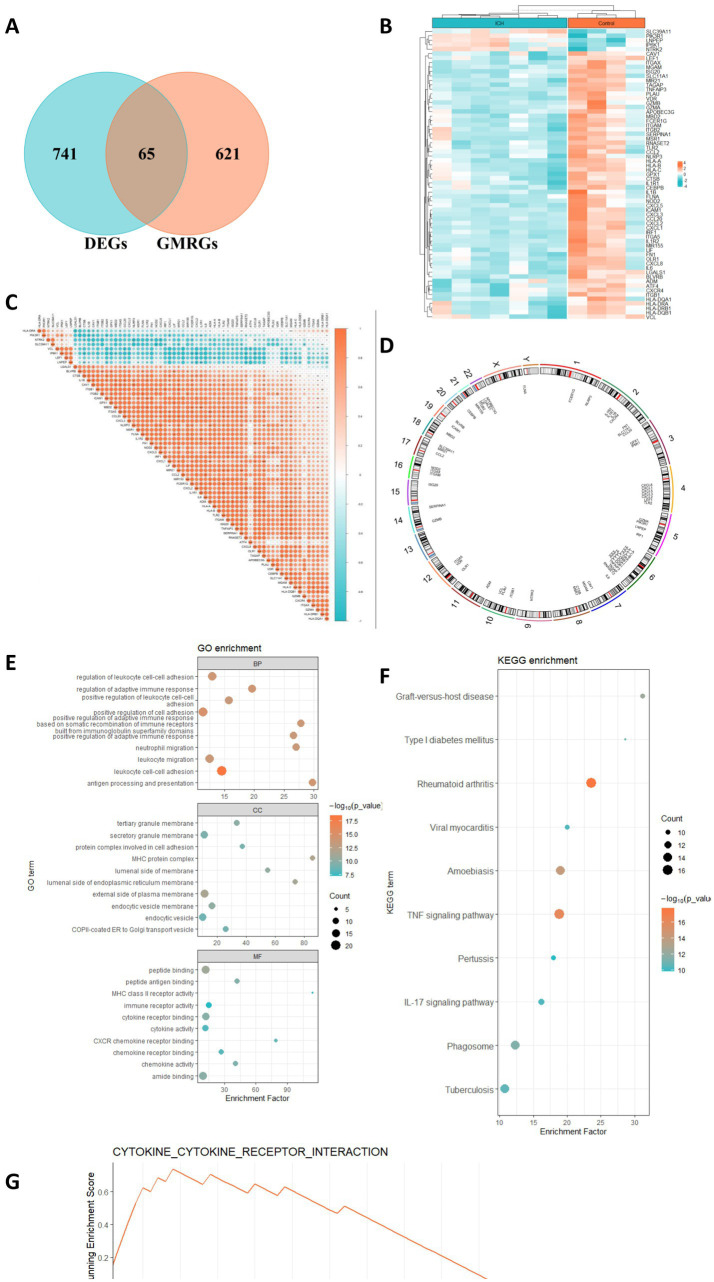
Identification of GMRDEGs in ICH. **(A)** Venn diagram of the DEGs in ICH and gut microbiota-related genes collected from genecards and previous literature. **(B,C)** The heatmap of 65 GMRDEGs in ICH and the expression correlation between the 65 GMRDEGs in ICH. **(D)** Chromosomal mapping of 65 GMRDEGs. **(E,F)** GO and KEGG enrichment analyses were performed for the 65 GMRDEGs. **(G)** GSEA of cytokine–cytokine receptor interaction. *p < 0.05, **p < 0.01, ***p < 0.001.

Then, we collected a total of 686 gut microbe-related genes from previous literature and the Genecards database. After intersecting the DEGs in ICH, we obtained 65 GMRDEGs (Figure 3A). A heatmap showed the expression pattern of these GMRDEGs, among which 5 genes were downregulated and 60 genes were upregulated (Figure 3B). We used Pearson correlation analysis to explore the expression correlations among the 65 GMRDEGs (Figure 3C). Additionally, we visualized the positions of these 65 GMRDEGs on the human chromosome using a circular graph, and found that the distribution of GMRDEGs on chromosome 6 was the most extensive (Figure 3D). Additionally, we subjected these GMRDEGs to functional enrichment analysis. GO enrichment analysis indicated that the GMRDEGs were associated with cell adhesion of leukocytes, regulation of the adaptive immune response, and leukocyte or neutrophil migration in terms of BP. The significantly enriched CC terms included MHC protein complex, external side of plasma membrane, and luminal side of endoplasmic reticulum membrane. The significantly enriched MF terms included peptide binding, amide binding, and cytokine receptor binding (Figure 3E). KEGG analyses demonstrated that these GMRDEGs were related to rheumatoid arthritis, tumor necrosis factor (TNF) signaling pathway, amoebiasis, and others (Figure 3F). GSEA was also conducted, and the results revealed that GMRDEGs were significantly related to the interaction between cytokine receptors (Figure 3G).

### PPI network of the GMRDEGs

**Figure 4:**
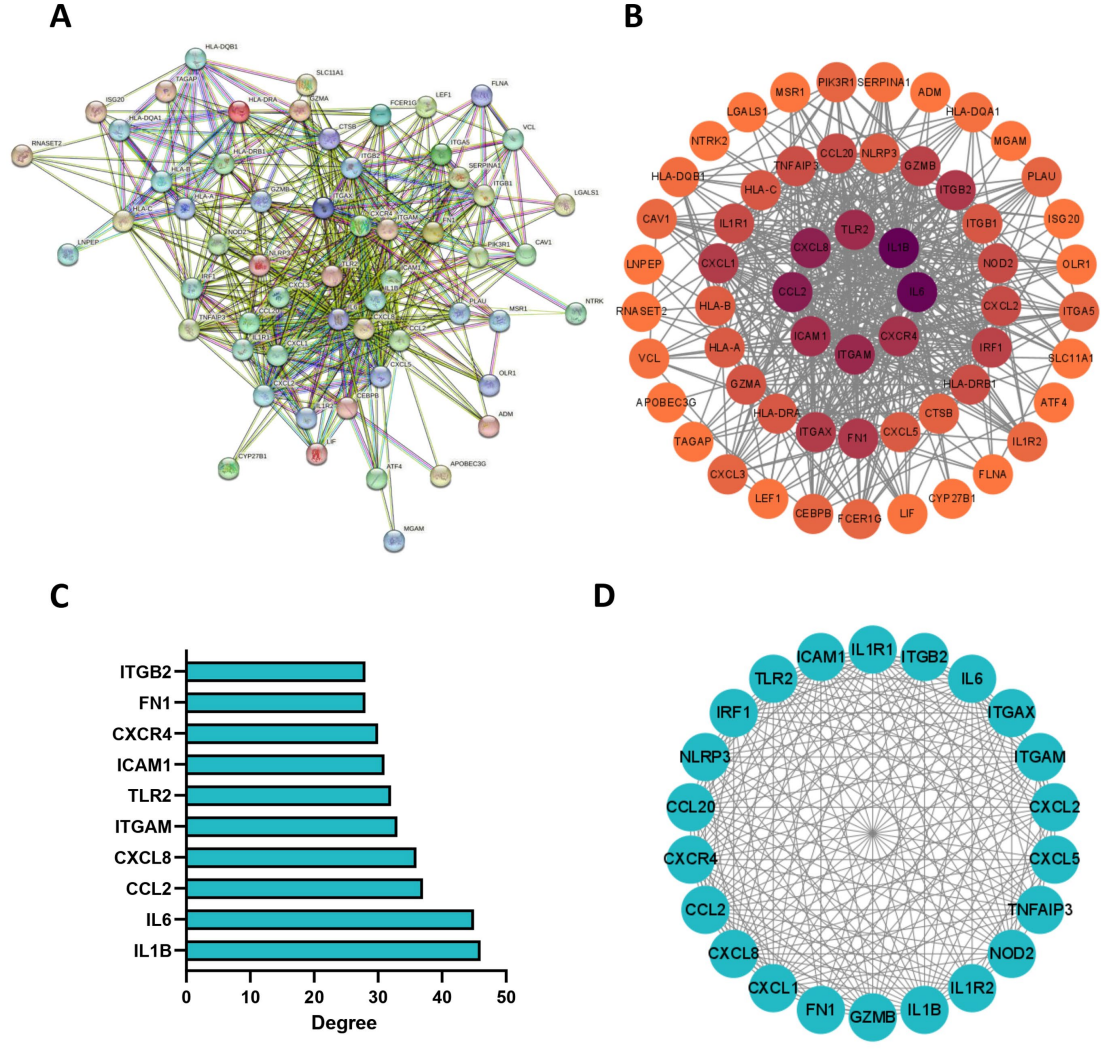
PPI network of GMRDEGs. **(A)** The PPI network of GMRDEGs was constructed using the STRING database under the minimum required interaction score of 0.4. **(B)** The Cytoscape was used to analyze the degree of nodes in the PPI network. The darker the color, the more important the node. **(C)** The barplot of top 10 GMRDEGs ranked by degree in the network. **(D)** The GMRDEGs screened by MCODE plug-in in the cluster 1.

Next, the PPI network of the GMRDEGs was constructed using the STRING database. Under the minimum required interaction score of 0.4, the network contained 58 connected nodes and 490 edges after hiding disconnected nodes (Figure 4A). The results were input into the Cytoscape software for further analysis. After analyzing the network, we obtained the degree indicating the importance of the nodes in the network (Figure 4B). The top 10 GMRDEGs ranked by degree were shown in Figure 4C, namely interleukin 1 B (IL1B), interleukin 6 (IL6), C-C chemokine ligand 2 (CCL2), C-X-C motif chemokine ligand 8 (CXCL8), integrin subunit alpha M (ITGAM), toll like receptor 2 (TLR2), intercellular adhesion molecule 1 (ICAM1), C-X-C motif chemokine receptor 4 (CXCR4), fibronectin 1 (FN1), and integrin subunit beta 2 (ITGB2). Additionally, we analyzed the network of GMRDEGs using the MCODE plug-in. The cluster 1 was the mostly densely connected cluster, with 22 nodes and 199 edges. The interaction network was shown in Figure 4D.

### Selection of characteristic genes

**Figure 5:**
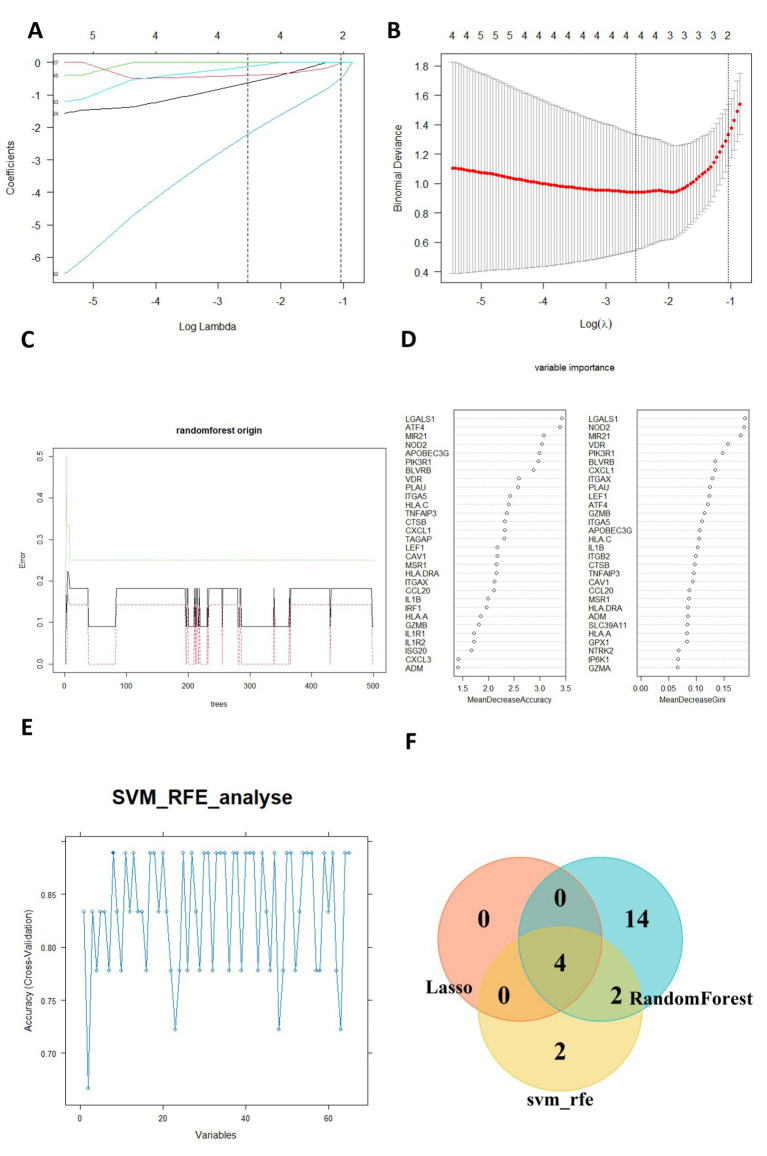
Selection of hub genes by machine learning algorithms. **(A)** Ten cross-validations of adjusted parameter selection in the LASSO model. **(B)** LASSO coefficient analysis. Vertical dashed lines are plottedat the best lambda. **(C)** Relationship between the number of randomforest trees and error rates. **(D)** The importance of GMRDEGs were ranked by MeanDecreaseaccuracy and MeanDecreaseGini. **(E)** SVM-RFE was applied for characteristic gene selection. **(F)** The venn diagram showed the common hub genes selected by the three algorithms.

The selection of characteristic genes was implemented based on machine-learning methods. Lasso regression analysis selected four feature genes from the 65 GMRDEGs (Figures 5A,B). Based on the random Forest algorithm, the top 20 GMRDEGs ranked by importance score were selected (Figures 5C,D). For the support vector machine-recursive feature elimination (SVM-RFE) algorithm, the highest accuracy was achieved as the feature number was eight, and eight characteristic genes were screened (Figure 5E). After intersecting the feature genes selected by the three machine learning algorithms, four common feature genes were obtained and regarded as hub genes: lymphoid enhancer-binding factor 1 (LEF1), integrin subunit alpha X (ITGAX), biliverdin IXbeta reductase (BLVRB), and activating transcription factor 4 (ATF4) (Figure 5F).

### Expression pattern of the hub genes

**Figure 6:**
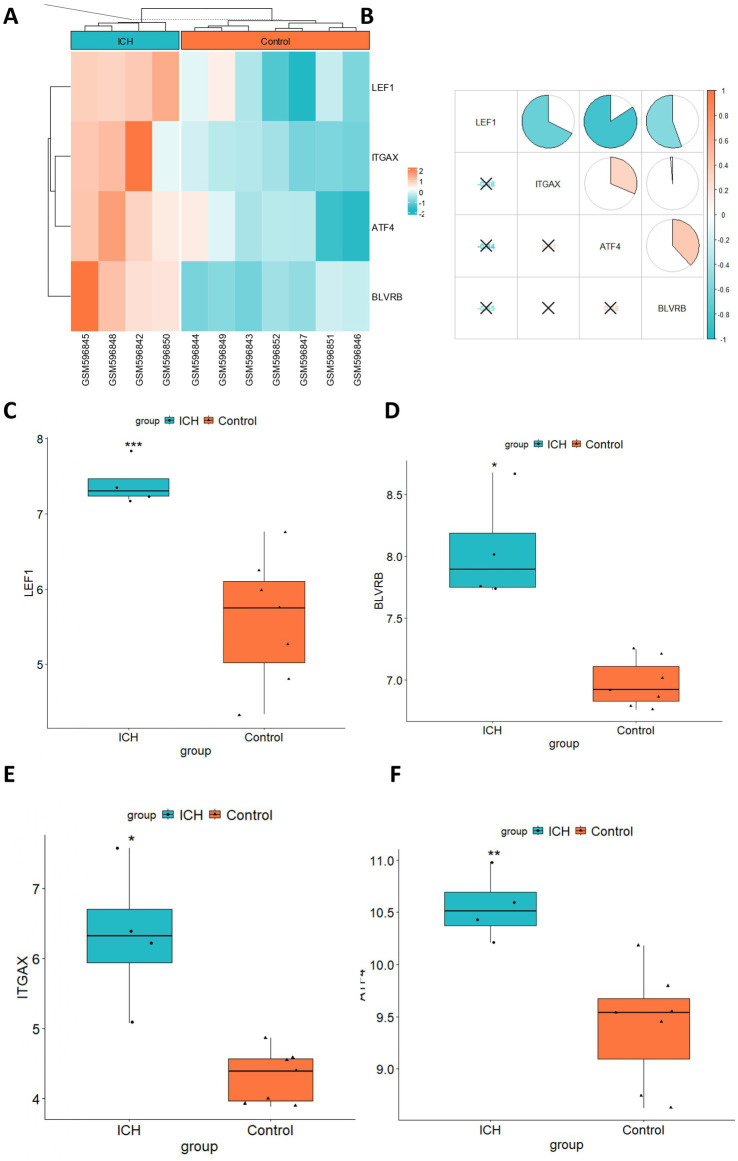
Expression pattern of hub genes in ICH. **(A)** The heatmap showed the expression profile of 4 hub genes in ICH. **(B)** The expression correlation between hub genes was evaluated by the Pearson correlation analysis. Box plots showed the expression comparison between the ICH and control group for **(C)** LEF1, **(D)** BLVRB, **(E)** ITGAX, and **(F)** ATF4. *p < 0.05, **p < 0.01, ***p < 0.001.

We further visualized the expression patterns of the four selected hub genes in ICH. The results showed that all four hub genes were significantly upregulated in the ICH group (Figure 6A). The correlation between hub gene expression levels was also explored. Notably, there was no significant correlation among the four hub genes (Figure 6B). Moreover, we visualized the expression of four hub genes in box plots, which showed significant upregulation of four genes (Figures 6C–F).

### Immune infiltration analysis

**Figure 7:**
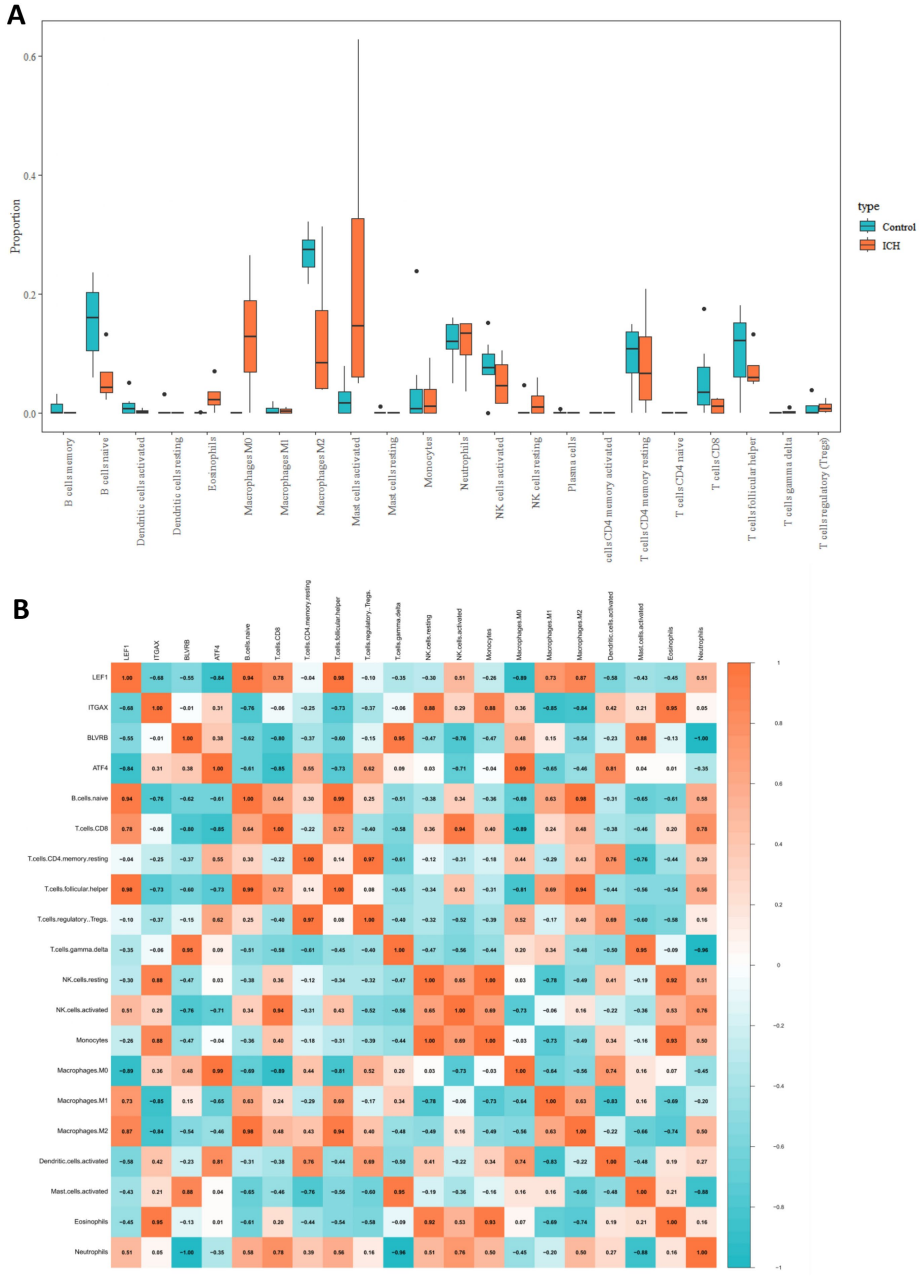
Immune infiltration analysis in ICH. **(A)** The comparison of proportion of immune cells between ICH and control groups. **(B)** The heatmap showed the correlation between immune cell infiltration levels and expression hub genes.

**Figure 8:**
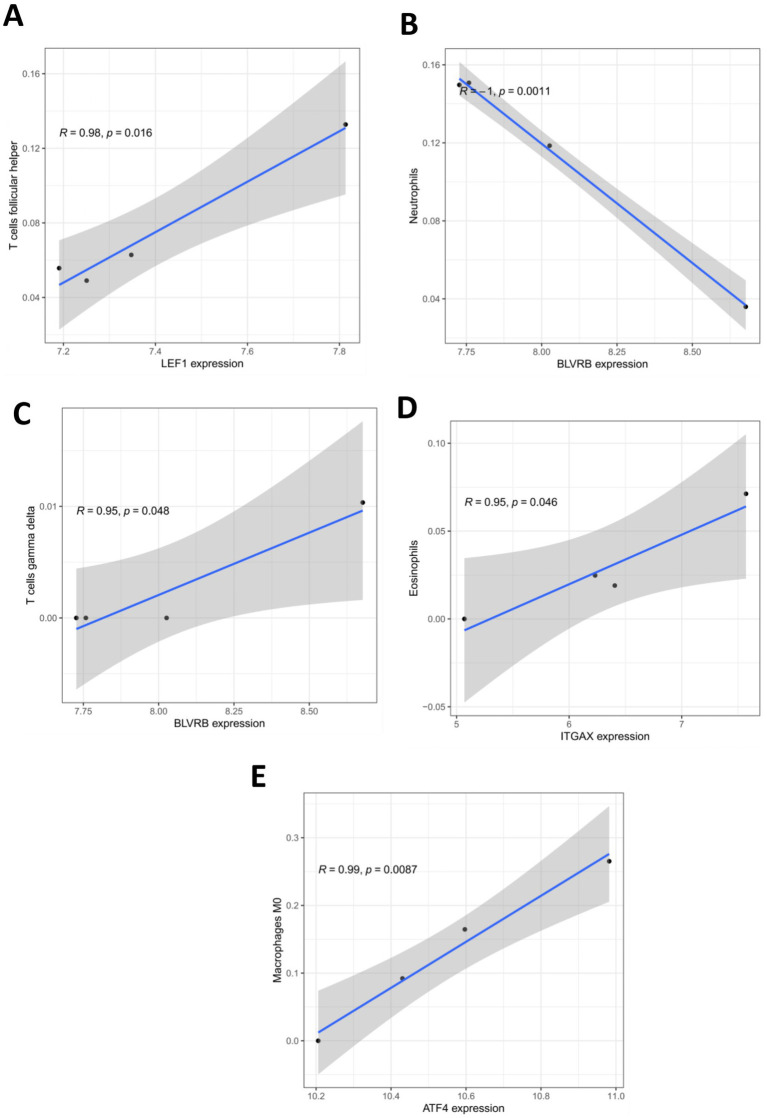
Correlation between hub gene expression and infiltration levels of immune cells. **(A)** The correlation between LEF1 expression and proportion of T cells follicular helper in ICH group. **(B)** The correlation between BLVRB expression and proportion of neutrophils in ICH group. **(C)** The correlation between BLVRB expression and proportion of T cells gamma delta in ICH group. **(D)** The correlation between ITGAX expression and proportion of Eosinophils in ICH group. **(E)** The correlation between ATF4 expression and proportion of macrophage M0 in ICH group.

Additionally, the proportions of different types of immune cells in the ICH and control groups were compared. The results demonstrated that the infiltrating levels of naïve B cells, M2 macrophages, activated NK cells, naïve CD4, CD8, and follicular helper T cells were lower in the perihematomal area than in the contralateral gray matter and white matter. The proportions of eosinophils, M0 macrophages, activated mast cells, and resting NK cells were higher in the perihematomal area (Figure 7A). The correlation between hub genes and the proportion of immune cells in the ICH group was analyzed and was shown as a heatmap (Figure 7B). LEF1 expression was positively correlated with the proportion of follicular helper T cells (*r* = 0.98, *p* = 0.016) (Figure 8A). BLVRB levels were negatively correlated with the abundance of neutrophils (*r* = −1, *p* = 0.0011) (Figure 8B) and were positively correlated with the abundance of gamma delta T cells (*r* = 0.95, *p* = 0.048) (Figure 8C). ITGAX expression was found to be positively correlated with the proportion of eosinophils (*r* = 0.95, *p* = 0.046) (Figure 8D). ATF4 expression was positively correlated with the proportion of M0 macrophages (*r* = 0.99, *p* = 0.0087) (Figure 8E).

### Validation of the expression of hub genes in ICH patients

**Figure 9:**
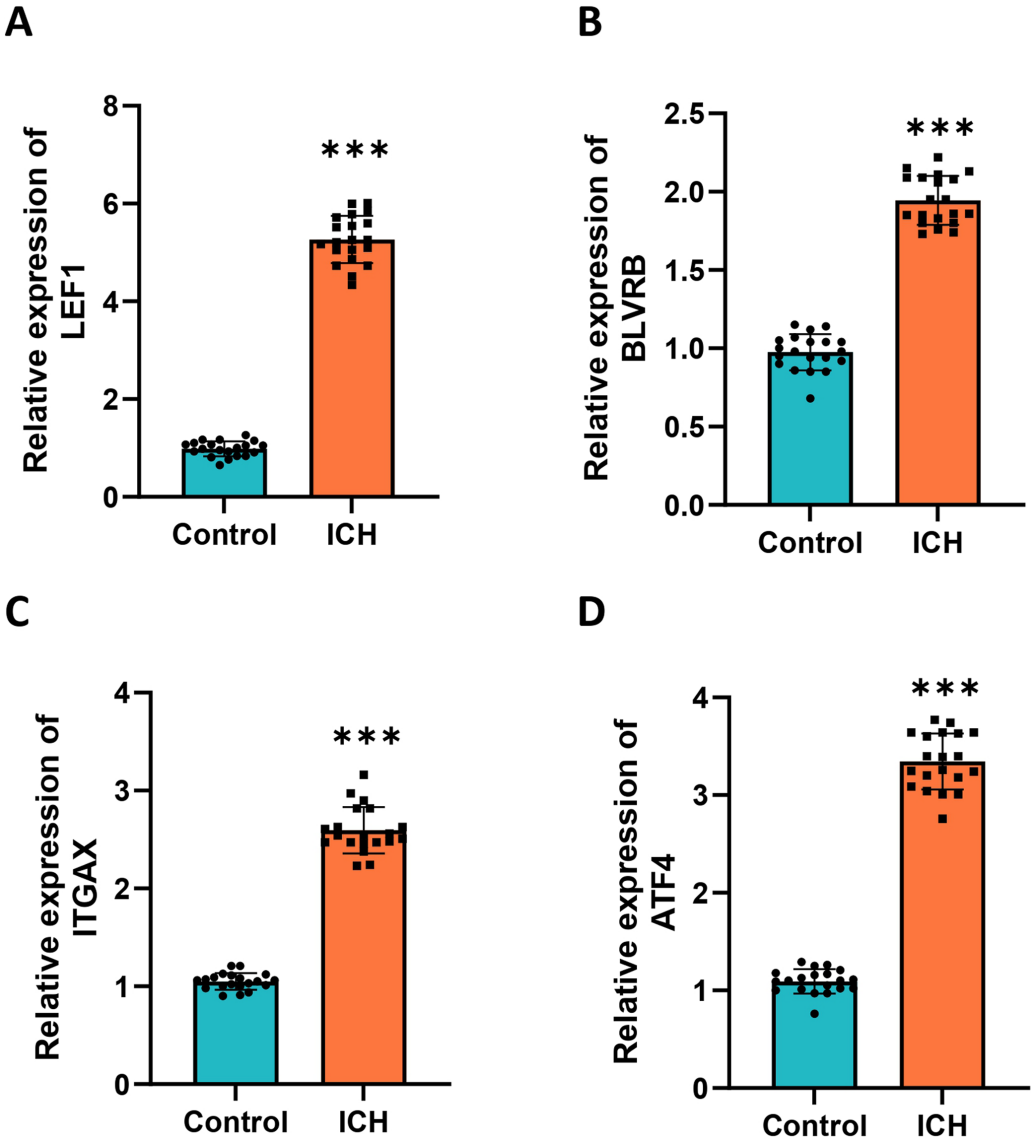
Validation of hub genes in clinical samples. RT-qPCR analysis was performed to detect the expression of **(A)** LEF1, **(B)** BLVRB, **(C)** ITGAX, and **(D)** ATF4 using RT-qPCR in the blood samples of ICH patients and healthy donors. Measurement data were expressed as mean ± standard deviation. Data comparisons between two groups were performed using *t*-tests.

Twenty ICH patients and healthy donors were enrolled in the study. Peripheral blood was collected, and RT-qPCR was performed to detect the expression of selected hub genes in ICH patients and healthy controls. The results revealed that all four hub genes (LEF1, BLVRB, ITGAX, and ATF4) were significantly upregulated in ICH patients compared to healthy donors (Figures 9A–D), and these findings were in line with the results of prior bioinformatics analyses.

### FMT alleviated ICH in mice possibly by regulating the expression of hub genes

**Figure 10:**
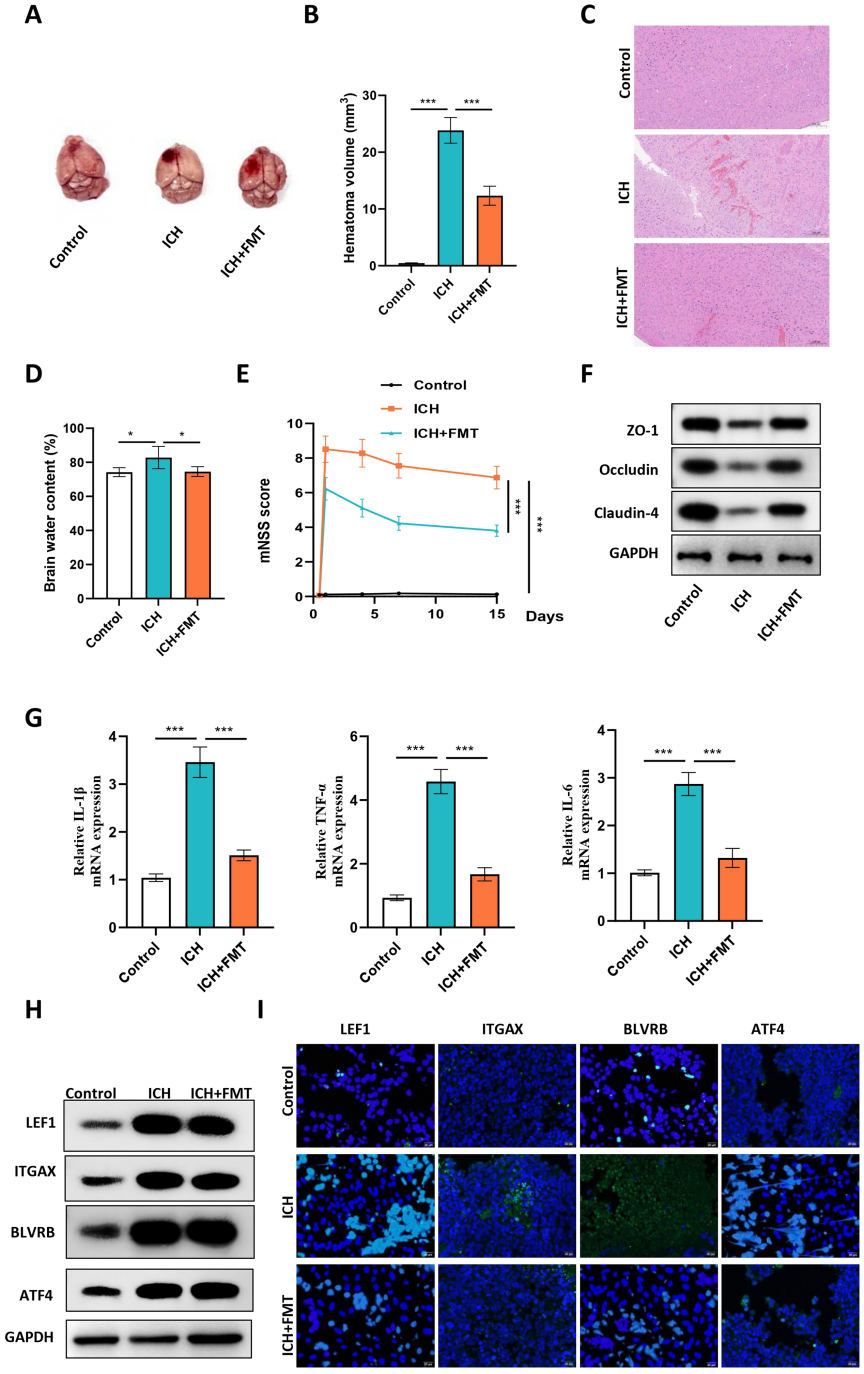
FMT alleviated ICH in mice and downregulated the expression of hub genes *in vivo*. **(A)** Representative images of mouse brains in each group. **(B)** The hematoma volume was quantified in each group. **(C)** Representative images of HE staining of brain sections of mice in the control, ICH and ICH + FMT groups. **(D)** Brain water content of mice in each group. **(E)** The mNSS score was used to assess the neurological dysfunction of mice in each group. **(F)** Western blot was conducted to detect the levels of proteins related to intestinal barrier destruction in the colon samples of mice in each group. **(G)** The mRNA expression of inflammatory factors in peripheral blood of mice in each group was detected using RT-qPCR. **(H)** Western blot was conducted to detect the proteins levels of hub genes in brain samples of mice in each group. **(I)** The representative images of IF staining in brain tissues of mice in each group. *N* = 6, data were presented as mean ± standard deviation. One-way or two-way analysis of variance was performed for statistical analysis.

Furthermore, we investigated the impact of FMT on ICH development. A collagenase-induced mouse model of ICH was established, and we found that the hematoma volume in ICH mice was reduced by FMT treatment (Figures 10A–C). We also demonstrated that FMT treatment significantly reversed ICH-induced elevation of brain water content (Figure 10D). Moreover, the mNSS score increased by ICH was partially restored by FMT, suggesting that FMT alleviated neurological dysfunction in ICH mice (Figure 10E). The levels of proteins related to intestinal barrier destruction in the colon samples were detected. The results revealed that FMT alleviated the ICH-induced downregulation of the tight junction proteins ZO-1, Occludin, and Claudin-4 (Figure 10F). Furthermore, we found that the levels of inflammatory factors were elevated in the blood samples of ICH mice and showed a significant reduction in response to FMT treatment, indicating that FMT attenuated the inflammatory response following ICH (Figure 10G). Additionally, we detected the levels of four hub genes in ICH and the impact of FMT on hub gene expression. The results uncovered that all four hub genes were upregulated in brain samples of ICH mice and were significantly downregulated by FMT treatment (Figures 10H,I).

## Discussion

ICH is responsible for 10–15% of all stroke cases and is characterized by a high death rate and adverse prognosis (19). Exploring the biomarkers of ICH might help develop a non-invasive method for ICH diagnosis and prognosis evaluation. N-terminal pro B-type natriuretic peptide (NT-proBNP) is increased in the early phase after ICH, and its combination with glial fibrillary acidic protein (GFAP) is useful for differentiating stroke subtypes in the hyperacute phase (20). Matrix metalloproteinase-9 (MMP-9) is regarded to predict the risk of hemorrhagic transformation after recombinant tissue plasminogen activator (rt-PA), and the serum level of MMP-9 is associated with the blood brain barrier disruption (21–23). However, the diagnostic accuracy and specificity of these biomarkers require further clinical validation. Understanding the mechanisms underlying ICH is necessary for effective diagnosis and therapy.

Based on bioinformatic analysis, we screened the DEGs in ICH. We screened 806 DEGs, among which 451 were upregulated and 355 were downregulated in perihematomal tissue compared with the contralateral grey matter and white matter in ICH patients. Enrichment analyses illustrated the close association of these DEGs with biological processes, such as leukocyte cell–cell adhesion, leukocyte migration, granulocyte migration, and wound healing. The enriched cellular component terms included MHC protein complex, tertiary granule, endocytic vesicle, and molecular function terms such as chemokine receptor binding, integrin binding, and peptide antigen binding. These results indicate primary insult after blood–brain barrier (BBB) dysfunction, with disrupted tight junctions (TJs), transporters, transcytosis, and aberrant expression of leukocyte adhesion molecules, causing brain edema and immune infiltration (24, 25). The DEGs were also related to signaling pathways such as the TNF signaling pathway that contributes to neuroinflammatory injury in ICH (26–28), and are associated with diseases such as Type I diabetes mellitus, which is closely linked with the increased risk of cardiovascular events, including stroke (29). Moreover, we screened 65 gut microbe-related DEGs after intersecting the DEGs with GMRGs, and the results from enrichment analyses revealed that the GMRDEGs were also related to the immune response regulation and migration or adhesion of immune cells, and were enriched in signaling pathways such as TNF and IL-17 signaling pathways. Additionally, we constructed a PPI network for the GMRDEGs, and the results revealed that GMRDEGs, such as IL1B, IL6, and CCL2, were of high importance in the network.

Furthermore, we used machine learning algorithms and screened four gut microbe-related hub genes for ICH, including LEF1, ITGAX, BLVRB, and ATF4. LEF1 is a core transcription factor of the Wnt/*β*-catenin pathway. It forms a complex with β-catenin and regulate the expression of downstream genes (such as MYC, CYCLIN D1), promoting cell proliferation and differentiation (30). Emerging studies have revealed the role of LEF1 in the development of human cancers, including prostate, colon, and lung cancers (31–33). Previous studies have also revealed its role in IS. For example, Zhang et al. found that the level of LEF1 increased following IS at both the mRNA and protein levels (34). A bioinformatics analysis study also has indicated that LEF1 is related to the molecular subtypes and immune regulation of IS (35). ITGAX is a dendritic cell marker that is expressed in microglial cells in the CNS, and is implicated in phagocytosis (36, 37). Significant enrichment of microglia and elevation of ITGAX are observed after IS, which indicates a unique microglial cell characteristic related to neurodegenerative diseases that emerged after IS (38). BLVRB is a member of the biliverdin reductase family and is involved in the conversion of biliverdin to bilirubin. Bilirubin has strong antioxidant properties and can eliminate free radicals, thereby reducing oxidative stress damage (39). A previous study has demonstrated that plasma BLVRB levels are associated with intraplaque hemorrhage and an increased risk of recurrent IS in patients with low- to moderate-grade carotid stenosis (40). ATF4 is a member of the ATF/cAMP response element-binding protein (CREB) family and is implicated in physiological processes, such as endoplasmic reticulum (ER) stress, inflammation, and oxidative stress (41). As reported in previous studies, ATF4 levels are upregulated after cerebral ischemia and reperfusion in rats (42, 43). In addition, ATF4 plays a crucial role in ICH. It regulates neuronal apoptosis through the interaction between the endoplasmic reticulum stress PERK/ATF4 pathway and mitochondrial one-carbon metabolism, thereby influencing the neurological damage and functional recovery after ICH (44). In this study, we found that LEF1, ITGAX, BLVRB, and ATF4 were upregulated in ICH, and were validated to be highly expressed in the plasma of ICH patients compared with the healthy controls, suggesting the value of these four biomarkers in ICH diagnosis. However, our research did not sufficiently focus on the expression differences of LEF1, ITGAX, BLVRB, and ATF4 in different tissues. Different tissues have unique microenvironments and physiological functions, and the expression patterns and regulatory mechanisms of core genes in different tissues may show significant differences. For instance, brain tissue exhibits high neuronal specificity and a complex neural network structure, while blood constitutes a dynamic circulatory system that exchanges substances and transmits signals with various tissues throughout the body. Therefore, the expression differences of LEF1, ITGAX, BLVRB, and ATF4 in the brain and blood may reflect their distinct roles in different physiological and pathological processes. In our subsequent research, we plan to collect more types of brain and blood samples, and employ high-throughput sequencing technology to comprehensively analyze the expression profiles of LEF1, ITGAX, BLVRB, and ATF4 in different tissues.

Immune infiltration by major immune cells can be observed in human perihematomal tissue on day 1 post-ICH (45, 46). The immune response to ICH is also closely related to inflammation and patient recovery (25). This study analyzed the pattern of immune cell infiltration in perihematomal tissue and the corresponding contralateral white and gray matter. We found increased infiltration of eosinophils, macrophage M0, activated mast cells, and resting NK cells, and a decreased proportion of B cells, M2 macrophages, activated NK cells, and T cells, suggesting a pro-inflammatory and dysregulated immune microenvironment. Additionally, we explored and identified the relationship between the levels of characteristic genes and infiltrating levels of immune cells, suggesting a potential relationship between hub genes and immune response in ICH. However, we did not deeply explore the underlying molecular mechanism and functional consequences. In the future, we will conduct in-depth research using cell co-culture experiments, gene editing techniques, and animal models to study how core genes affect the recruitment, activation, and differentiation of immune cells, as well as how these changes further affect the inflammatory response, blood–brain barrier permeability, and neuronal damage in the pathological process of intracranial hemorrhage, thereby establishing a clear causal relationship chain of hub genes-immune infiltration-disease progression.

Increasing evidence has demonstrated a vital role of the brain-gut axis in ICH (47, 48). The gut microbiota participates in different phases of stroke and can act as a risk and contributing factor. ICH can induce a neuroinflammatory response and alter peripheral immunity, leading to gut microbiota dysbiosis and increased intestinal permeability (15). The transplantation of healthy gut microbiota has been shown to improve recovery after ICH by improving functional deficits and modulating neuroinflammation (49). Our work revealed that FMT decreased the hematoma volume of ICH mice, improve neurological function, and reduced intestinal barrier permeability in ICH mice. Moreover, we found that FMT could also reverse the ICH-induced upregulation of LEF1, BLVRB, ITGAX, and ATF4, suggesting their involvement in the brain-gut axis in ICH. At present, our understanding of the mechanism of FMT in treating intracranial hemorrhage is still relatively limited, especially regarding the interaction mechanisms between inflammatory signaling pathways and the microbiota as well as the host genes. In subsequent studies, we will employ multi-omics technologies such as metagenomics, transcriptomics, and metabolomics to comprehensively analyze the changes in the intestinal microbiota after FMT treatment, the expression regulation of host genes, and the alterations in metabolites. We will also combine cell experiments and animal models to verify the correlations between these changes and biological processes such as inflammatory responses, immune regulation, and neuroprotection, thereby deeply revealing the potential molecular mechanisms of FMT in treating ICH.

### Strengths and limitations

The strength of this study was that we identified gut microbiota-related biomarkers in cerebral hemorrhagic stroke using bioinformatic analysis and experimental validation. Four hub genes, LEF1, ITGAX, BLVRB, and ATF4, were significantly upregulated in ICH and associated with immune cell infiltration. Their expression was validated in patient plasma samples and in a collagenase-induced ICH mouse model. Notably, FMT alleviated ICH severity and reversed the dysregulation of these hub genes, suggesting their involvement in the brain-gut axis. These findings provide new insights into the molecular mechanisms underlying ICH and highlight potential therapeutic targets for microbiota-based interventions. Our study had some limitations. First, the analyzed public dataset (GSE24265) originated from brain tissue, whereas subsequent validations involved blood samples and animal brain tissues. The cellular composition of the brain tissue and blood is different (for example, neurons vs. immune cells), which may lead to inconsistent gene expression patterns. In addition, the brain structure of animals differs from that of humans, which may lead to incomplete consistency in gene function. Therefore, in future studies, non-human primate animal models, single-cell sequencing technology, clinical validation and multi-omics approaches. Should be incorporated to further optimize the cross-tissue validation framework. Second, the explicit role and mechanism of these biomarkers in the pathological process ICH remain unclear and require further exploration in the future. Therefore, further research will be conducted to explore how the gut microbiota specifically regulates the expression of the four key genes, LEF1, BLVRB, ITGAX and ATF4 in ICH.

## Conclusion

This study screened four gut microbiota-related hub genes in ICH: LEF1, BLVRB, ITGAX, and ATF4. These four genes were upregulated in perihematomal tissue and were associated with infiltrating levels of immune cells. Further studies validated their upregulation in plasma obtained from ICH patients and in a mouse model of ICH. Additionally, we revealed that FMT treatment alleviated ICH potentially by downregulating the expression of these hub genes. Therefore, LEF1, BLVRB, ITGAX and ATF4 are expected to become new biomarkers for intracranial hemorrhage. By detecting the expression levels of these genes in patients’ blood, doctors can diagnose intracranial hemorrhage more quickly and accurately, especially when the early symptoms of the disease are not typical. This can provide valuable time for timely intervention and treatment. At the same time, changes in the expression levels of these genes may be closely related to the severity and prognosis of intracranial hemorrhage. Further research can establish a prognostic assessment model based on these genes to assist doctors in formulating individualized treatment plans.

## Data Availability

The datasets presented in this study can be found in online repositories. The names of the repository/repositories and accession number(s) can be found in the article/Supplementary material.

## Footnotes

^1^ http://string-db.org/
