# Supplementary figures and images for: Identification of novel gut microbiota-related biomarkers in cerebral hemorrhagic stroke
Source: Front Med (Lausanne). 2025 Aug 26;12:1636860. doi: 10.3389/fmed.2025.1636860 (PMC12417508; doi:10.3389/fmed.2025.1636860)

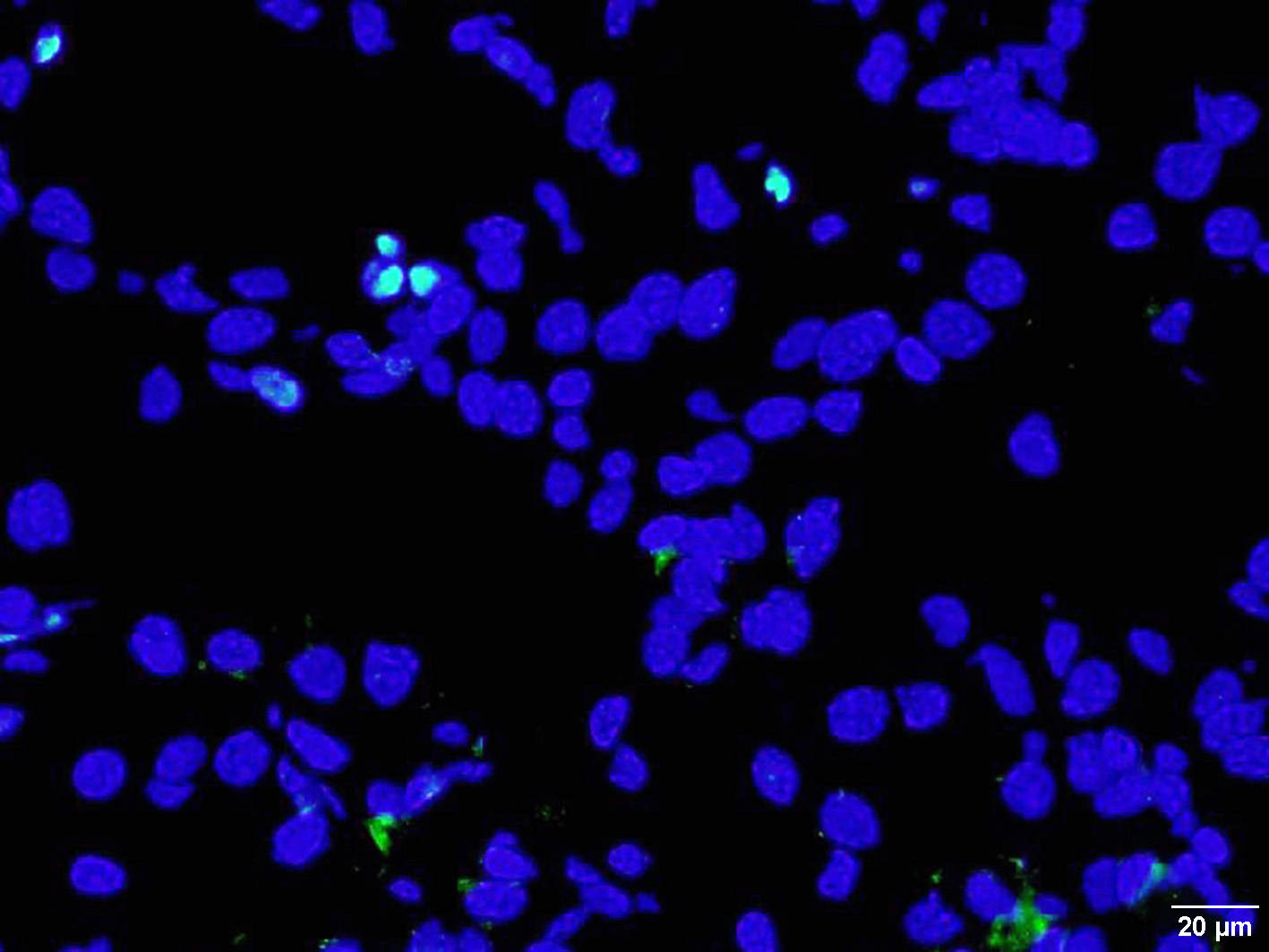

Supplement: Supplementary file 1 [file Data_Sheet_1.ZIP › Original IF images/Control magnification × 100/LEF1/Merge.jpg]

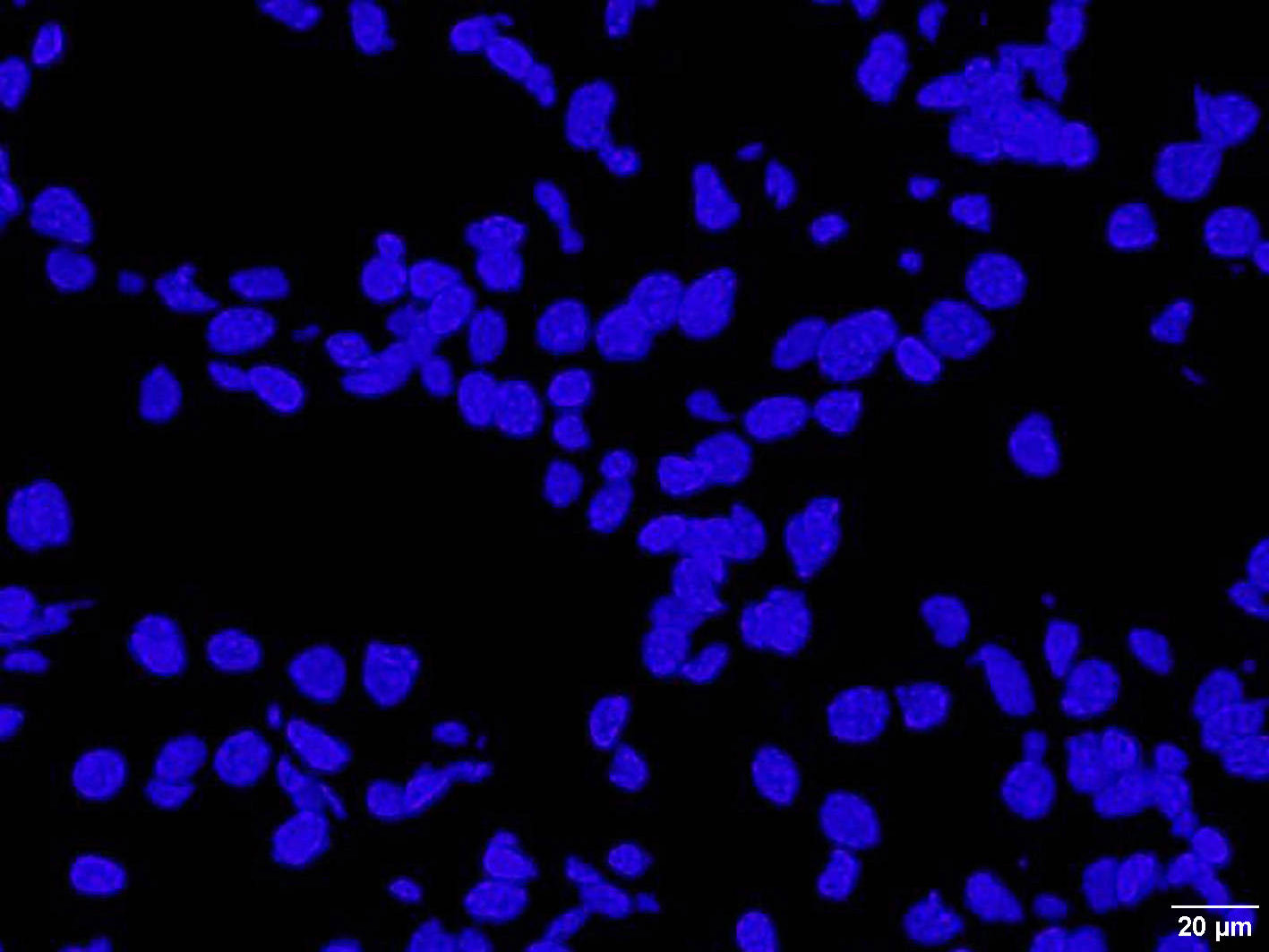

Supplement: Supplementary file 1 [file Data_Sheet_1.ZIP › Original IF images/Control magnification × 100/LEF1/DAPI.jpg]

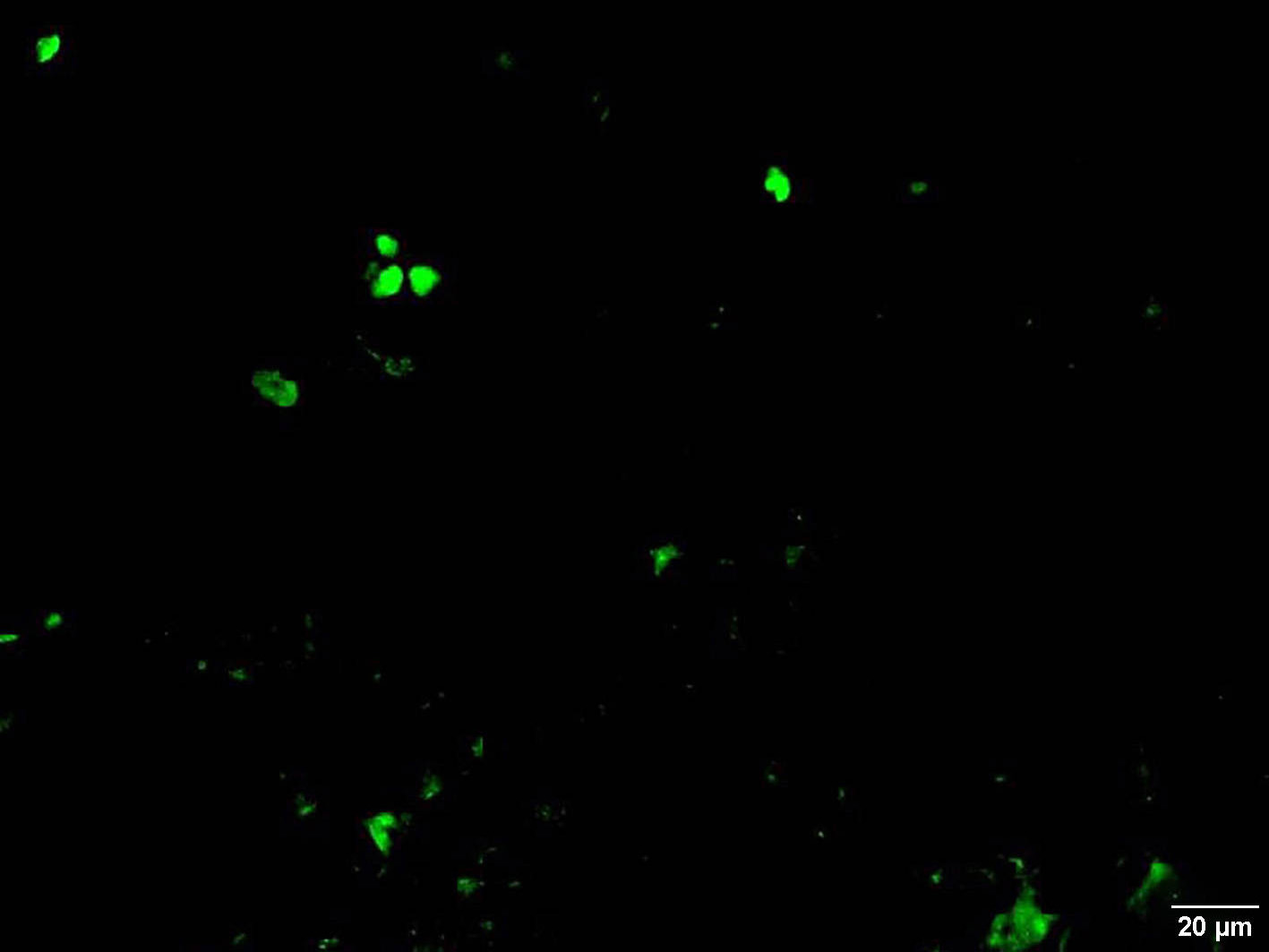

Supplement: Supplementary file 1 [file Data_Sheet_1.ZIP › Original IF images/Control magnification × 100/LEF1/LEF1.jpg]

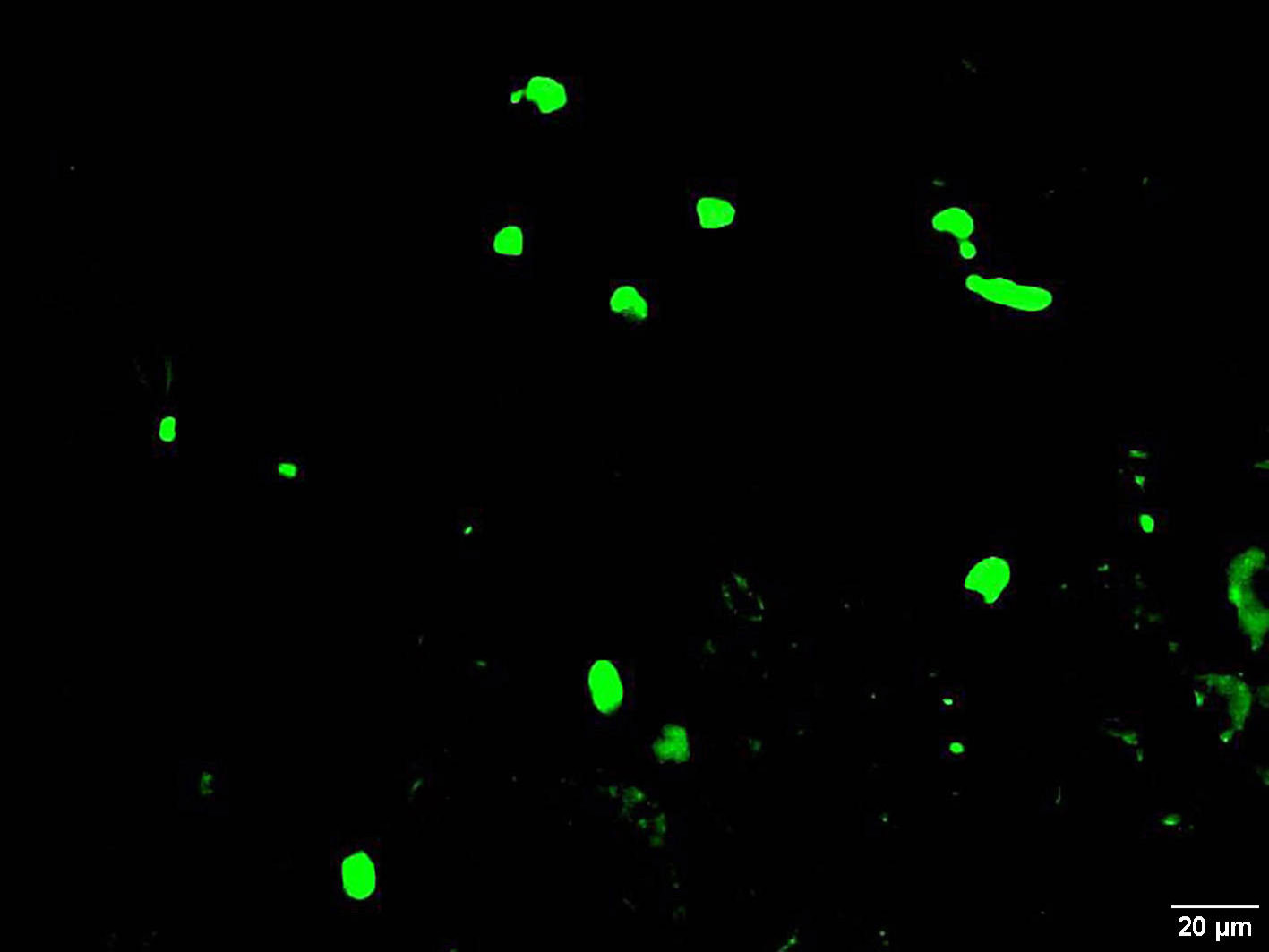

Supplement: Supplementary file 1 [file Data_Sheet_1.ZIP › Original IF images/Control magnification × 100/BLVRB/BLVRB.jpg]

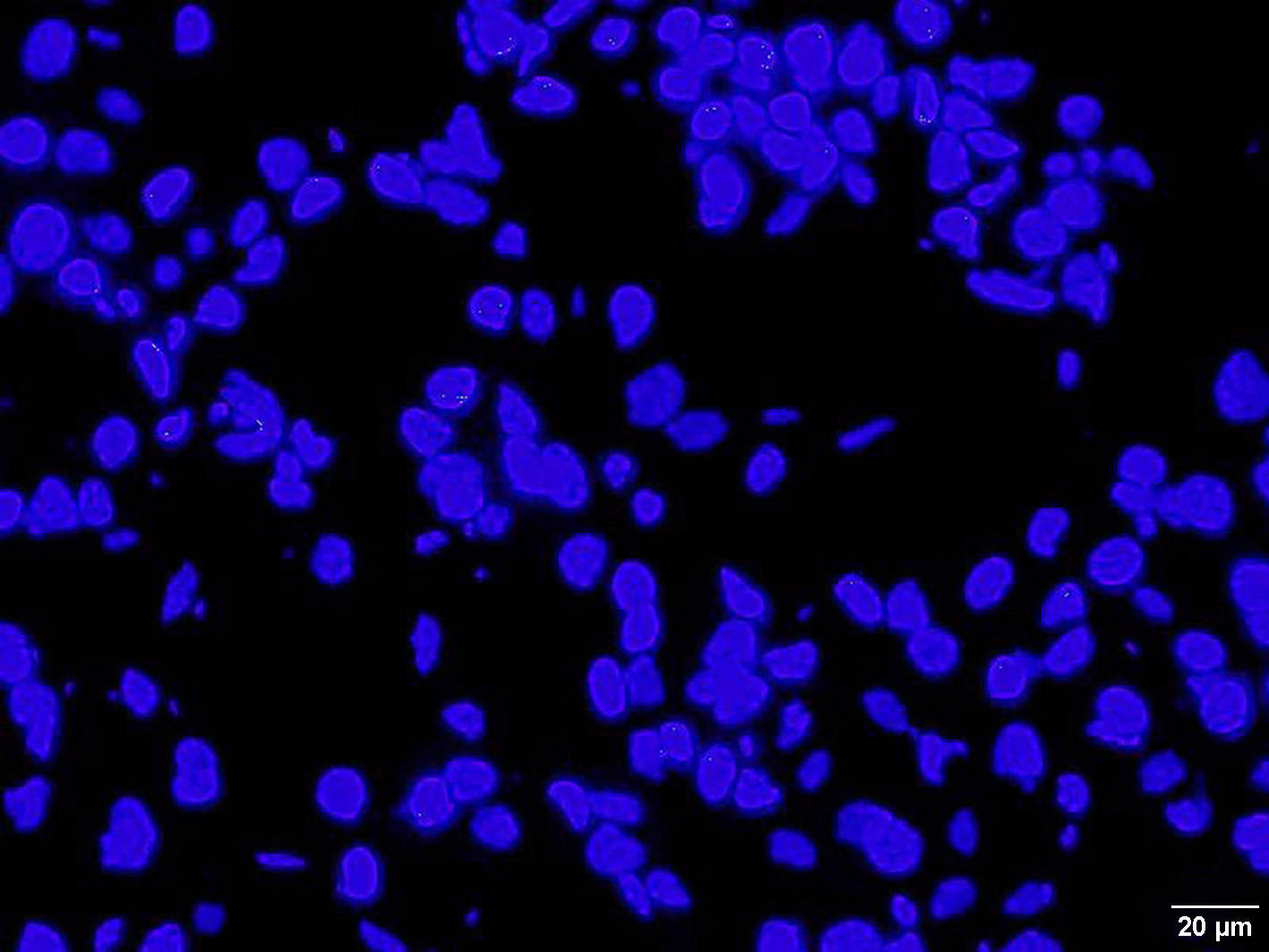

Supplement: Supplementary file 1 [file Data_Sheet_1.ZIP › Original IF images/Control magnification × 100/BLVRB/DAPI.jpg]

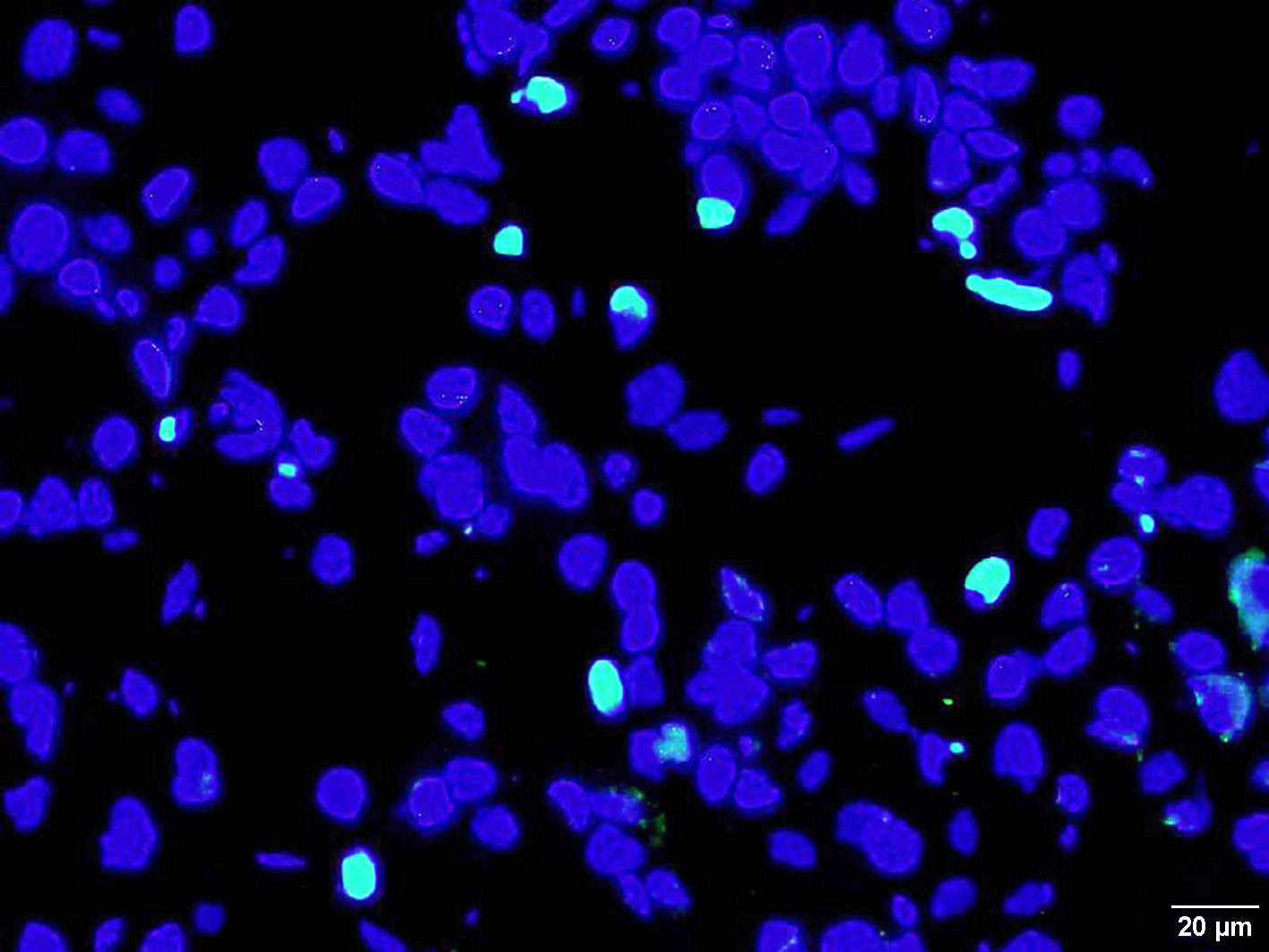

Supplement: Supplementary file 1 [file Data_Sheet_1.ZIP › Original IF images/Control magnification × 100/BLVRB/Merge.jpg]

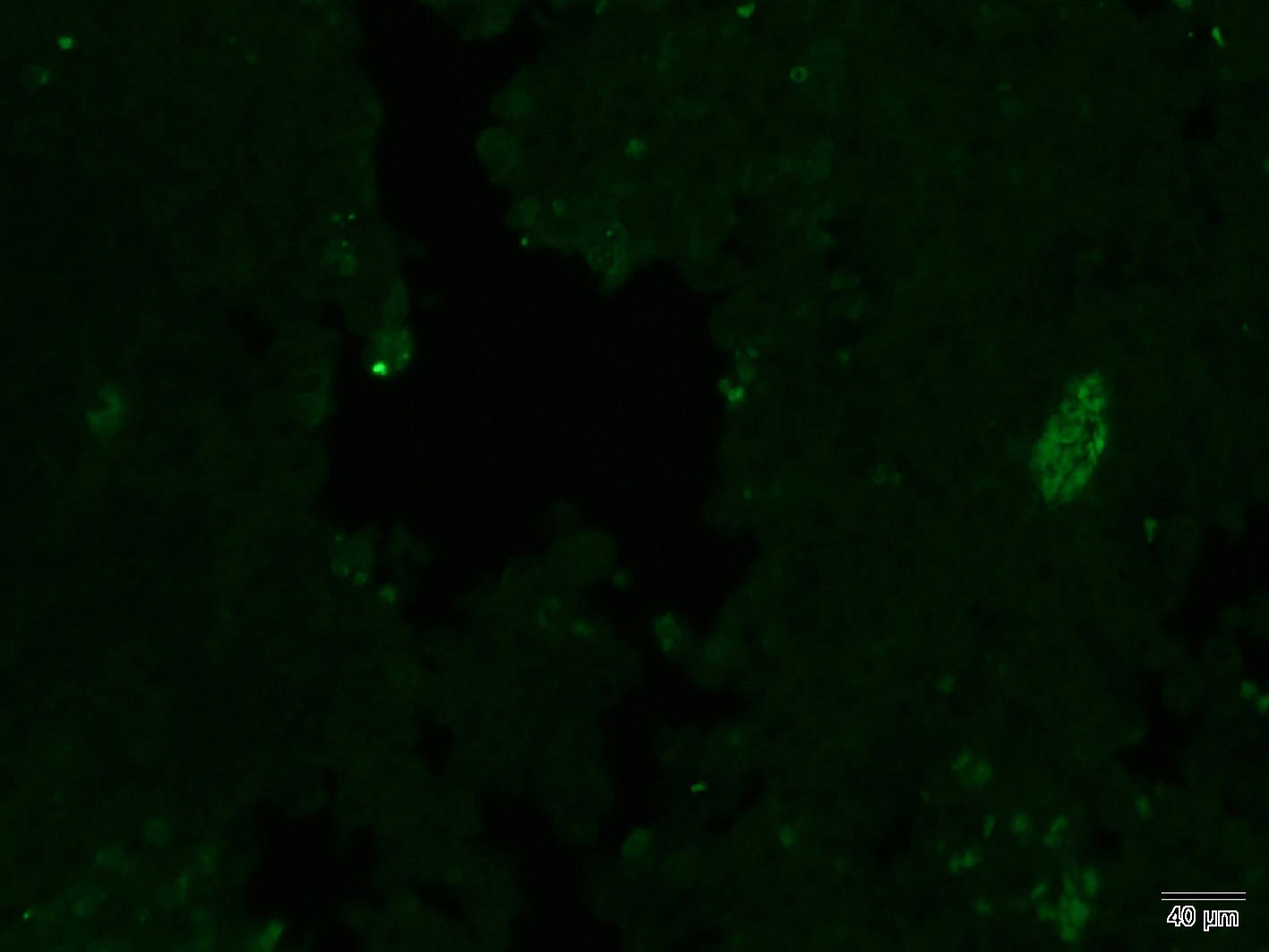

Supplement: Supplementary file 1 [file Data_Sheet_1.ZIP › Original IF images/Control magnification × 100/ATF4/ATF4.jpg]

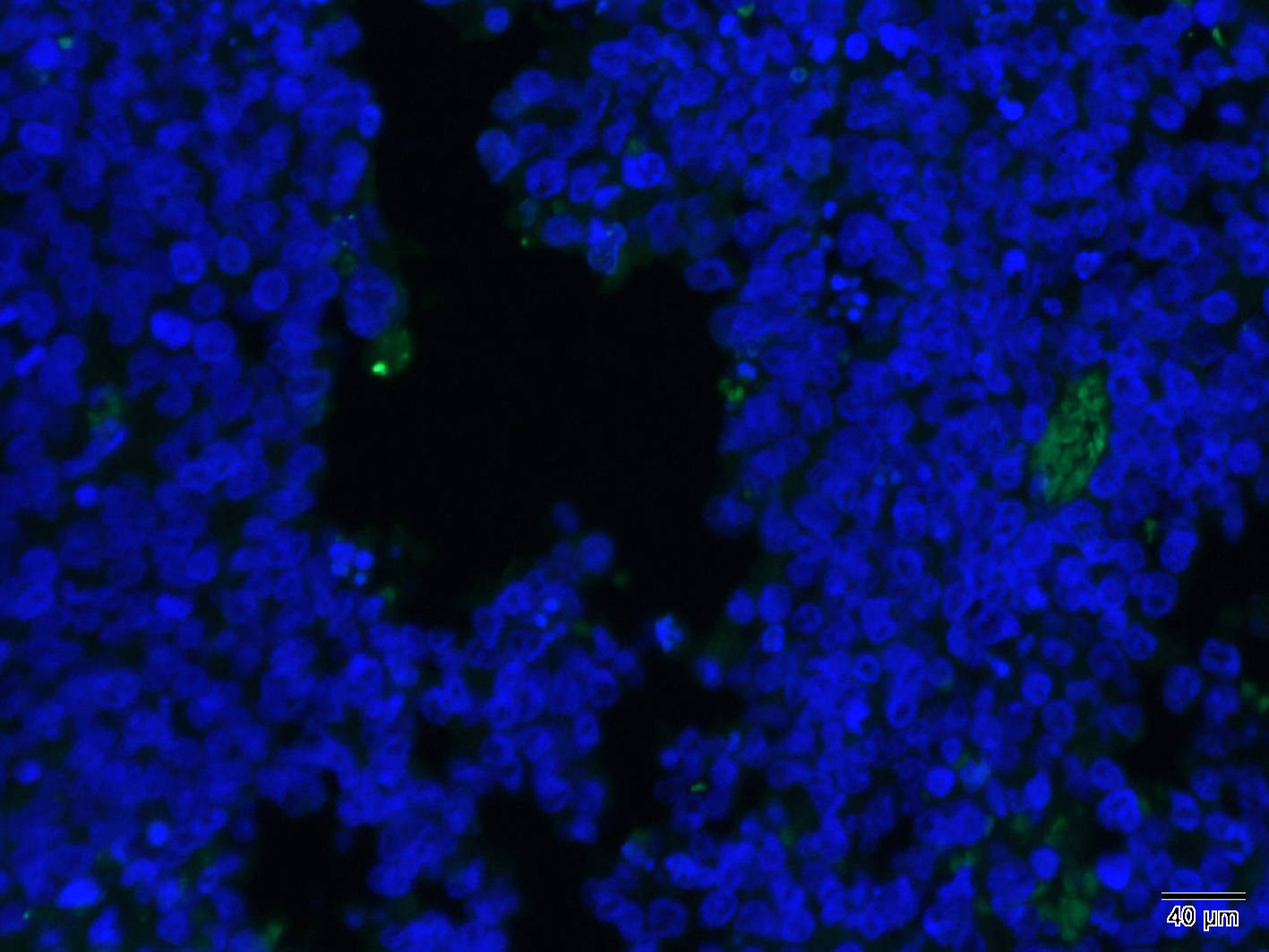

Supplement: Supplementary file 1 [file Data_Sheet_1.ZIP › Original IF images/Control magnification × 100/ATF4/Merge.jpg]

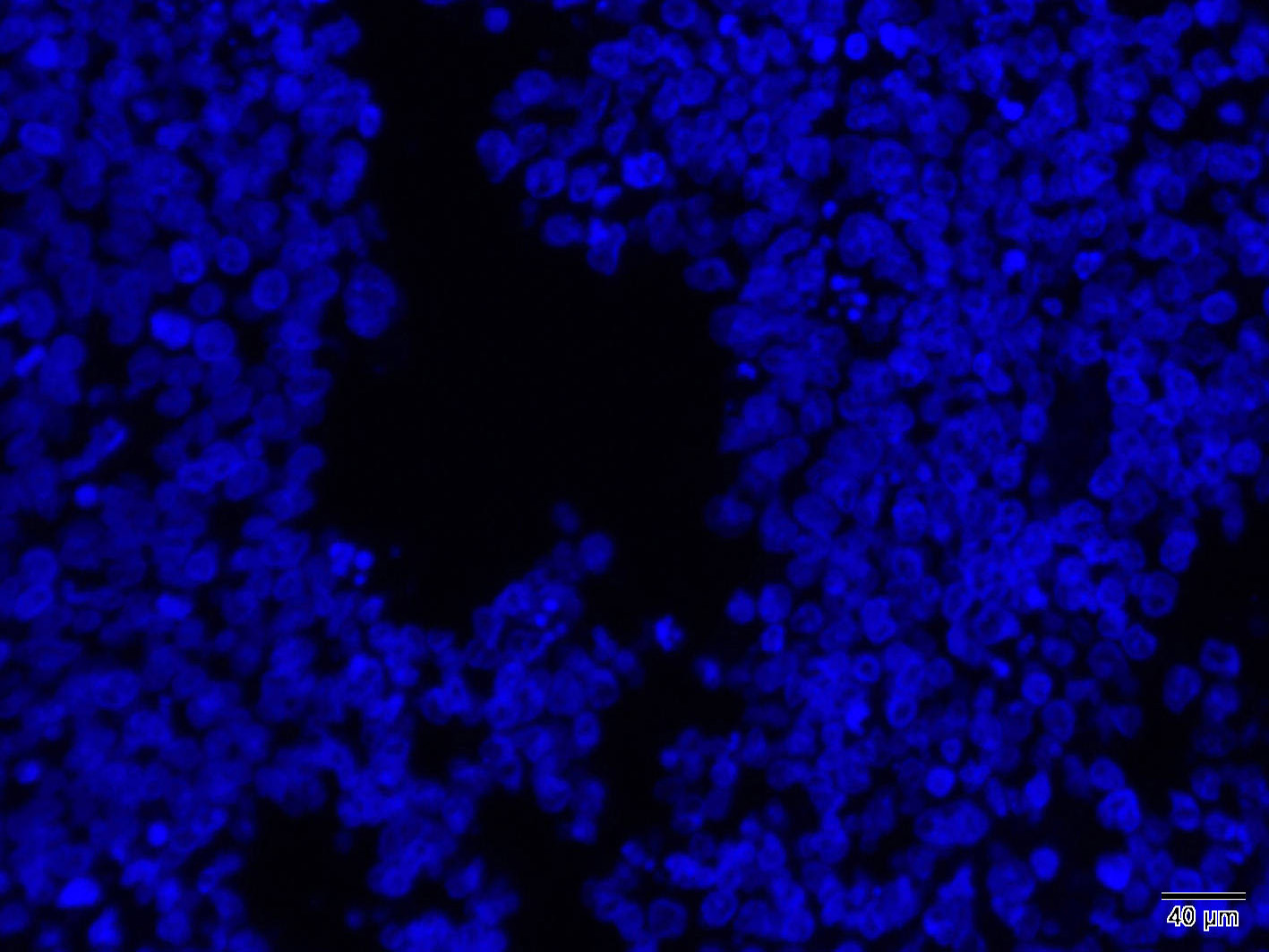

Supplement: Supplementary file 1 [file Data_Sheet_1.ZIP › Original IF images/Control magnification × 100/ATF4/DAPI.jpg]

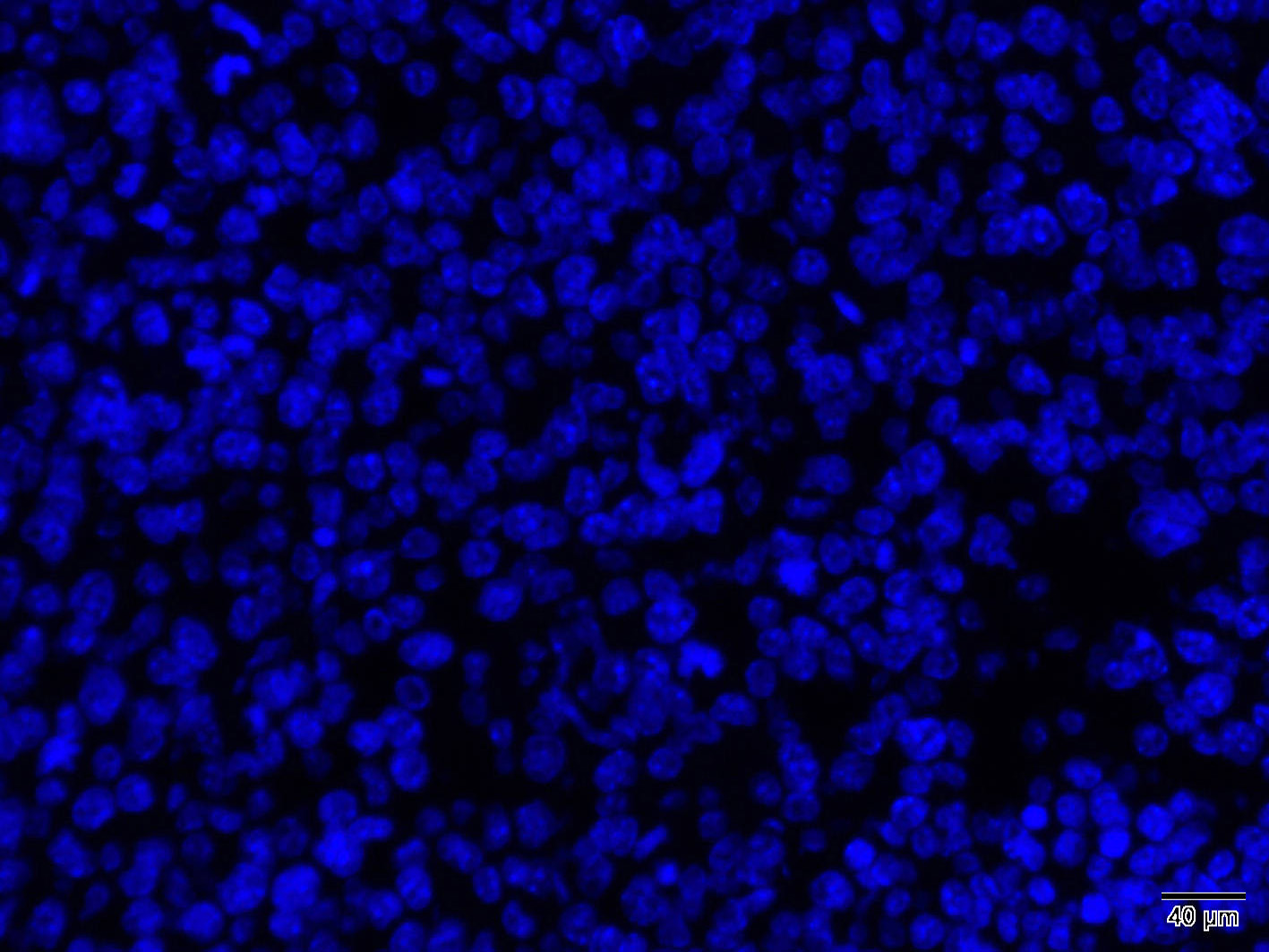

Supplement: Supplementary file 1 [file Data_Sheet_1.ZIP › Original IF images/Control magnification × 100/ITGAX/DAPI.jpg]

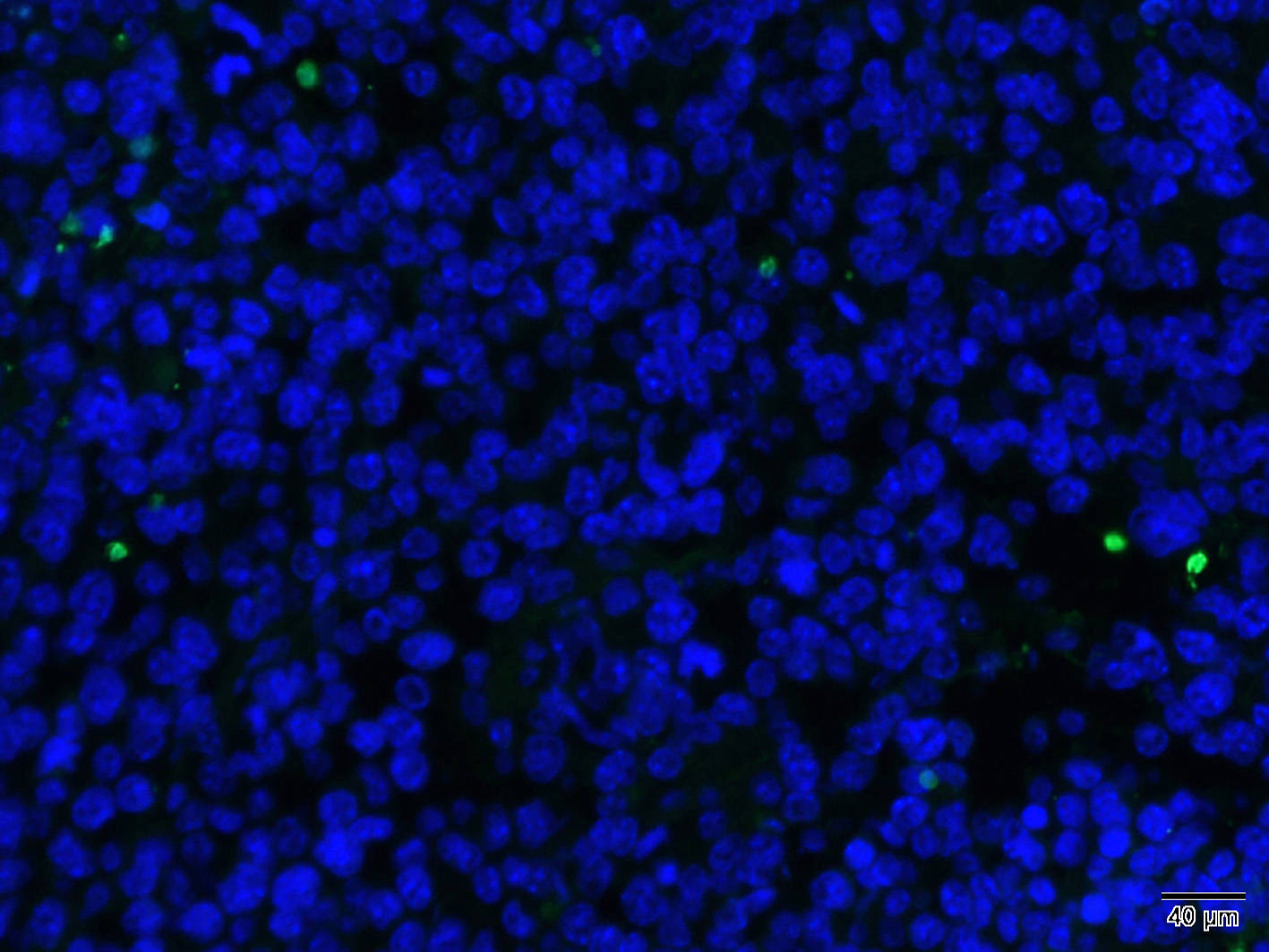

Supplement: Supplementary file 1 [file Data_Sheet_1.ZIP › Original IF images/Control magnification × 100/ITGAX/Merge.jpg]

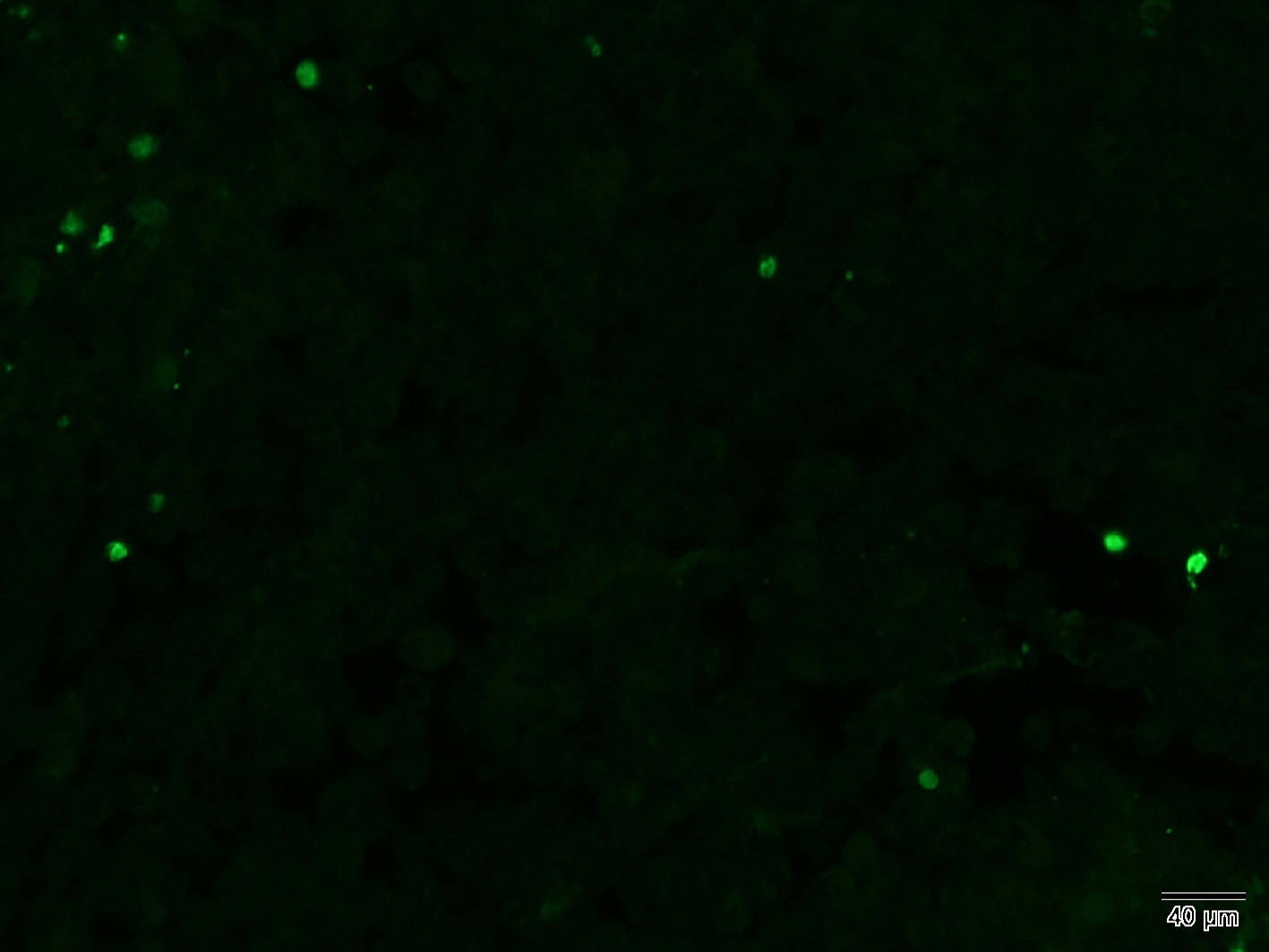

Supplement: Supplementary file 1 [file Data_Sheet_1.ZIP › Original IF images/Control magnification × 100/ITGAX/ITGAX.jpg]

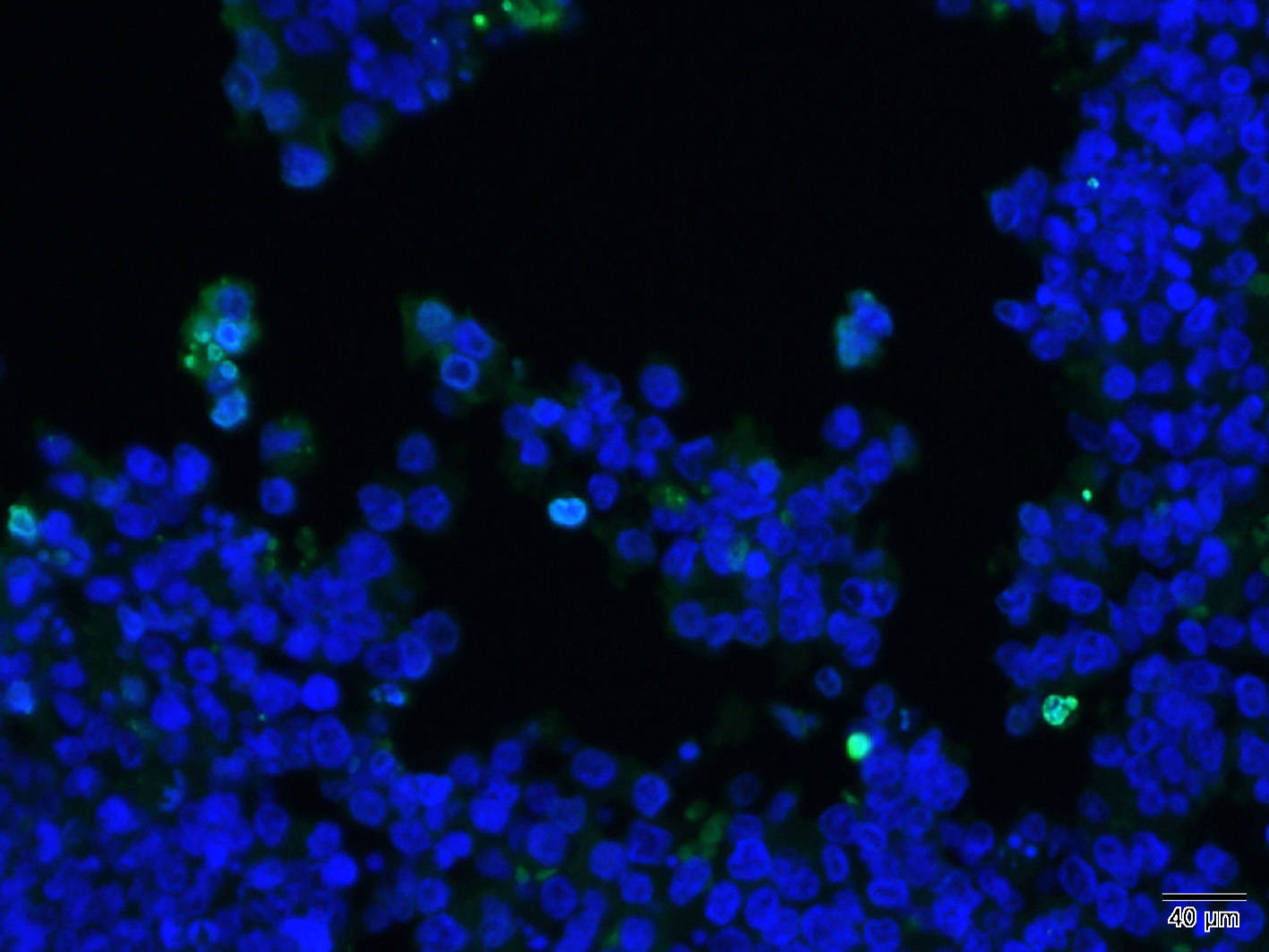

Supplement: Supplementary file 1 [file Data_Sheet_1.ZIP › Original IF images/ICH+FMT magnification × 100/ATF4/Merge.jpg]

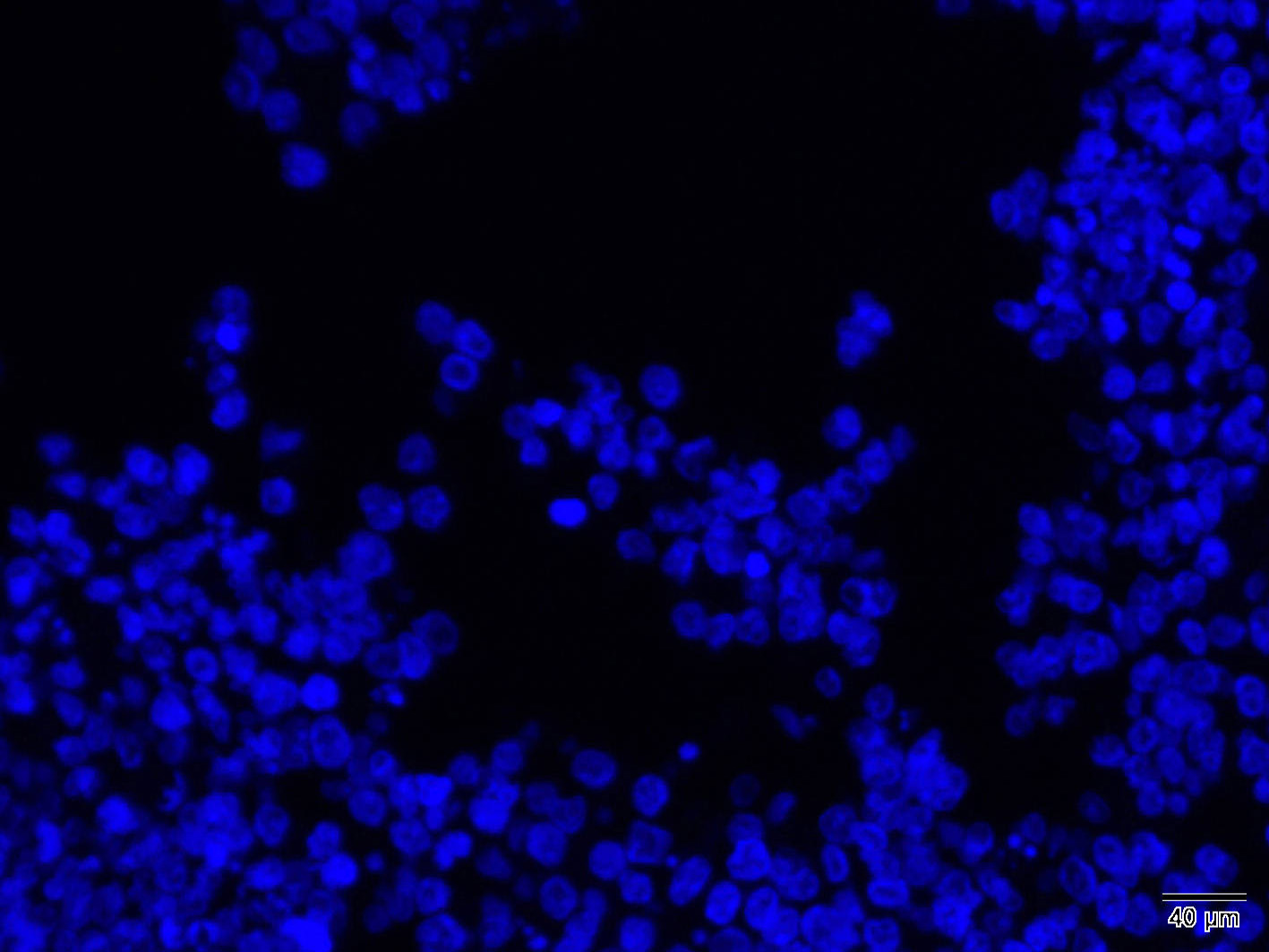

Supplement: Supplementary file 1 [file Data_Sheet_1.ZIP › Original IF images/ICH+FMT magnification × 100/ATF4/DAPI.jpg]

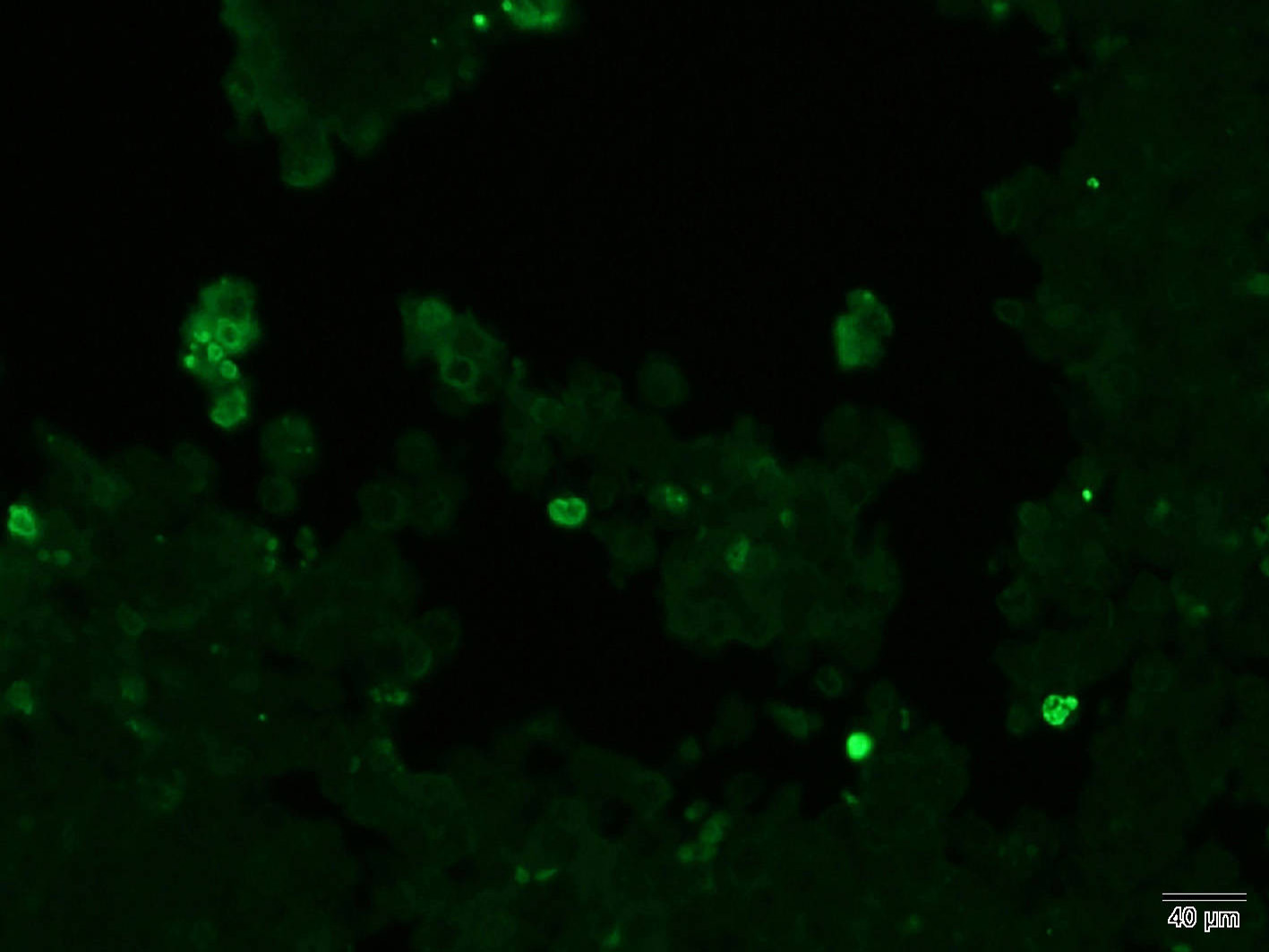

Supplement: Supplementary file 1 [file Data_Sheet_1.ZIP › Original IF images/ICH+FMT magnification × 100/ATF4/ATF4.jpg]

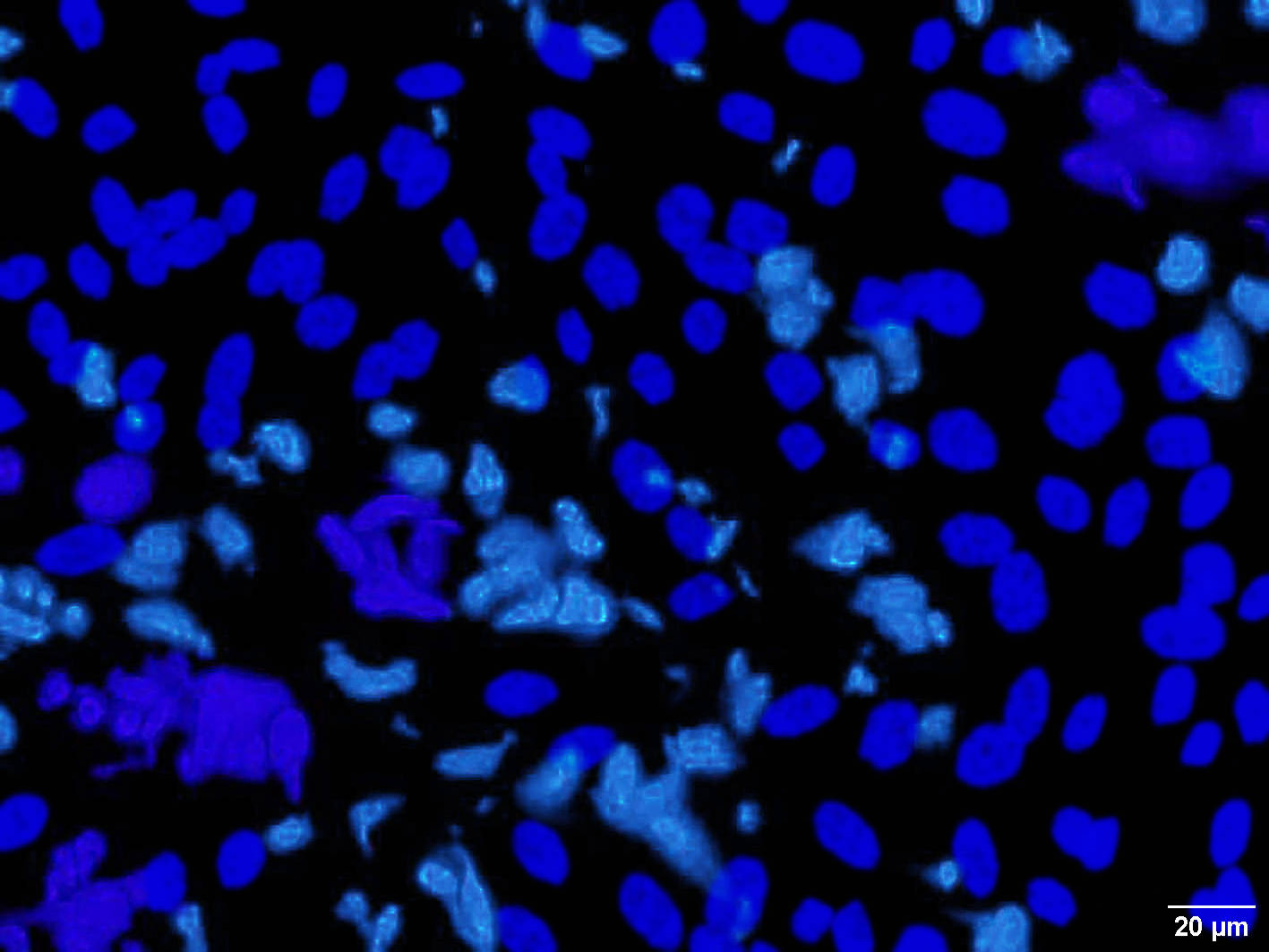

Supplement: Supplementary file 1 [file Data_Sheet_1.ZIP › Original IF images/ICH+FMT magnification × 100/BLVRB/Merge.jpg]

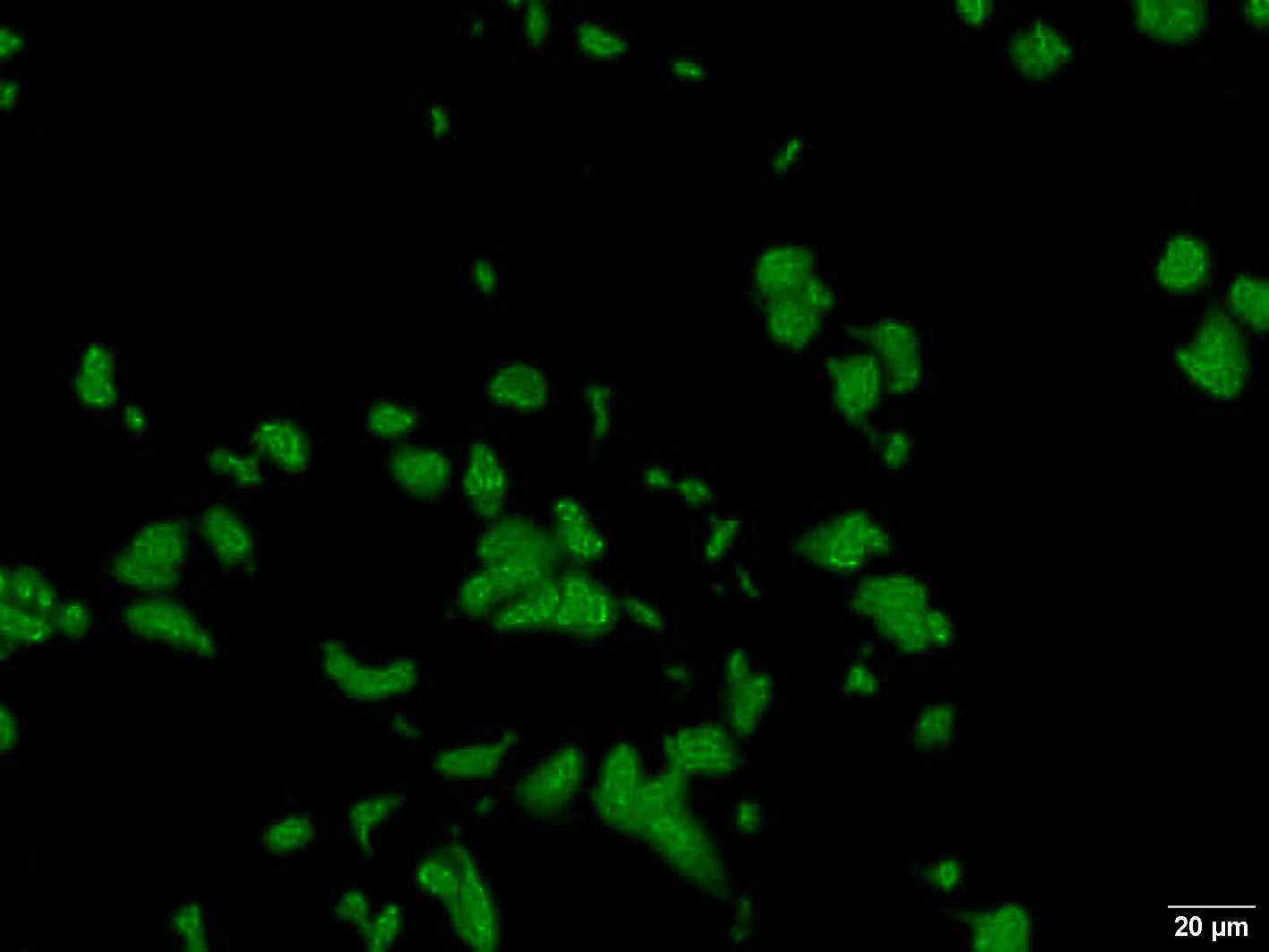

Supplement: Supplementary file 1 [file Data_Sheet_1.ZIP › Original IF images/ICH+FMT magnification × 100/BLVRB/BLVRB.jpg]

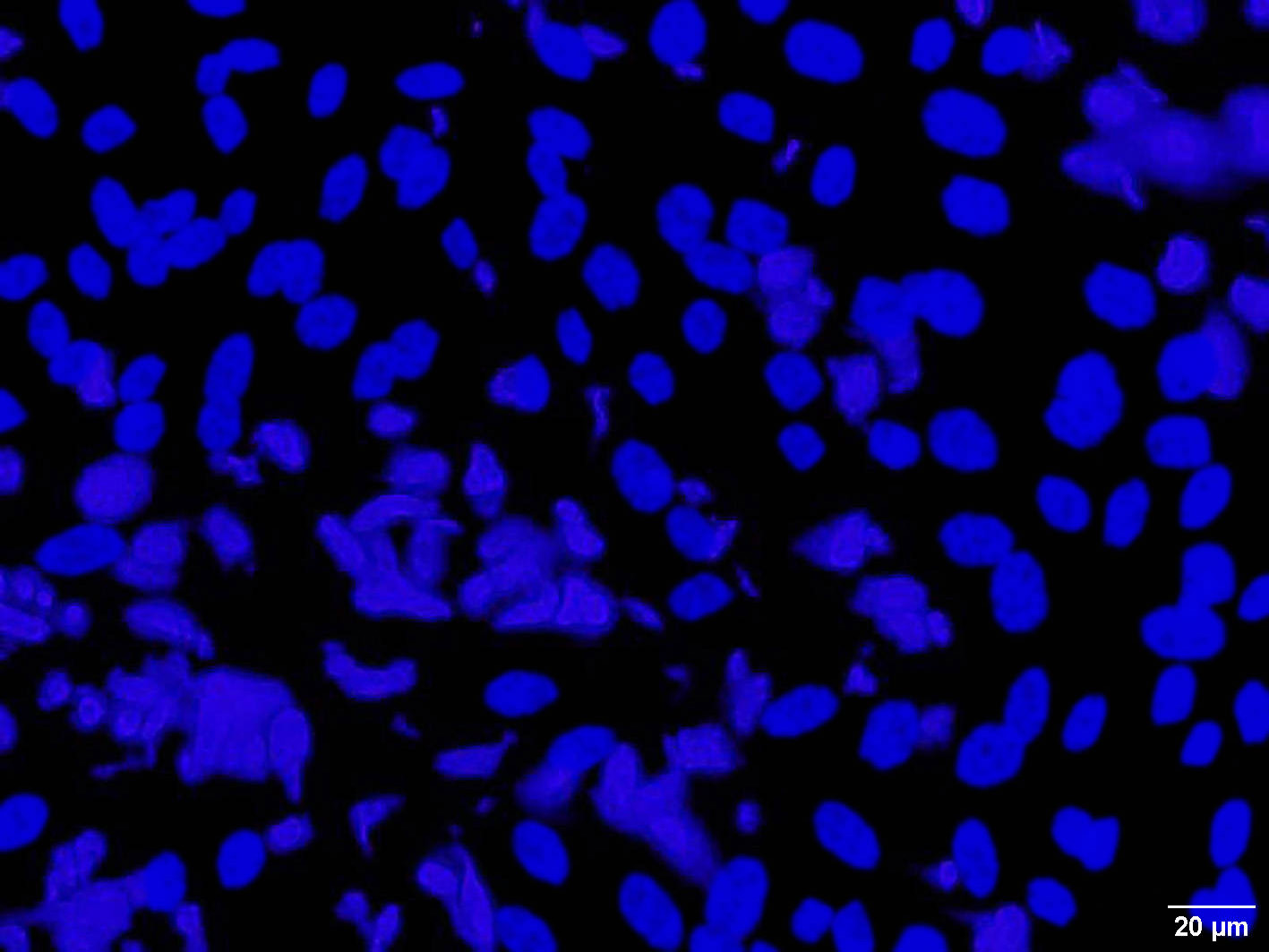

Supplement: Supplementary file 1 [file Data_Sheet_1.ZIP › Original IF images/ICH+FMT magnification × 100/BLVRB/DAPI.jpg]

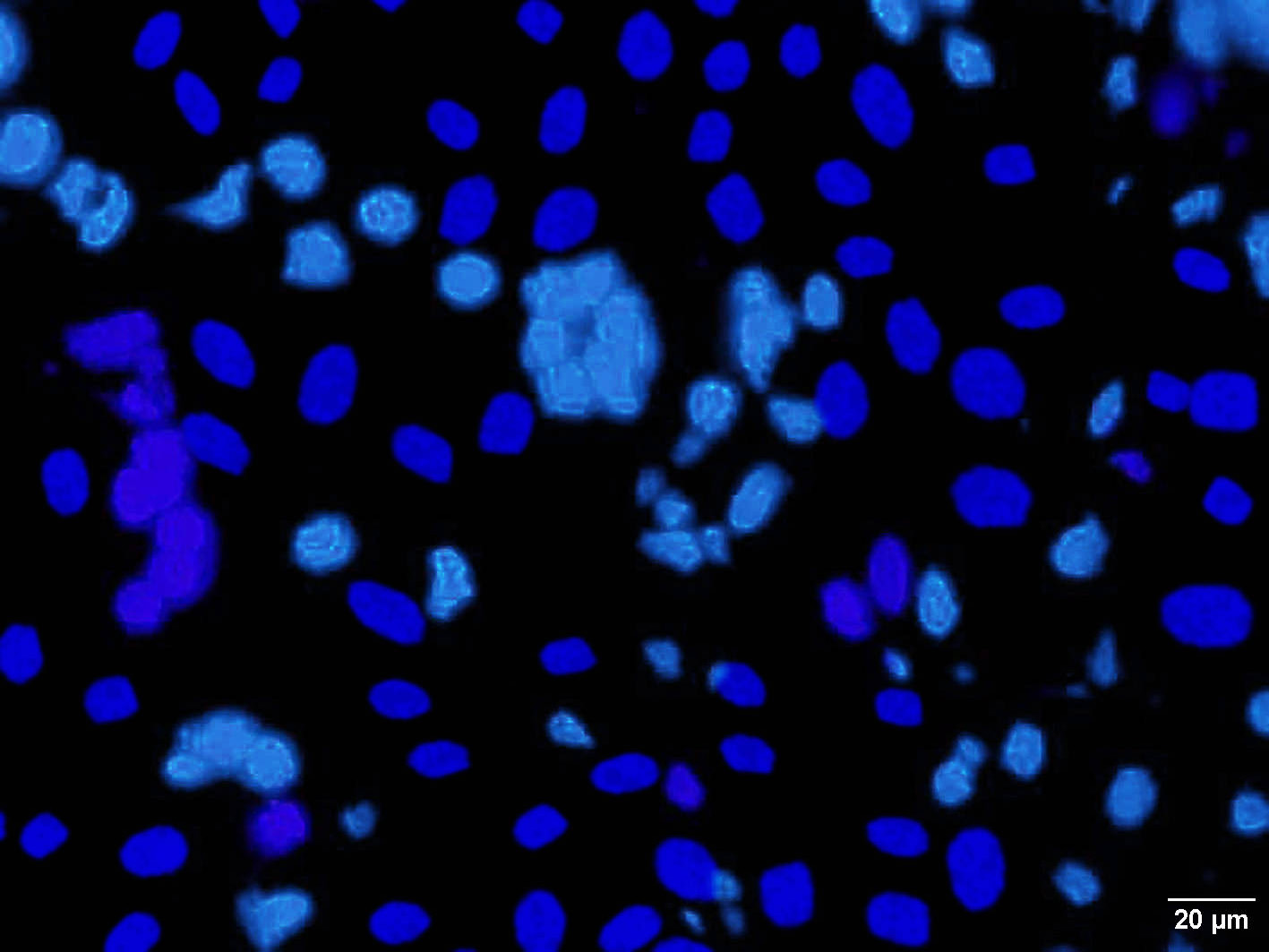

Supplement: Supplementary file 1 [file Data_Sheet_1.ZIP › Original IF images/ICH+FMT magnification × 100/LEF1/Merge.jpg]

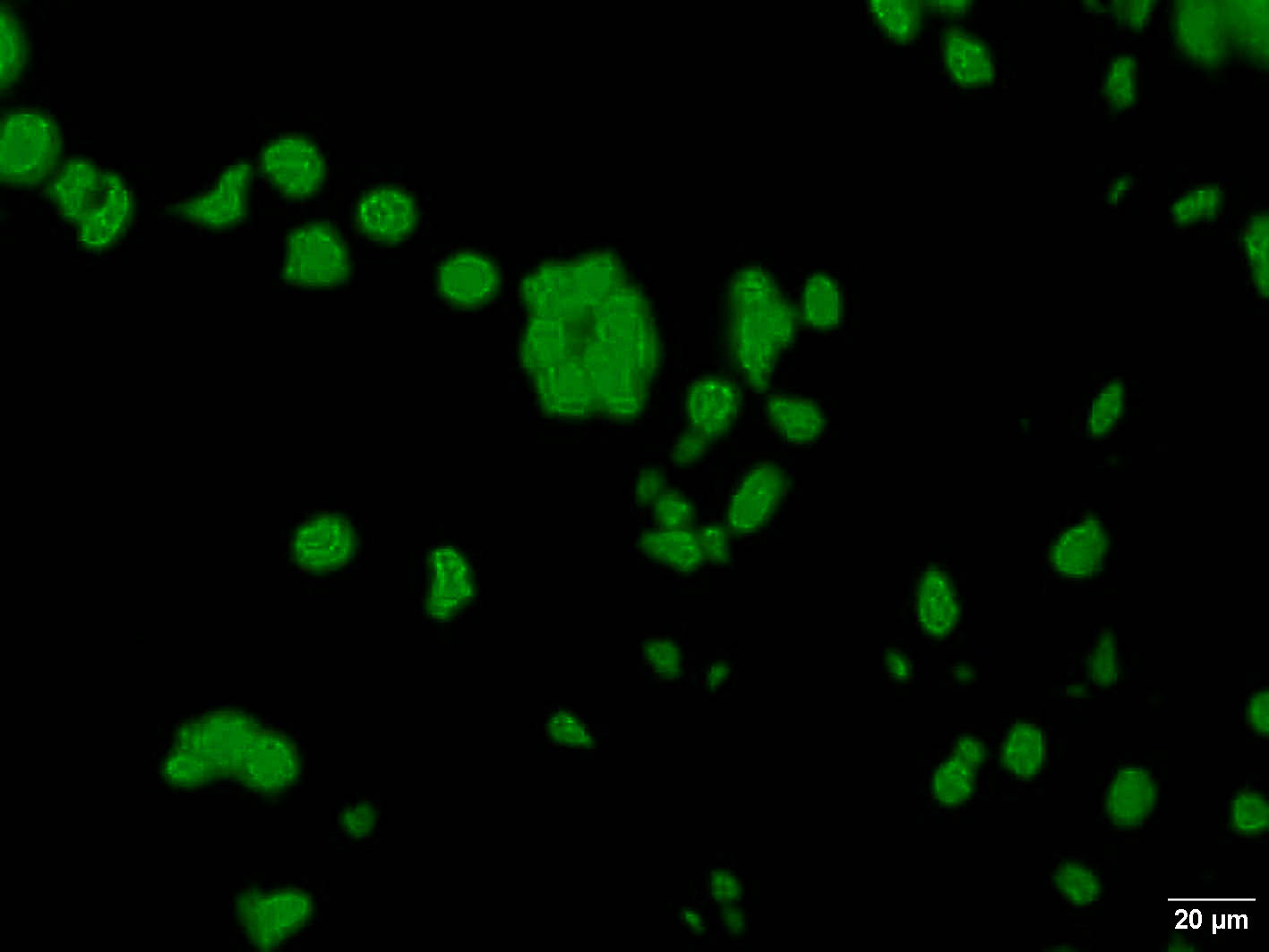

Supplement: Supplementary file 1 [file Data_Sheet_1.ZIP › Original IF images/ICH+FMT magnification × 100/LEF1/LEF1.jpg]

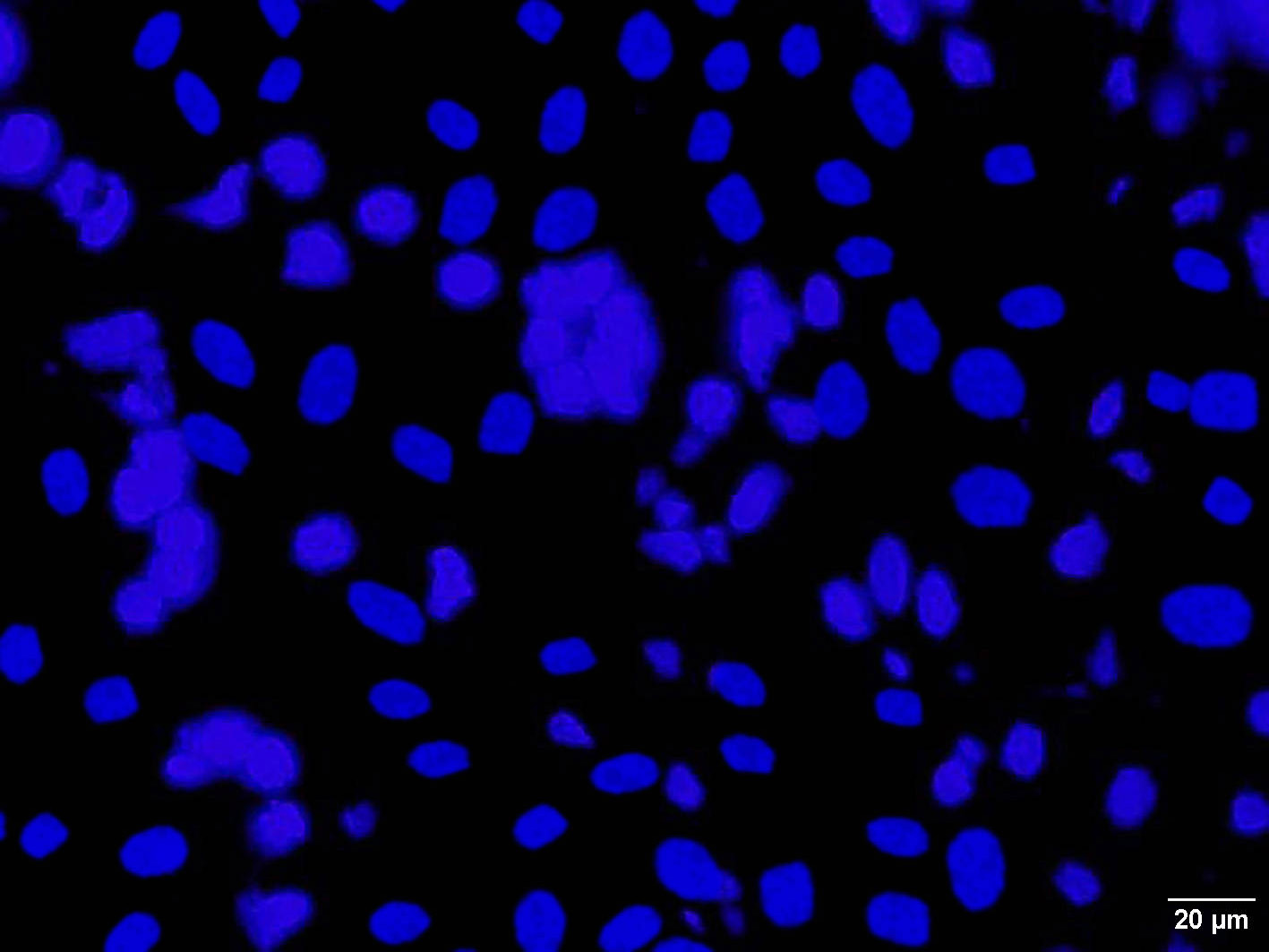

Supplement: Supplementary file 1 [file Data_Sheet_1.ZIP › Original IF images/ICH+FMT magnification × 100/LEF1/DAPI.jpg]

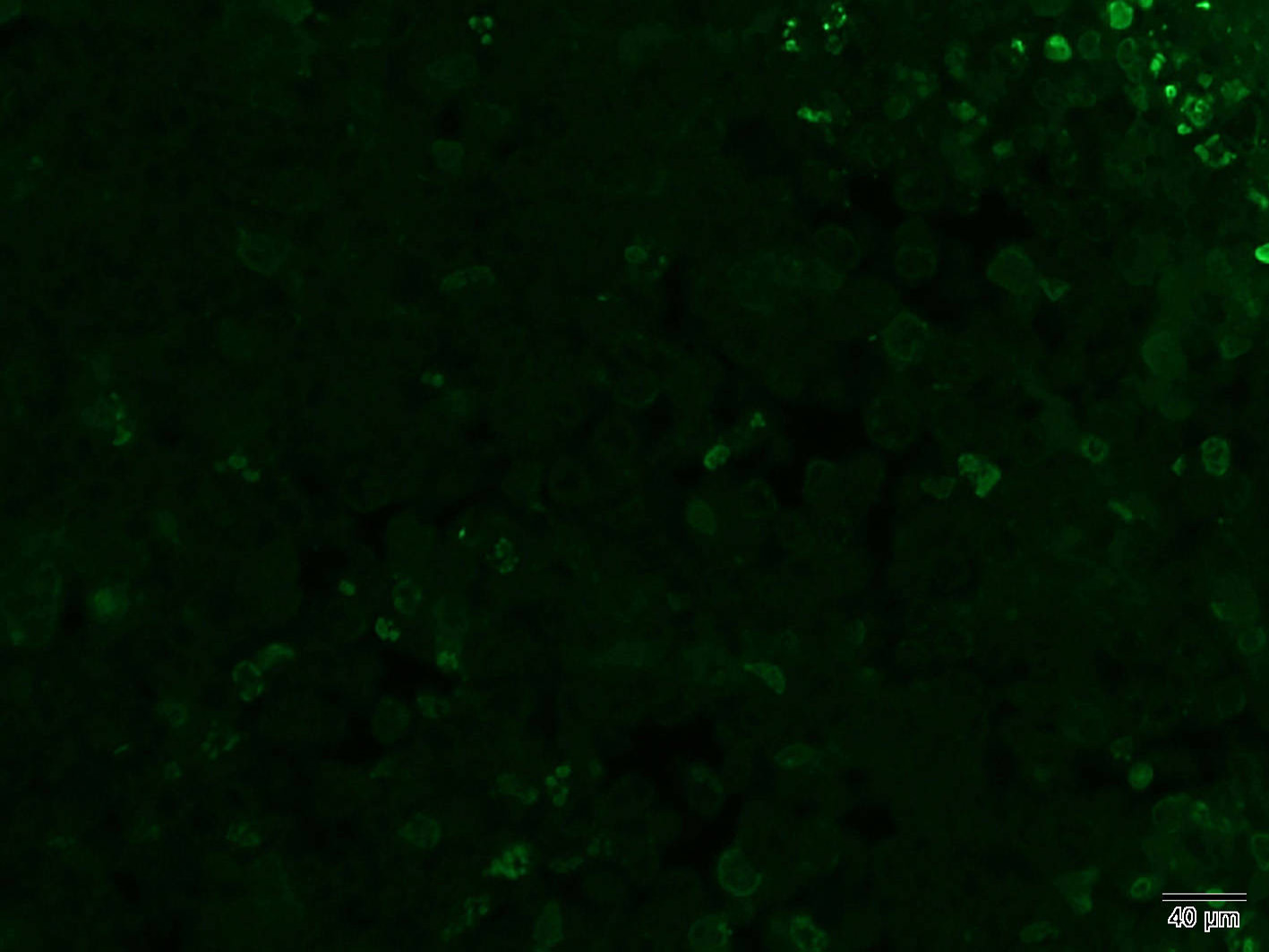

Supplement: Supplementary file 1 [file Data_Sheet_1.ZIP › Original IF images/ICH+FMT magnification × 100/ITGAX/ITGAX.jpg]

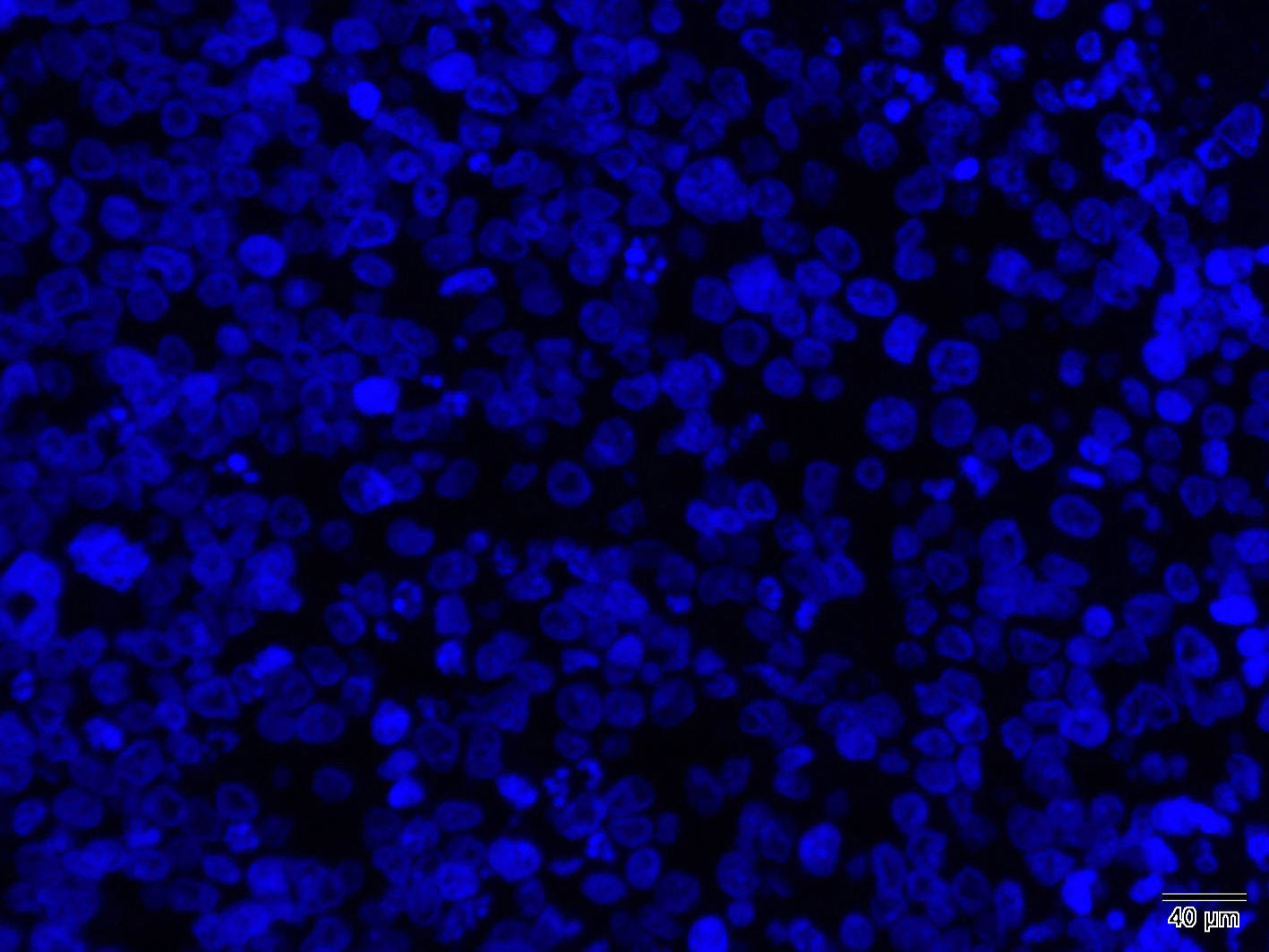

Supplement: Supplementary file 1 [file Data_Sheet_1.ZIP › Original IF images/ICH+FMT magnification × 100/ITGAX/DAPI.jpg]

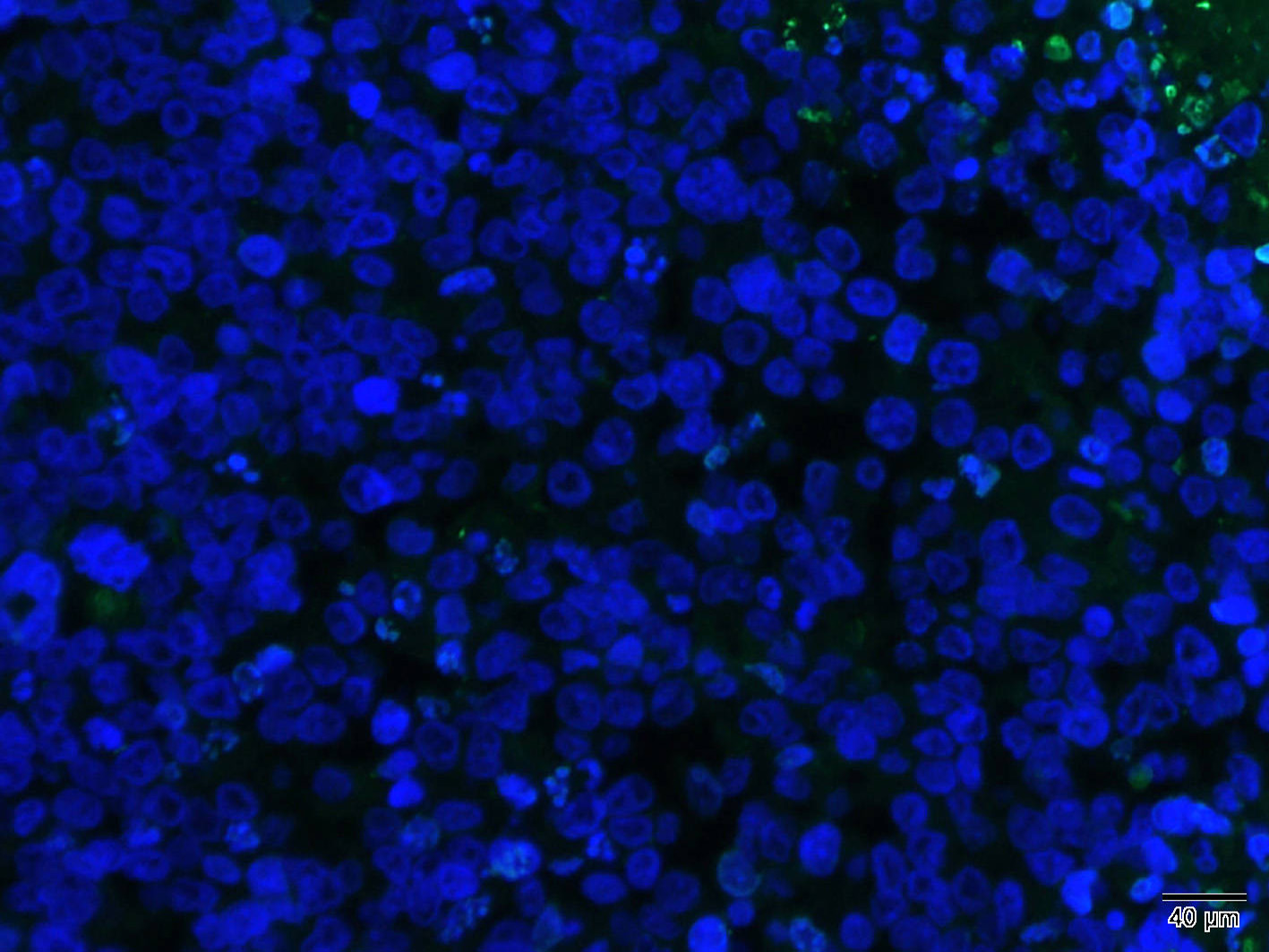

Supplement: Supplementary file 1 [file Data_Sheet_1.ZIP › Original IF images/ICH+FMT magnification × 100/ITGAX/Merge.jpg]

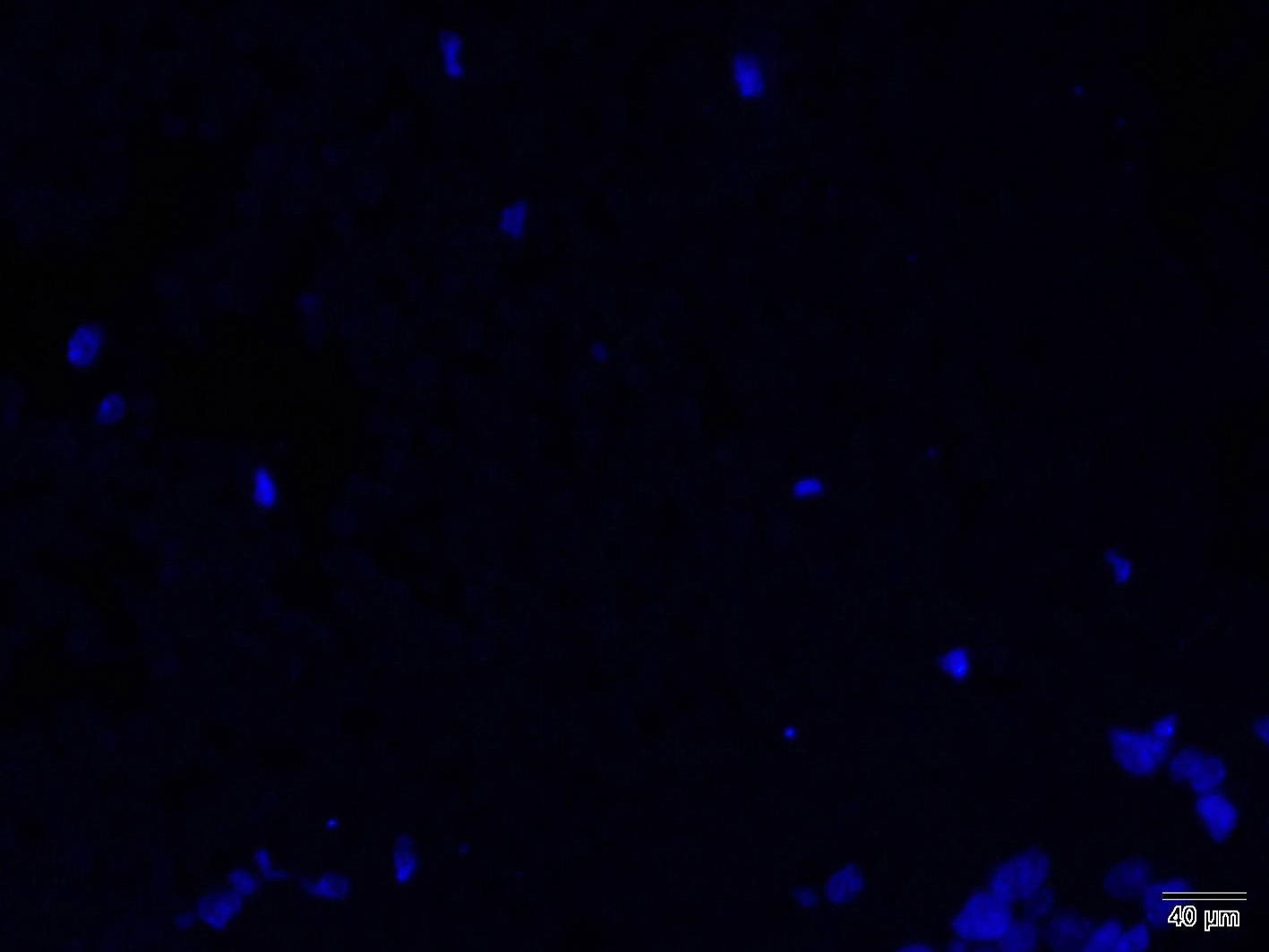

Supplement: Supplementary file 1 [file Data_Sheet_1.ZIP › Original IF images/ICH magnification × 100/BLVRB/DAPI.jpg]

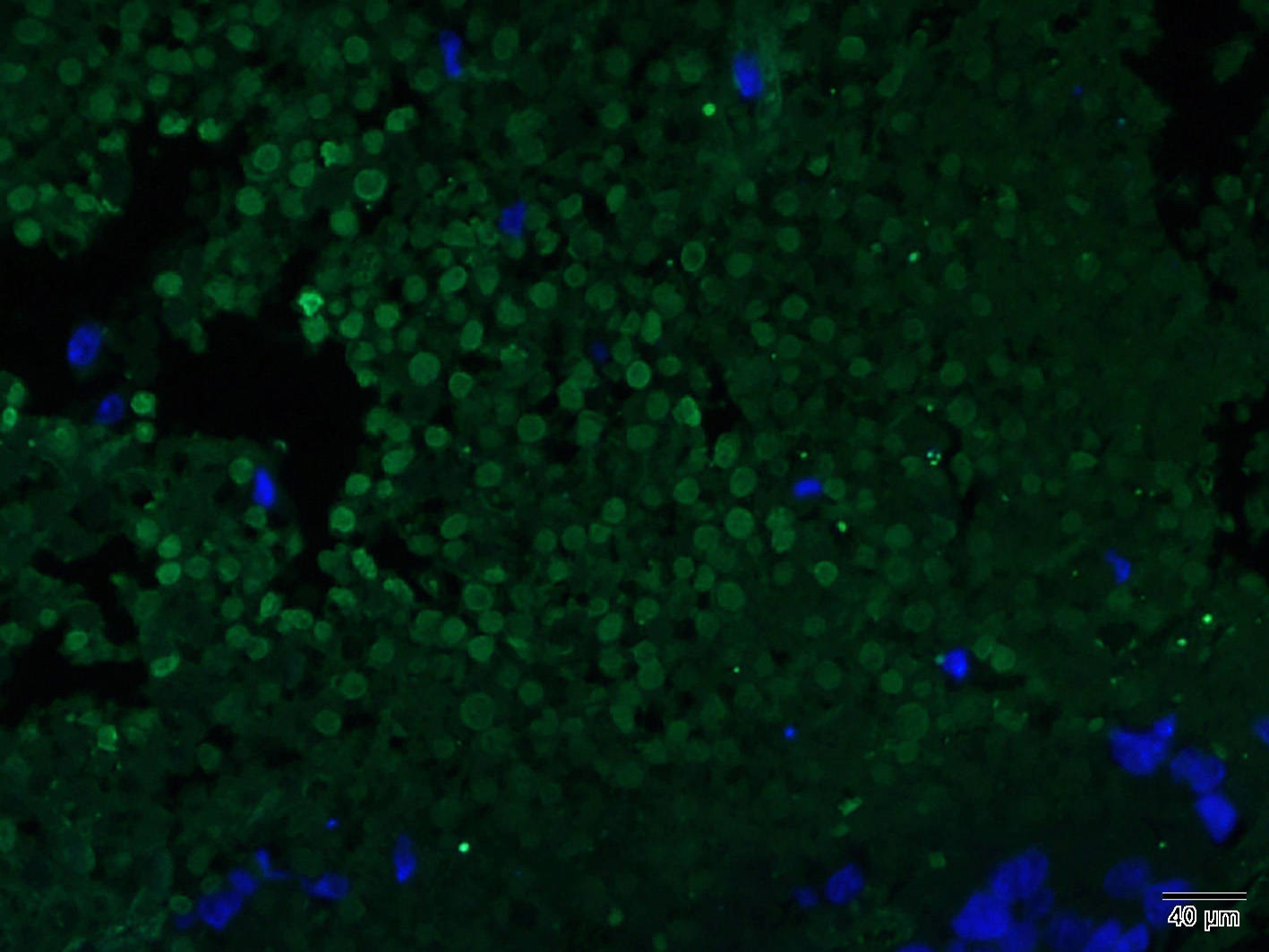

Supplement: Supplementary file 1 [file Data_Sheet_1.ZIP › Original IF images/ICH magnification × 100/BLVRB/Merge.jpg]

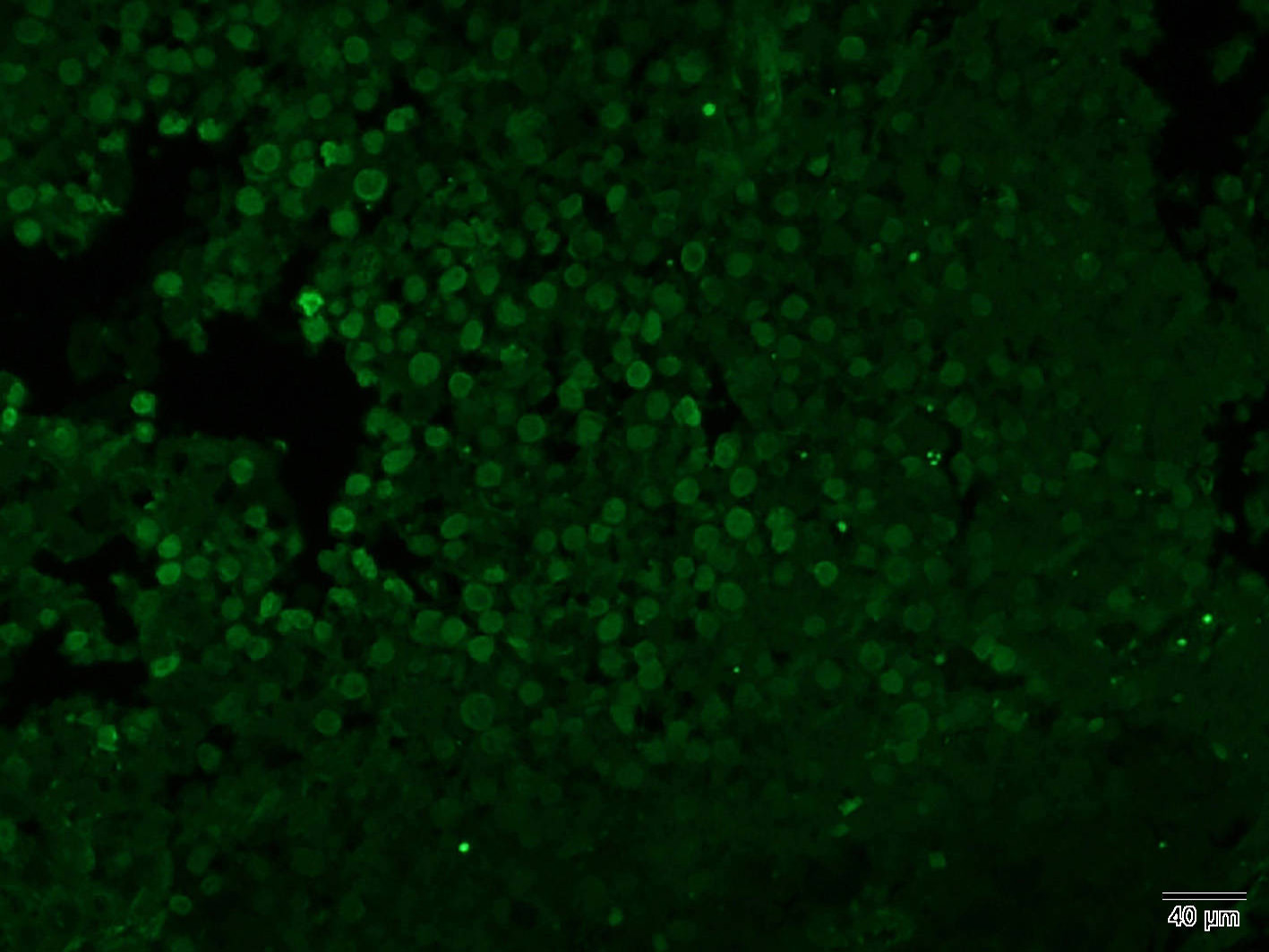

Supplement: Supplementary file 1 [file Data_Sheet_1.ZIP › Original IF images/ICH magnification × 100/BLVRB/BLVRB.jpg]

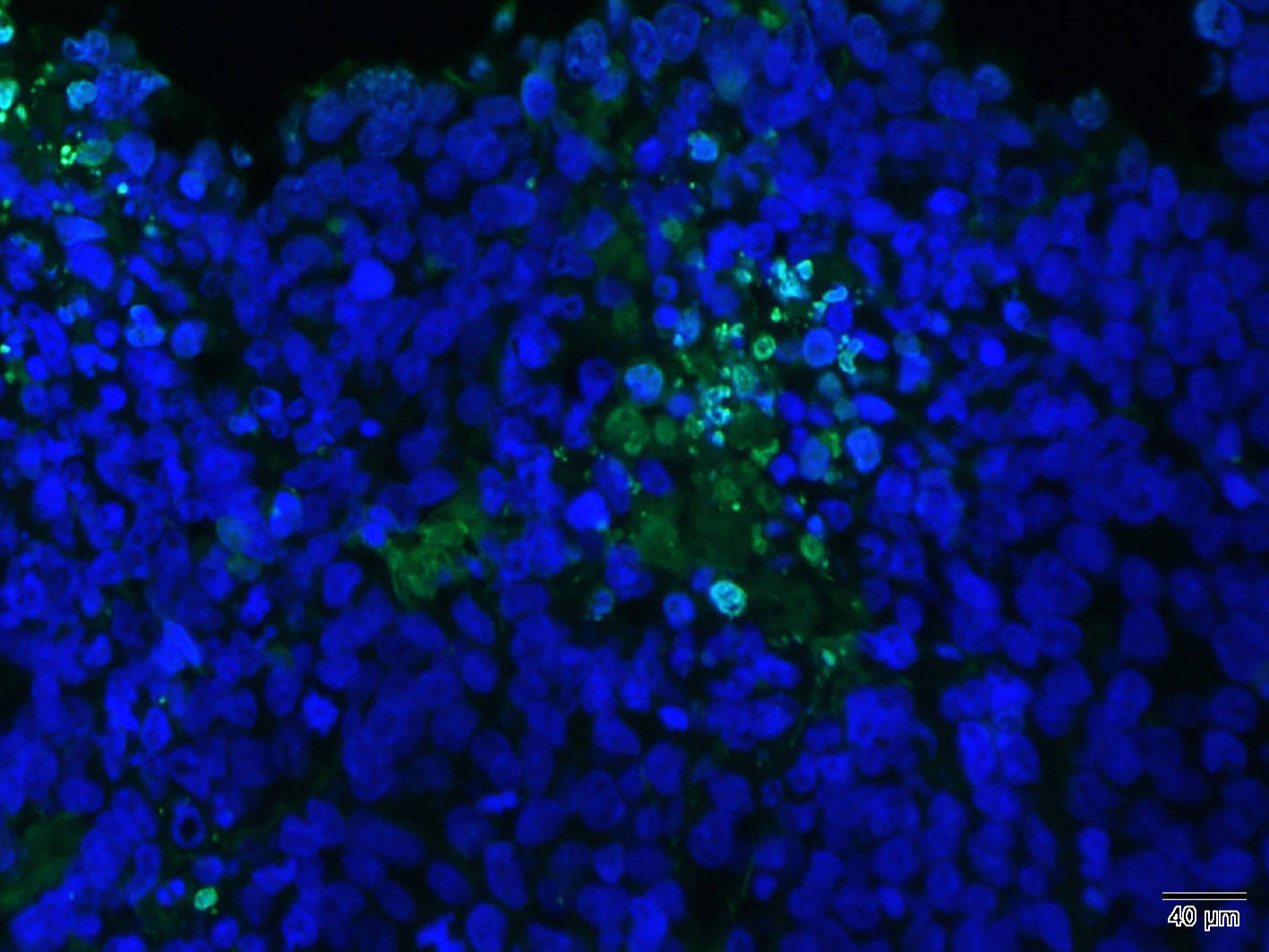

Supplement: Supplementary file 1 [file Data_Sheet_1.ZIP › Original IF images/ICH magnification × 100/ITGAX/Merge.jpg]

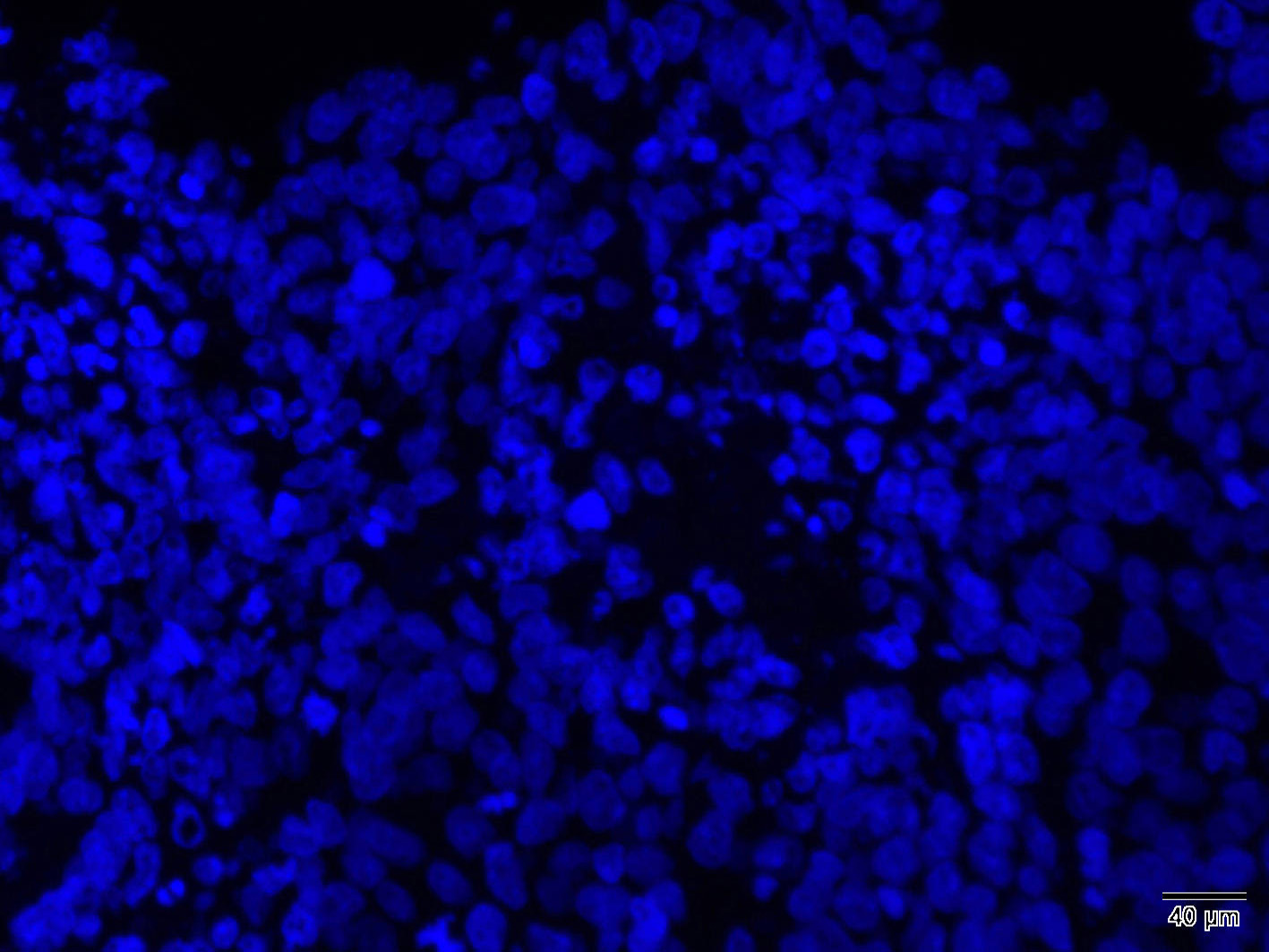

Supplement: Supplementary file 1 [file Data_Sheet_1.ZIP › Original IF images/ICH magnification × 100/ITGAX/DAPI.jpg]

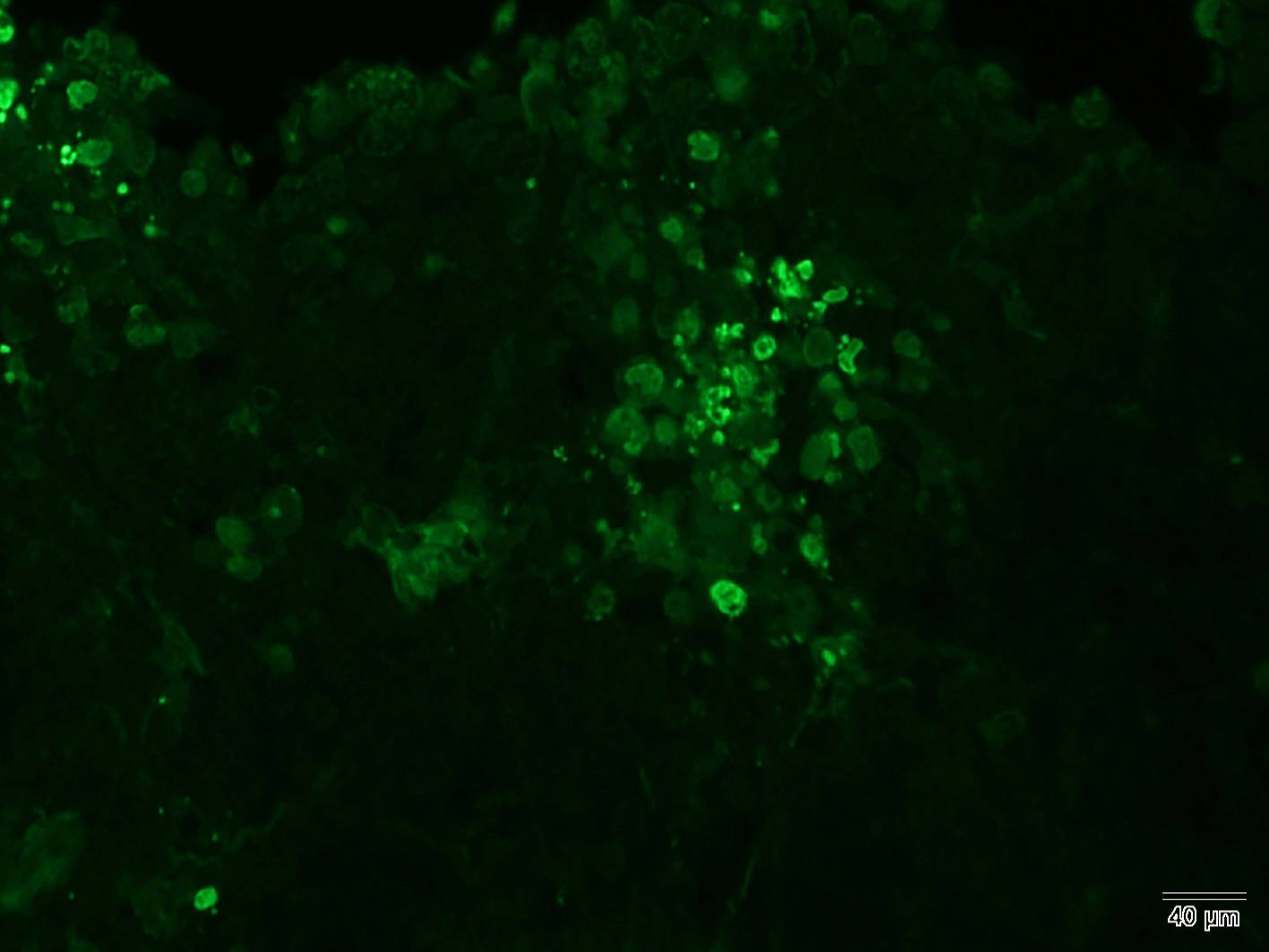

Supplement: Supplementary file 1 [file Data_Sheet_1.ZIP › Original IF images/ICH magnification × 100/ITGAX/ITGAX.jpg]

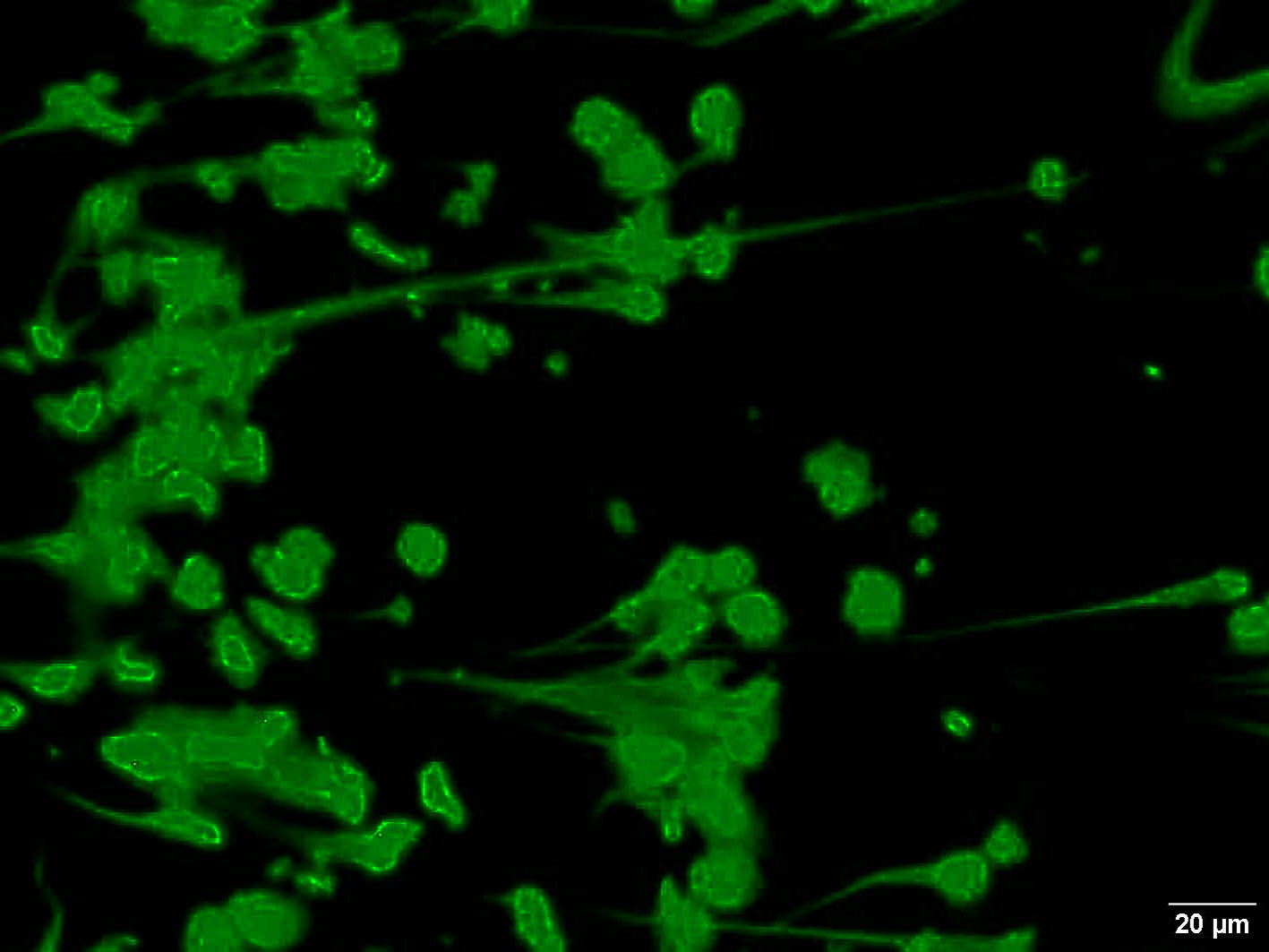

Supplement: Supplementary file 1 [file Data_Sheet_1.ZIP › Original IF images/ICH magnification × 100/ATF4/ATF4.jpg]

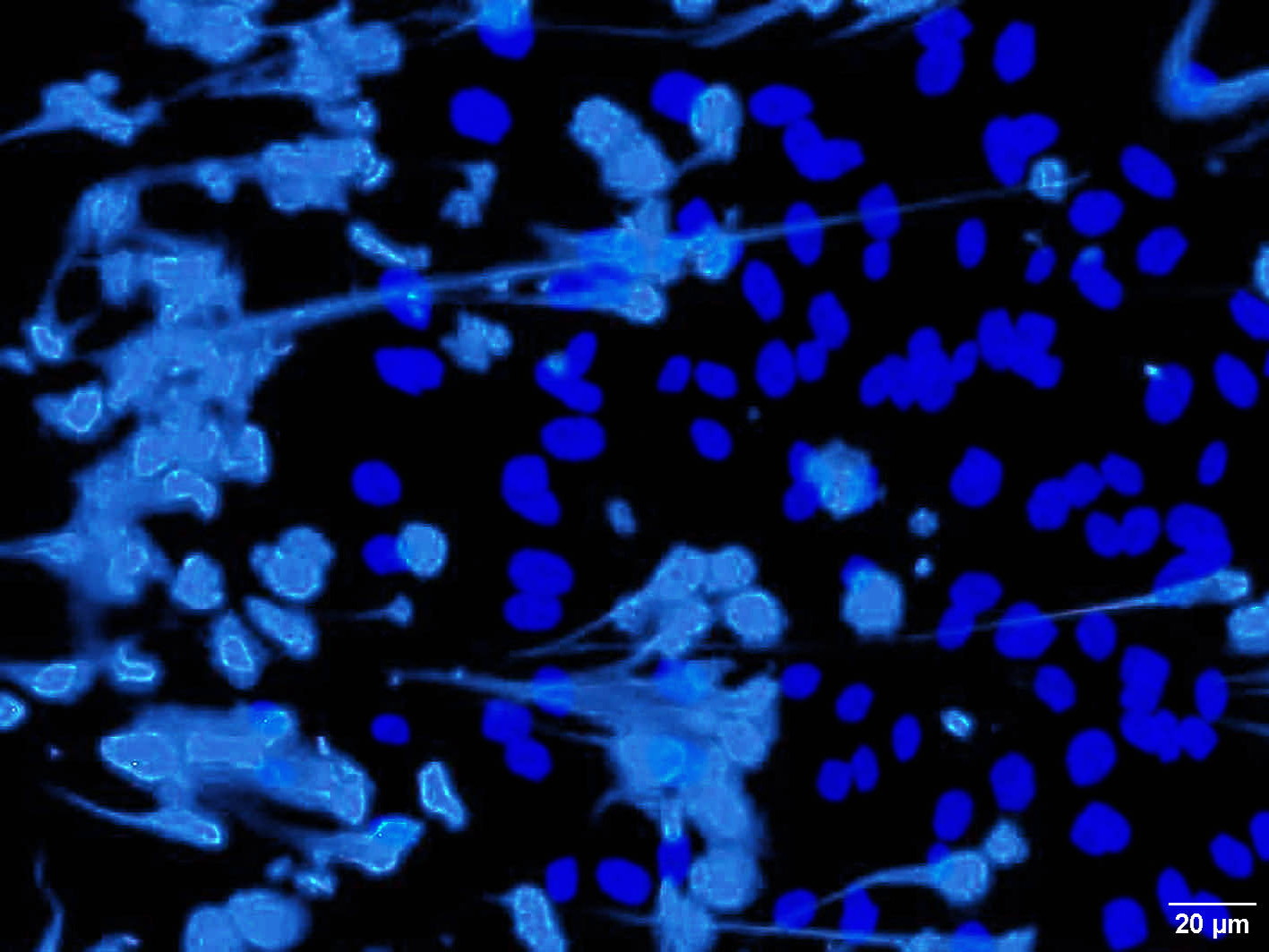

Supplement: Supplementary file 1 [file Data_Sheet_1.ZIP › Original IF images/ICH magnification × 100/ATF4/Merge.jpg]

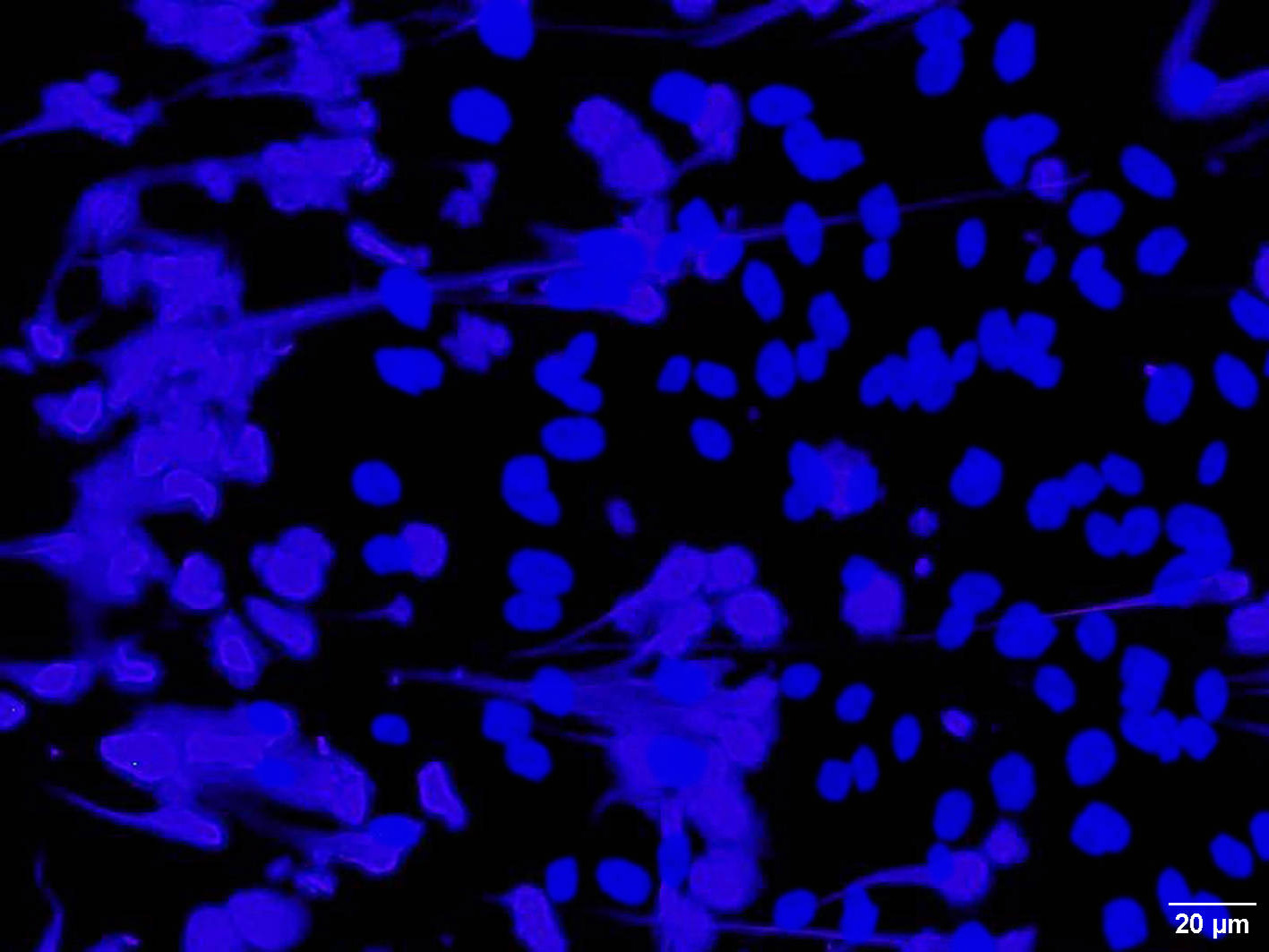

Supplement: Supplementary file 1 [file Data_Sheet_1.ZIP › Original IF images/ICH magnification × 100/ATF4/DAPI.jpg]

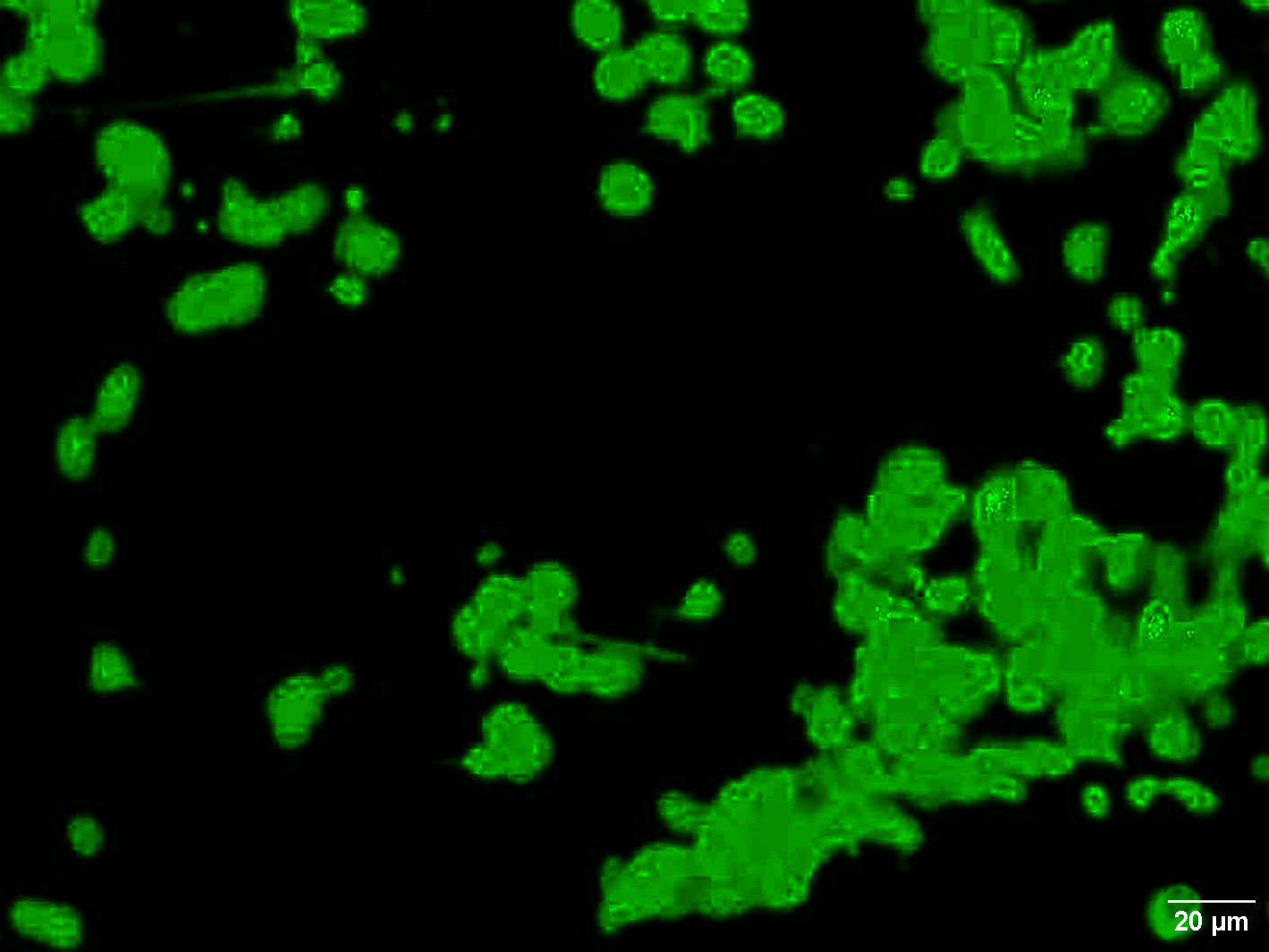

Supplement: Supplementary file 1 [file Data_Sheet_1.ZIP › Original IF images/ICH magnification × 100/LEF1/LEF1.jpg]

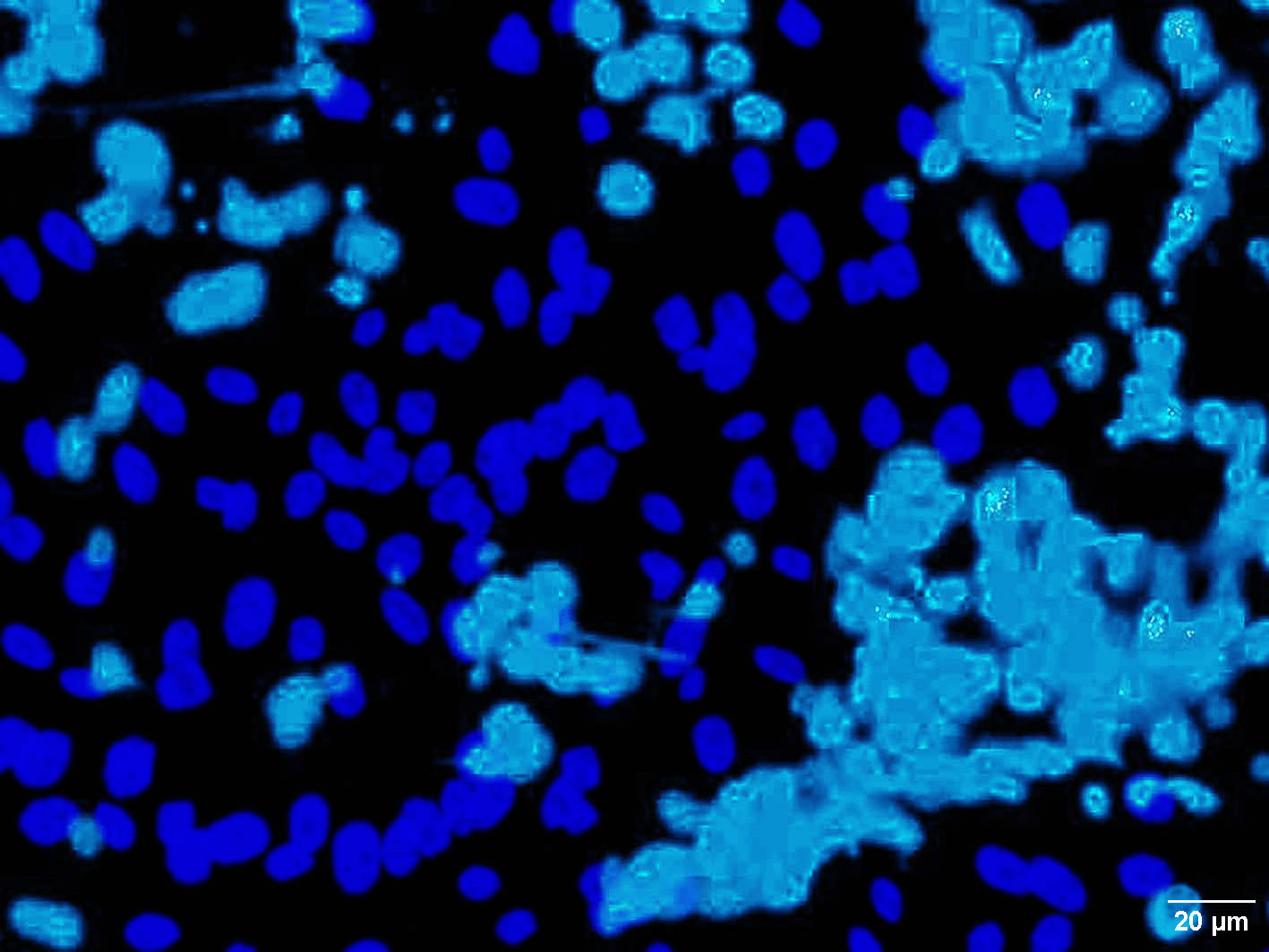

Supplement: Supplementary file 1 [file Data_Sheet_1.ZIP › Original IF images/ICH magnification × 100/LEF1/Merge.jpg]

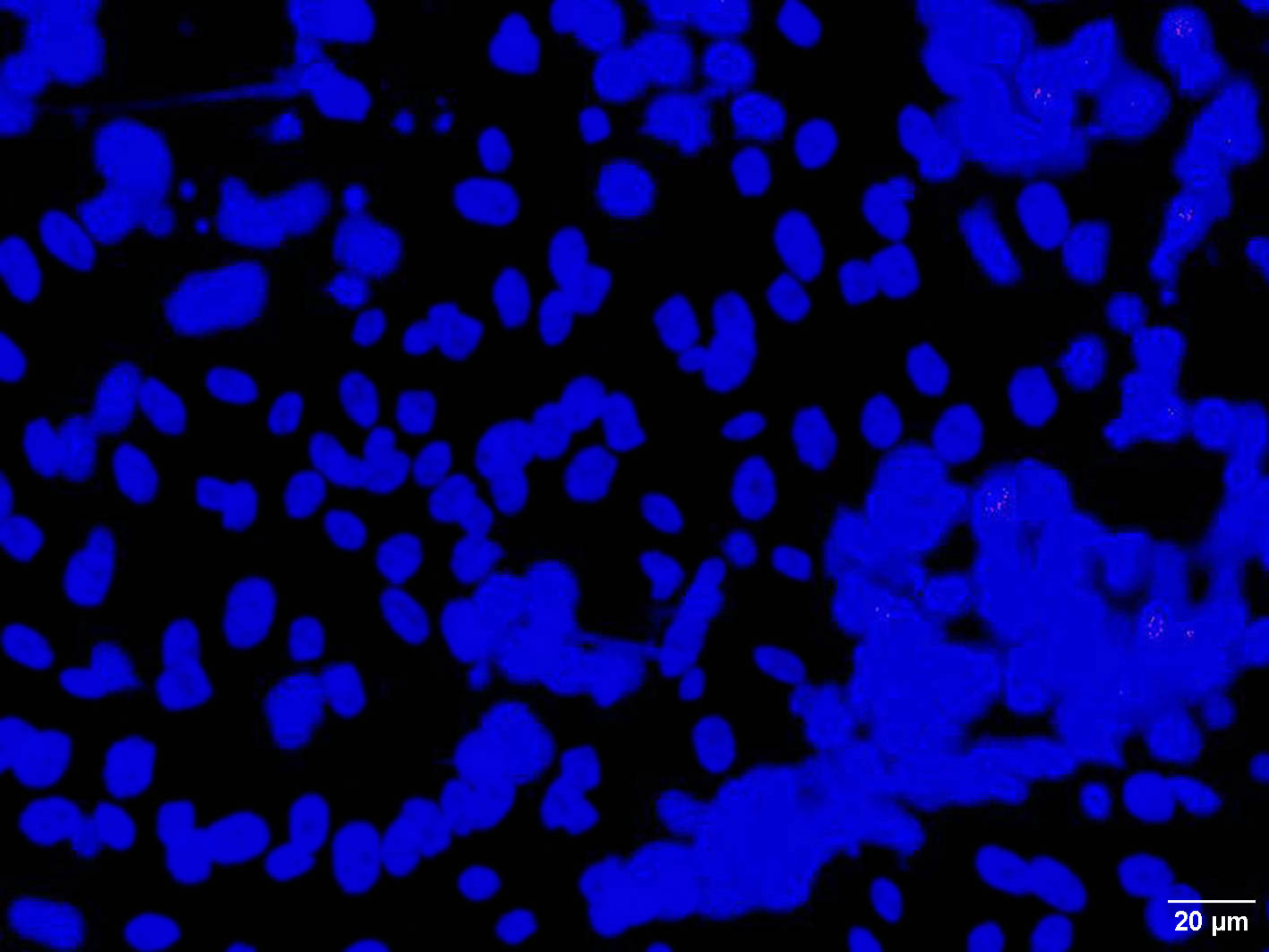

Supplement: Supplementary file 1 [file Data_Sheet_1.ZIP › Original IF images/ICH magnification × 100/LEF1/DAPI.jpg]

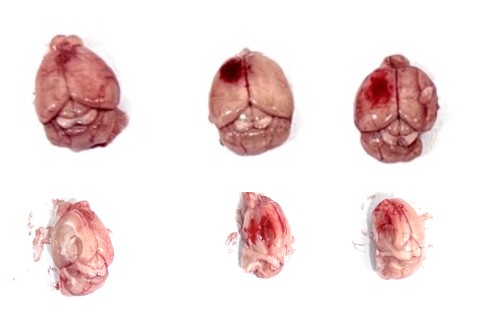

Supplement: Supplementary file 2 [file Data_Sheet_2.ZIP › Original photographs of animal brain tissues/Brain tissues of mice.jpg]

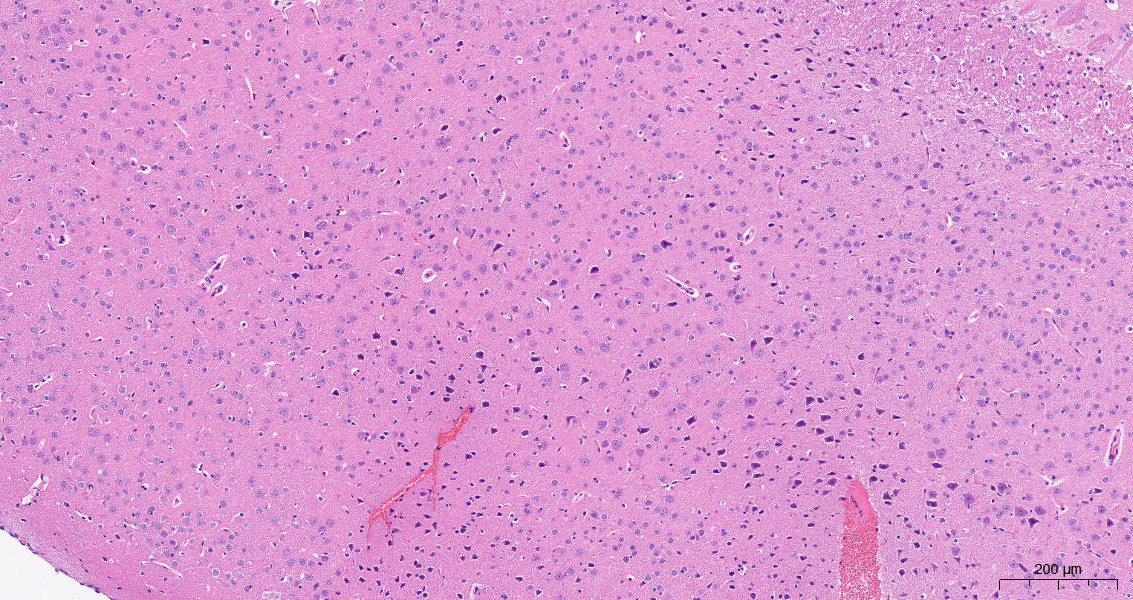

Supplement: Supplementary file 2 [file Data_Sheet_2.ZIP › Original photographs of animal brain tissues/ICH+FMT magnification × 10.jpg]

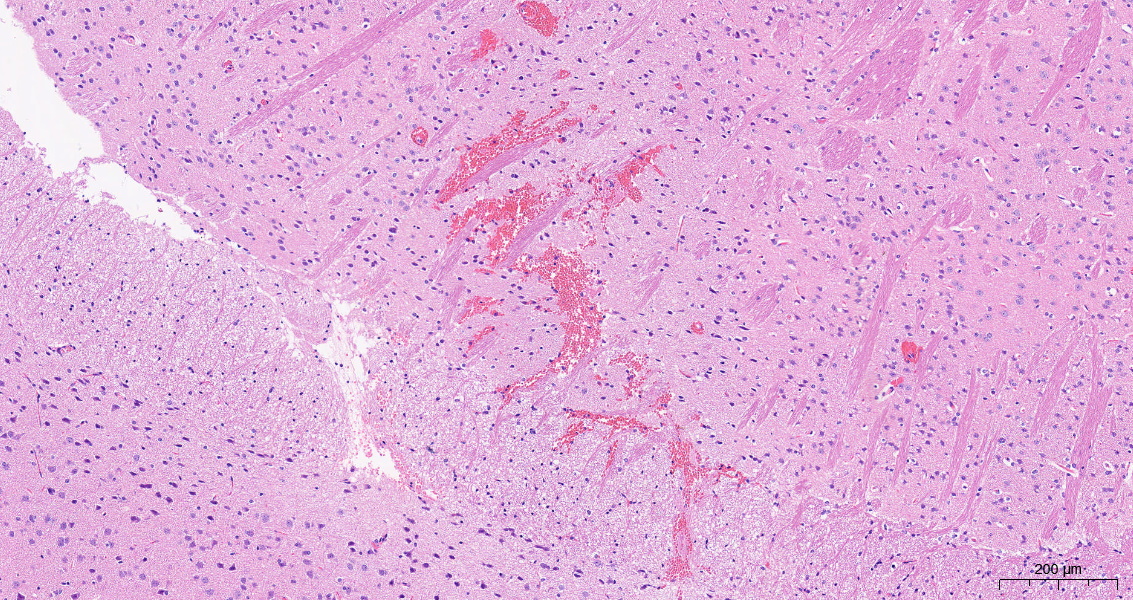

Supplement: Supplementary file 2 [file Data_Sheet_2.ZIP › Original photographs of animal brain tissues/ICH magnification × 10.jpg]

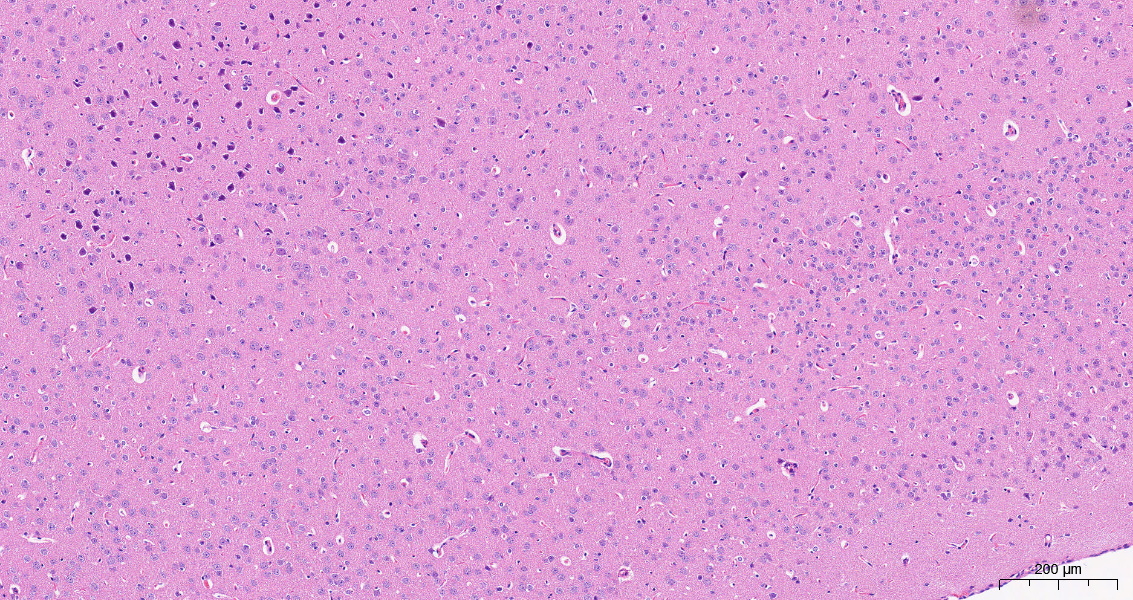

Supplement: Supplementary file 2 [file Data_Sheet_2.ZIP › Original photographs of animal brain tissues/Control magnification × 10.jpg]

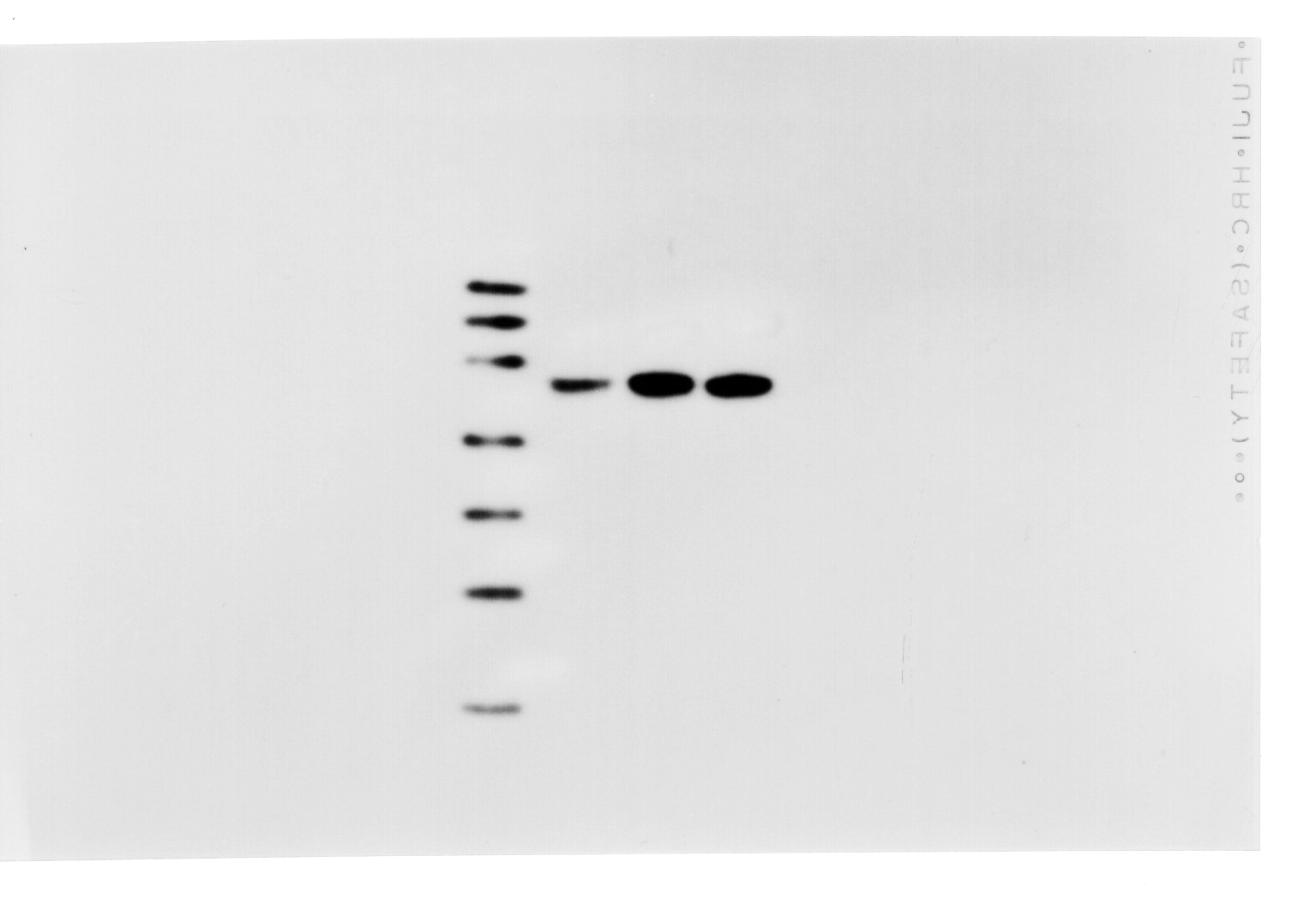

Supplement: Supplementary file 3 [file Data_Sheet_3.ZIP › Original WB/Figure 10H/ITGAX.jpg]

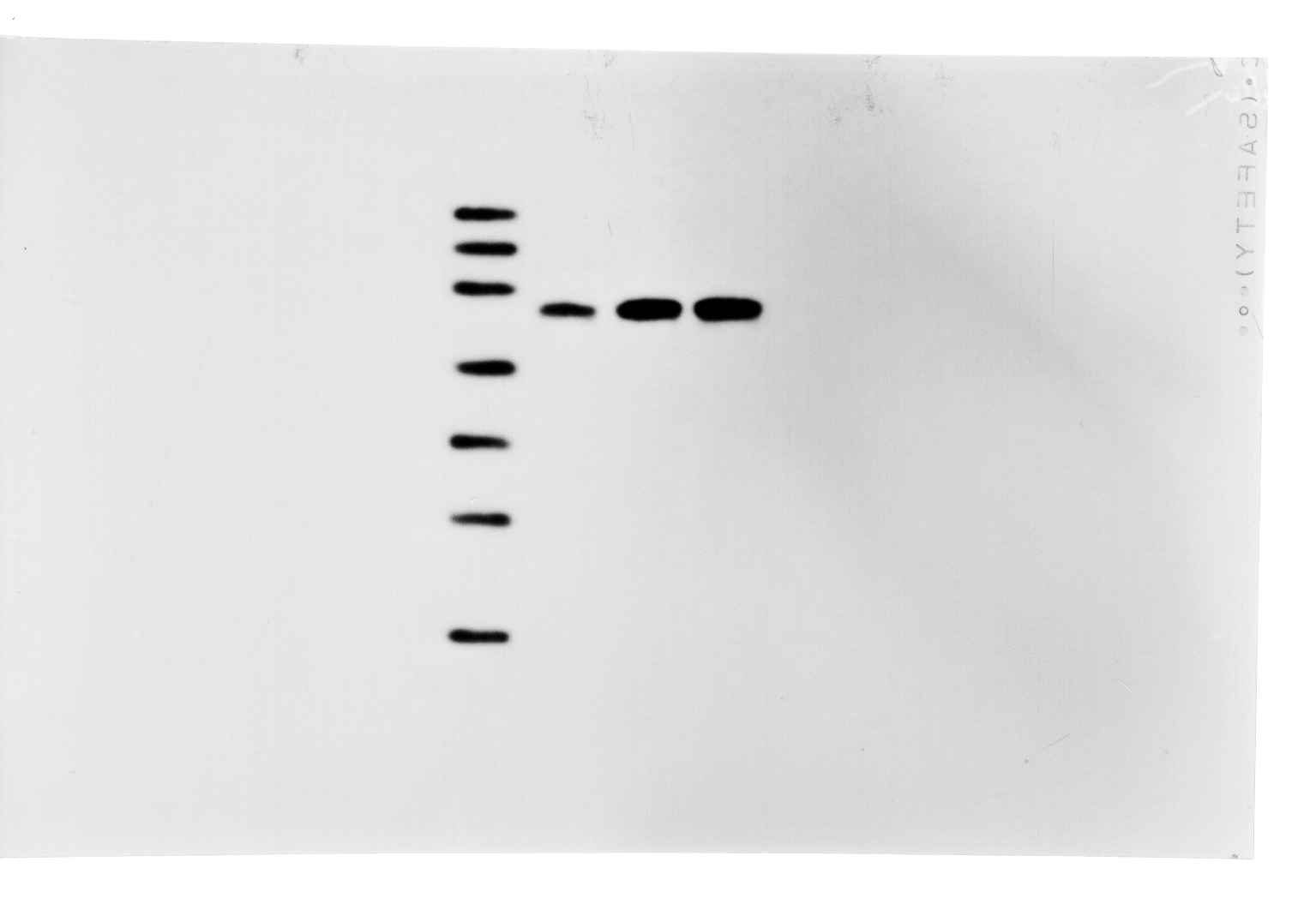

Supplement: Supplementary file 3 [file Data_Sheet_3.ZIP › Original WB/Figure 10H/ATF4.jpg]

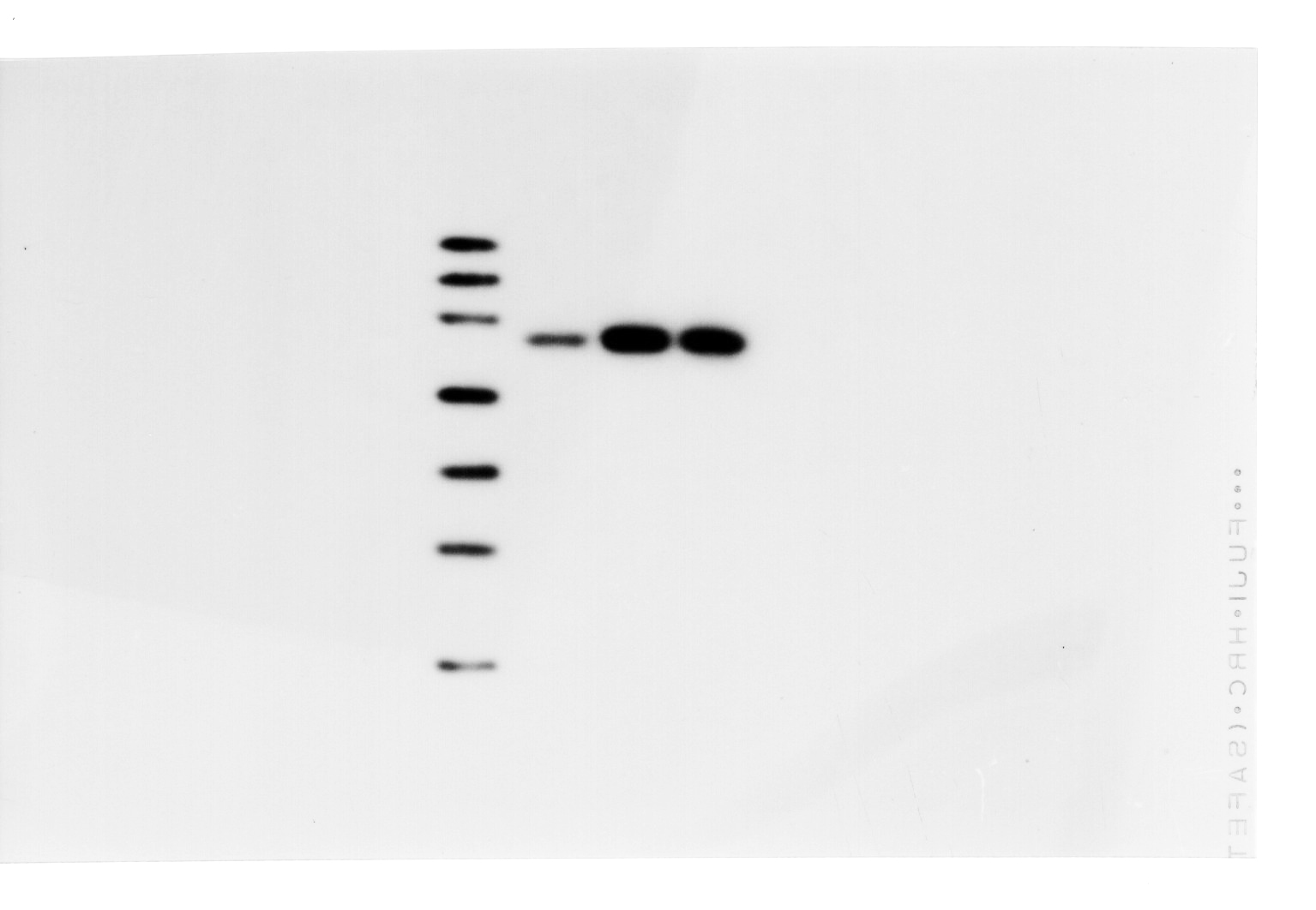

Supplement: Supplementary file 3 [file Data_Sheet_3.ZIP › Original WB/Figure 10H/LEF1.jpg]

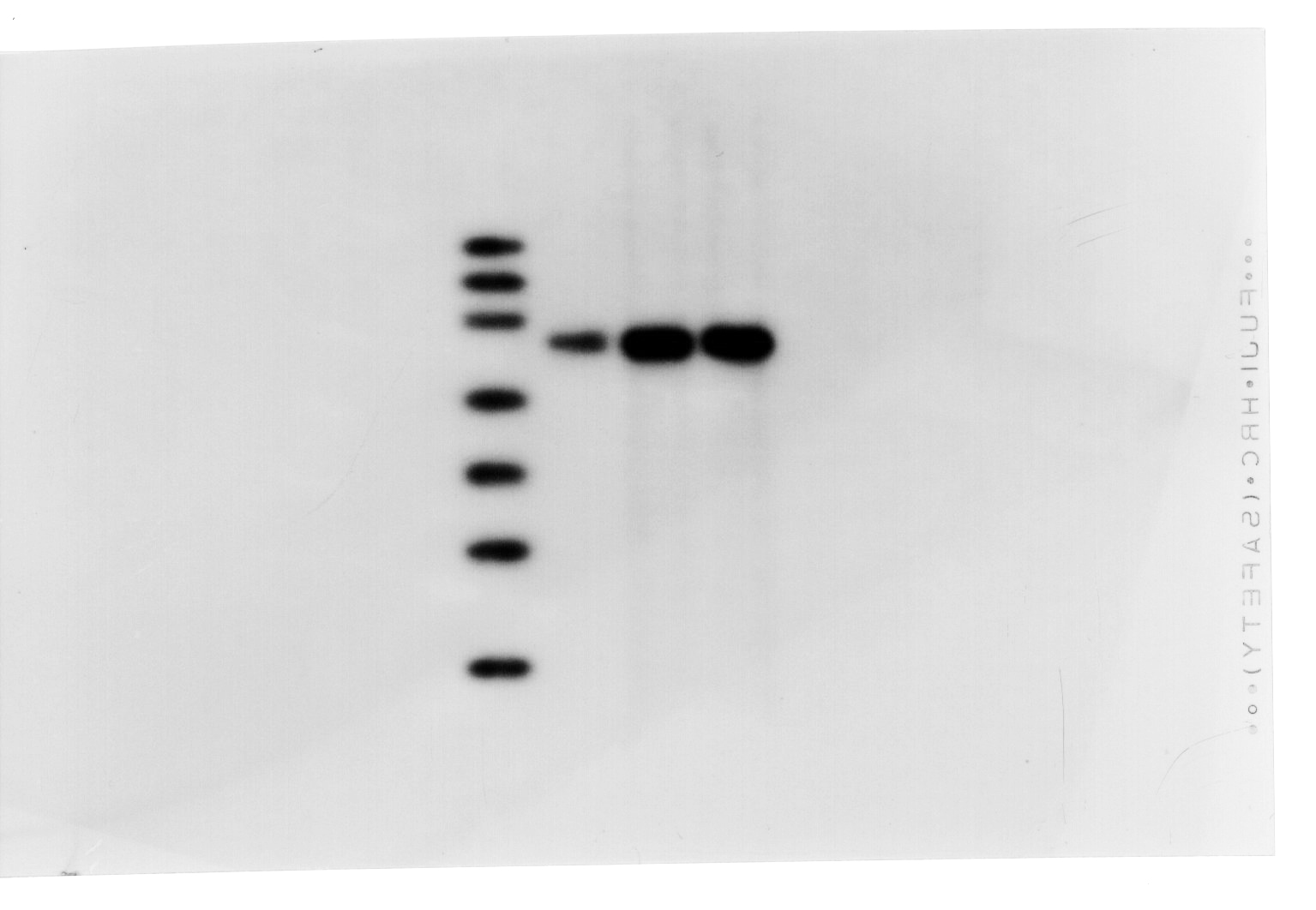

Supplement: Supplementary file 3 [file Data_Sheet_3.ZIP › Original WB/Figure 10H/BLVRB.jpg]

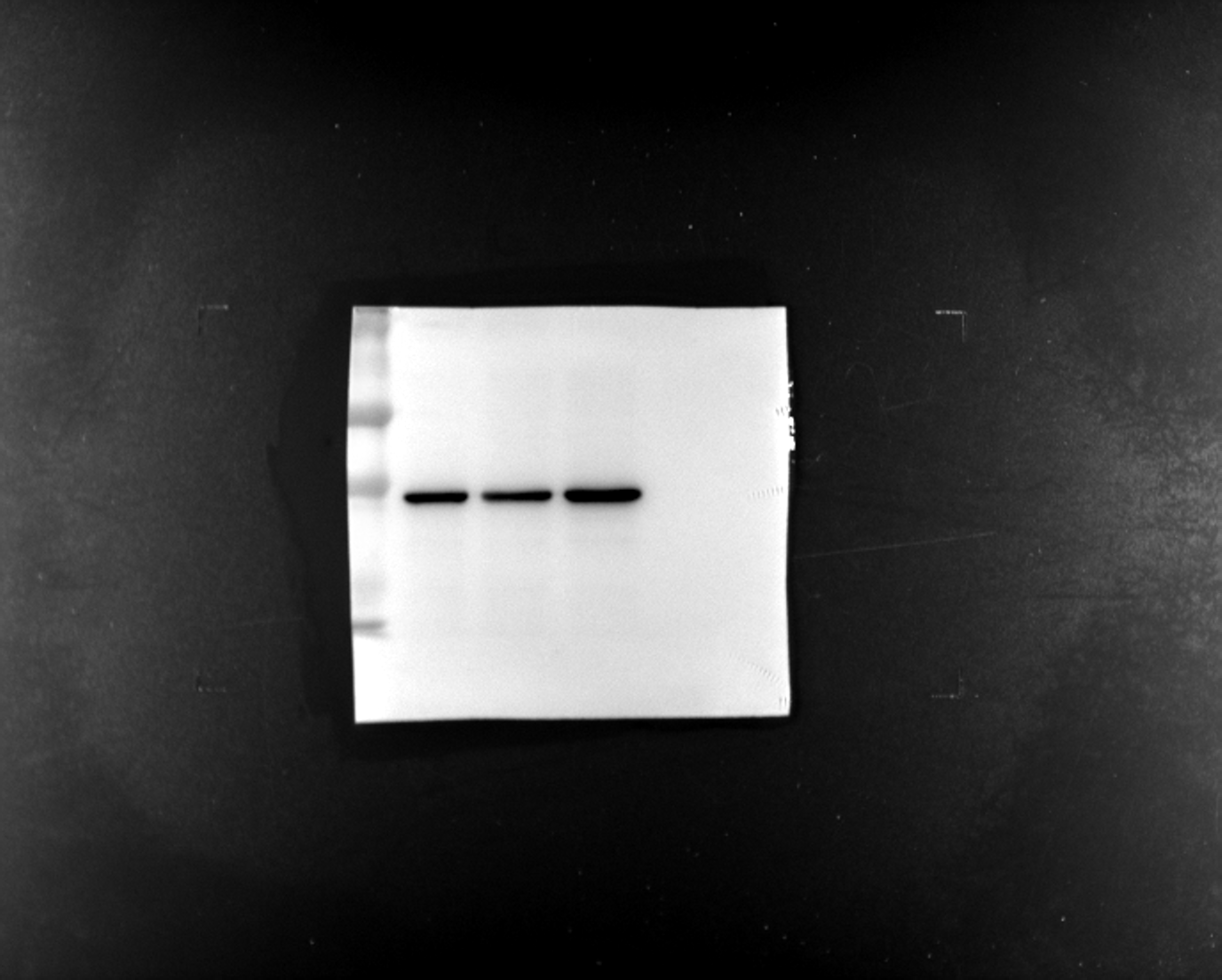

Supplement: Supplementary file 3 [file Data_Sheet_3.ZIP › Original WB/Figure 10H/GAPDH.Tif]

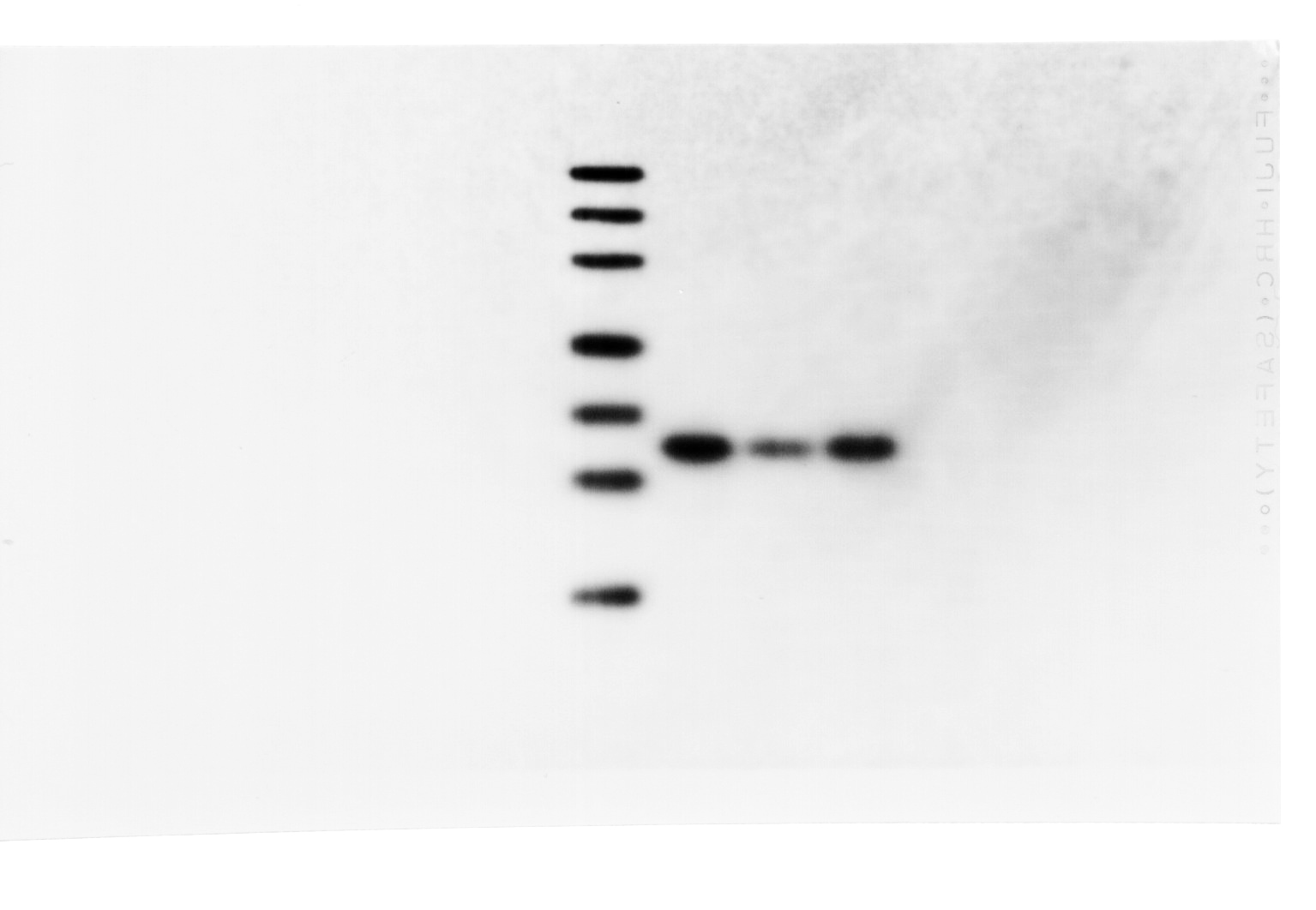

Supplement: Supplementary file 3 [file Data_Sheet_3.ZIP › Original WB/Figure 10F/Occludin.jpg]

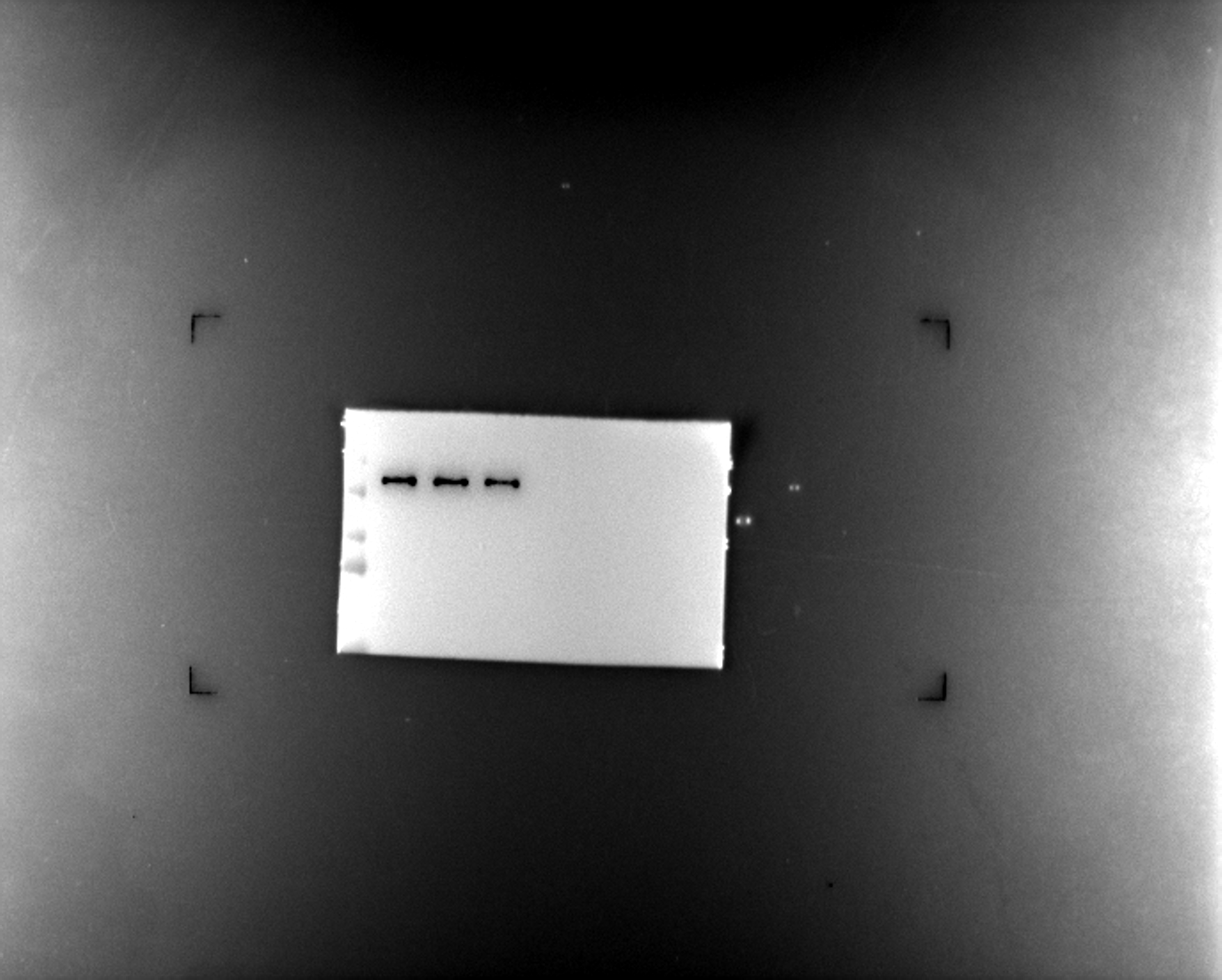

Supplement: Supplementary file 3 [file Data_Sheet_3.ZIP › Original WB/Figure 10F/GAPDH.Tif]

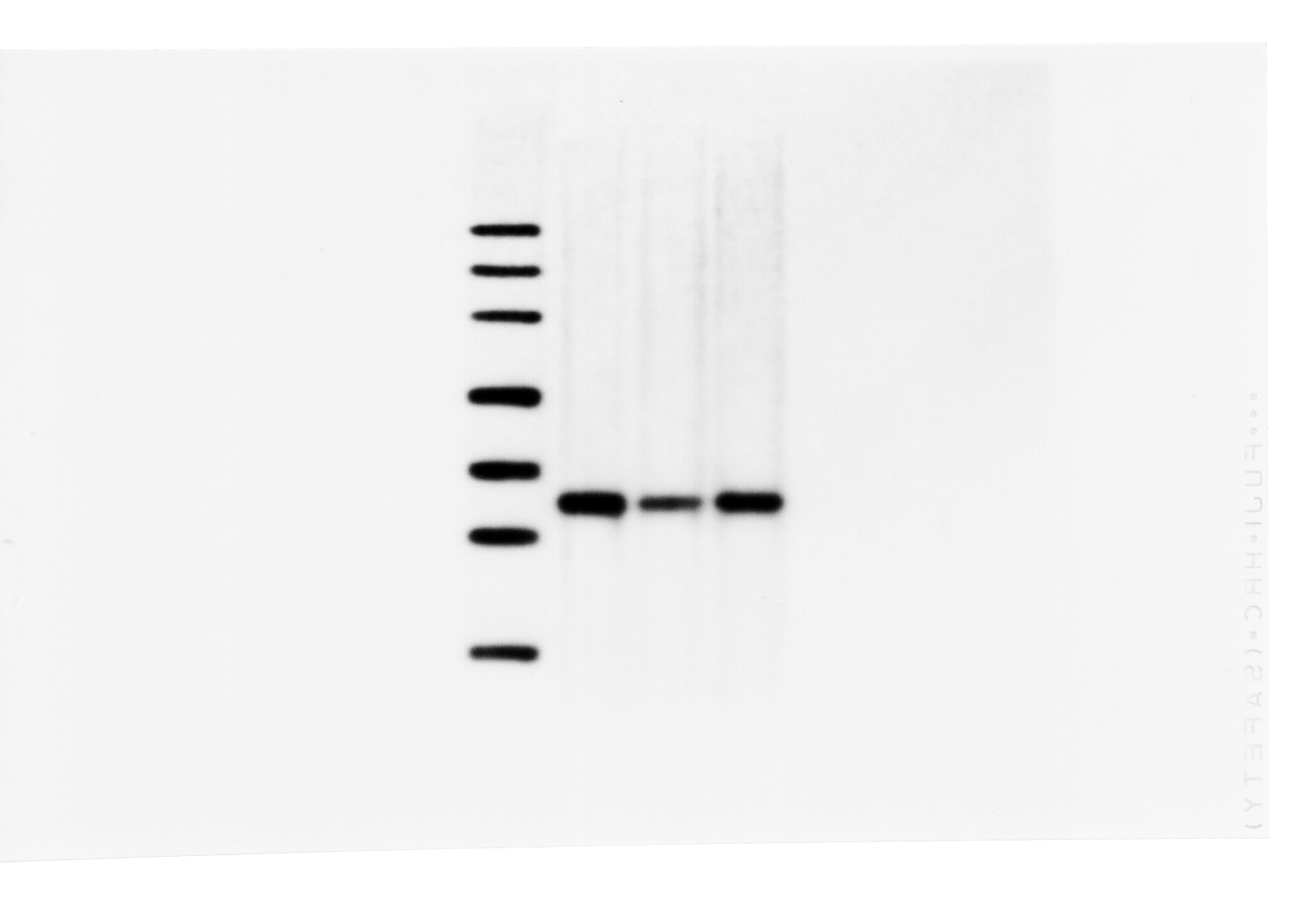

Supplement: Supplementary file 3 [file Data_Sheet_3.ZIP › Original WB/Figure 10F/ZO-1.jpg]

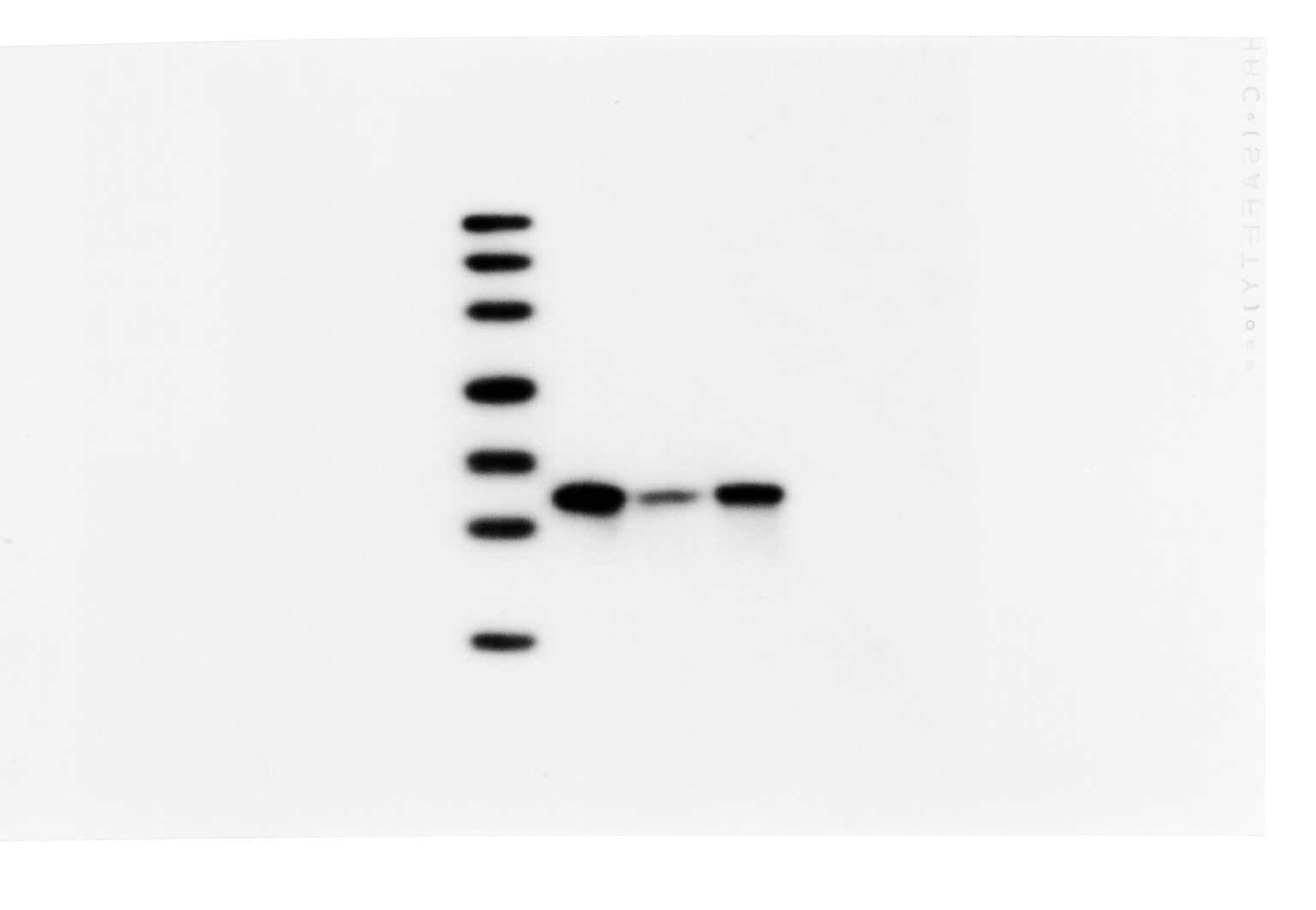

Supplement: Supplementary file 3 [file Data_Sheet_3.ZIP › Original WB/Figure 10F/Claudin-4.jpg]
